# Exploring the Chemistry of the Mechanical Bond: Synthesis of a [2]Rotaxane through Multicomponent Reactions

**DOI:** 10.1021/acs.jchemed.3c00163

**Published:** 2023-08-01

**Authors:** Adrian Saura-Sanmartin, Jorge Lopez-Sanchez, Carmen Lopez-Leonardo, Aurelia Pastor, Jose Berna

**Affiliations:** Departamento de Química Orgánica, Facultad de Química, Regional Campus of International Excellence “Campus Mare Nostrum”, Universidad de Murcia, E-30100 Murcia, Spain

**Keywords:** Upper-Division Undergraduate, Organic Chemistry, Hands-On Learning/Manipulatives, Noncovalent Interactions, Mechanical Bond, Rotaxanes, Supramolecular Chemistry

## Abstract

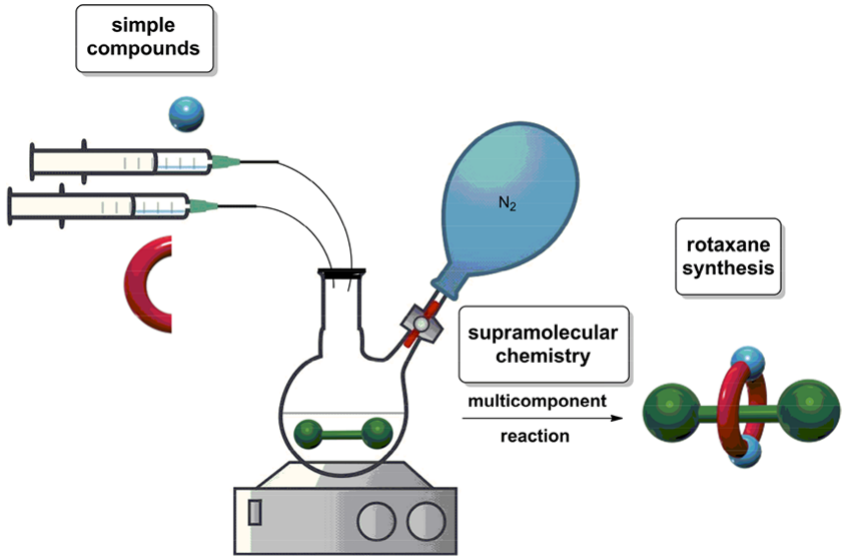

The synthesis of a [2]rotaxane through three- or five-component coupling reactions has been adapted to an organic chemistry experiment for upper-division students. The experimental procedure addresses the search for the most favorable reaction conditions for the synthesis of the interlocked compound, which is obtained in a yield of up to 71%. Moreover, the interlocked nature of the rotaxane is proven by NMR spectroscopy. The content of the sessions has been designed on the basis of a proactive methodology whereby upper-division undergraduate students have a dynamic role. The laboratory experience not only introduces students to the chemistry of the mechanical bond but also reinforces their previous knowledge of basic organic laboratory procedures and their skills with structural elucidation techniques such as NMR and FT-IR spectroscopies. The experiment has been designed in such a customizable way that both experimental procedures and laboratory material can be adapted to a wide range of undergraduate course curricula.

## Introduction

Mechanically interlocked molecules (MIMs) are constituted by at least two counterparts that are linked together mechanically. The mastering of noncovalent interactions between structurally programmed building blocks allows the construction of this appealing sccafolds.^1^ The new properties conferred by the mechanical bond to MIMs have resulted in exciting applications, contributing to the research in this burgeoning field.^1^ In fact, the synthesis and study of this type of structure constituted part of the topic of the Nobel Prize in 2016.^2−4^

**Figure 1:**
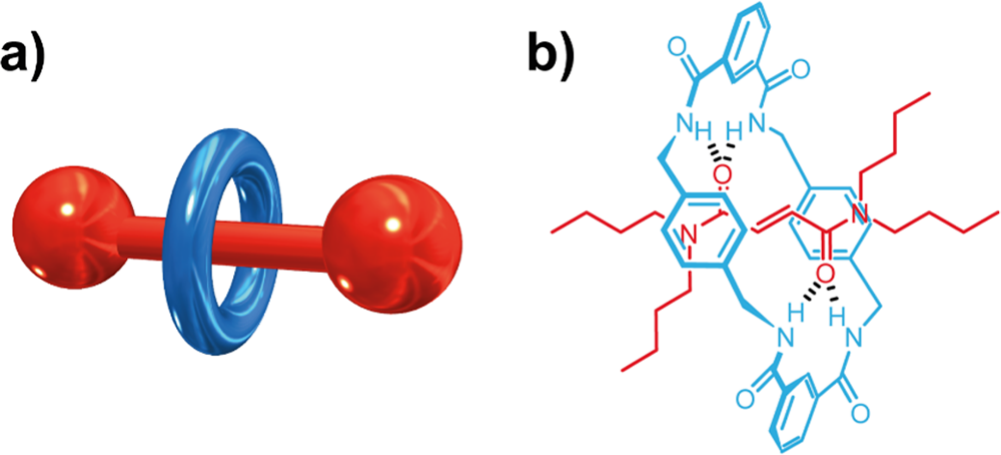
(a) Cartoon representation of a [2]rotaxane showing the dumbbell-shaped component in red and the cyclic one in blue and (b) chemical structure of a Leigh-type [2]rotaxane (**1**).

The two archetypal examples of MIMs are rotaxanes and catenanes.^1^ [2]Rotaxanes are molecules having a dumbbell-shaped component surrounded by a cyclic one (Figure 1a). They have emerged as useful scaffolds for constructing different molecular architectures, such as catalysts,^5,6^ smart materials^7^ and molecular machines.^8^ The experiment described herein is focused on the synthesis of a Leigh-type rotaxane (Figure 1b).^9^

Despite the scientific relevance of MIMs, their synthesis is challenging.^10^ The most effective protocols are based on the formation of a supramolecular intermediate that allows an efficient orientation between the precursors through molecular recognition (template effect).^11^ Hence, supramolecular chemistry is key in the synthesis of MIMs in general and [2]rotaxanes in particular. Nevertheless, the undergraduate curriculum often leaves supramolecular chemistry behind, even though it is one of the hot topics of research in chemistry.^12−14^ In this context of underrepresentation, MIMs^4^ have been rarely disclosed in chemical education literature, except for one example about the synthesis of Borromean rings by students,^15^ and a few reported experiments in this Journal employing supramolecular chemistry^16,17^ and multicomponent reactions.^18,19^

Herein, we described a modification of our previously reported experimental procedure for the synthesis of the fumaramide-based [2]rotaxane **1** (Figure 1b)^20^ to turn it into a customizable laboratory activity suitable for upper-division undergraduate students. Both, the appealing geometry of rotaxanes and interesting properties conferred by the mechanical bond, will provide significant enrichment to the laboratory experience and raise student’s interest and engagement. Notably, the designed experiment combines synthetic organic chemistry, supramolecular chemistry, and structural elucidation. This conjunction turns this protocol into a very instructive experience, in which students can improve their previous knowledge and technical skills in synthetic organic chemistry.

## Pedagogical Significance

The pedagogic goals established for this laboratory activity are (a) to introduce students to the chemistry of the mechanical bond through the synthesis of a benzylic amide [2]rotaxane; (b) to reinforce the concepts related to supramolecular chemistry and the key role of the template effect in the synthesis of MIMs; (c) to strengthen their technical skills in synthetic organic synthesis; (d) to solidify their expertise in structural identification of organic compounds, in particular mechanically interlocked compounds, through the analysis of NMR and FT-IR spectroscopic data; and above all (e) to compare and interpret a set of results obtained under different reaction conditions on the basis of the provided background.

The preparation of the teaching material to meet these pedagogic goals can be addressed very effectively by involving students in the process. In this way, the instructors have a more accurate idea of their strengths and weaknesses, allowing them to focus on topics that require greater emphasis. The approach described herein allows the assessing of a diversity of laboratory competencies related to the experimental work through the exercising of analytical, manipulative practical and instrumental skills and also the so-called soft skills (critical thinking, teamwork, and written communication skills).^21^

## Experimental Overview

The laboratory experiment comprises the synthesis of benzylic-amide-based [2]rotaxane **1** by using two different methods. Both methods are based on the formation of amide bonds by reacting amines with acid chlorides in the presence of a base.^22−25^ This simple reaction allows students to focus their attention on understanding the concepts related to the molecular recognition event and the template effect as the main contribution for the successful synthesis of interlocked compound **1**.

**Figure 2:**
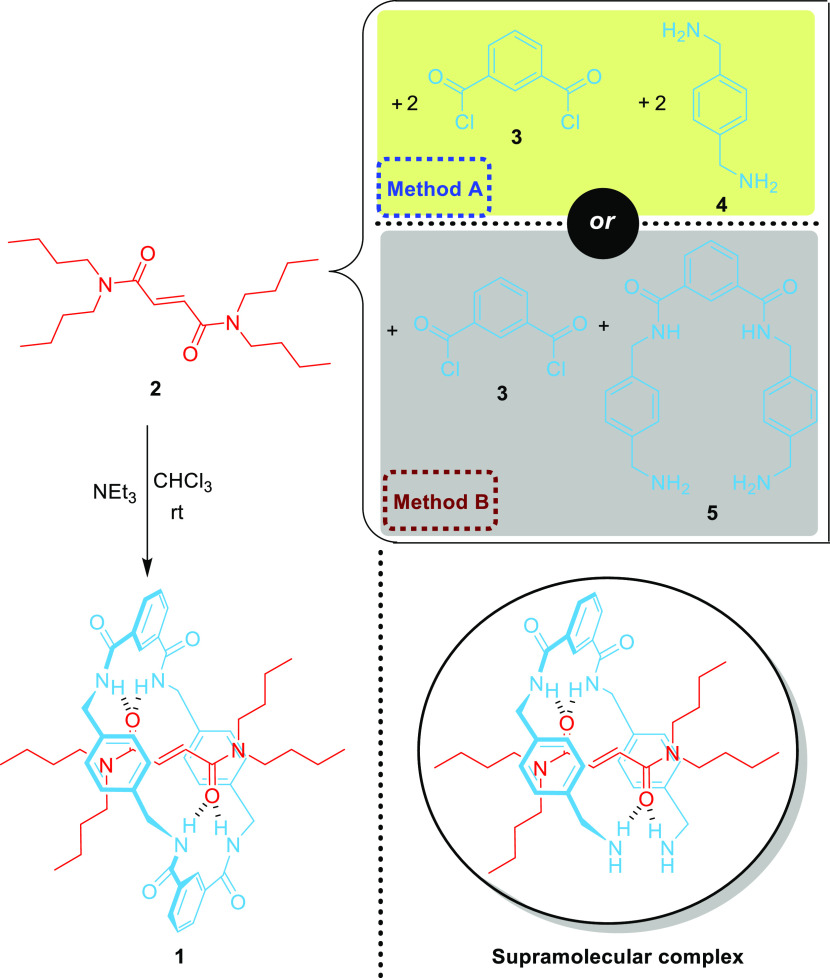
Synthesis of [2]rotaxane **1**. Method A: five-component coupling reaction involving thread **2**, two equiv of diacid chloride **3** and two equiv of diamine **4**. Method B: three-component coupling reaction involving thread **2**, diacid chloride **3** and diamine **5**. Macrocycle and reactants leading to it are shown in blue, and thread is shown in red. The supramolecular complex prior to the formation of the rotaxane is drawn at the bottom right.

The first protocol is based on the reaction of *N*^1^,*N*^1^,*N*^4^,*N*^4^-tetrabutylfumaramide (**2**) with two molecules of isophthaloyl dichloride (**3**) and other two of *p*-xylylenediamine (**4**) (Figure 2, Method A).^20^ The formation of benzylic amide-based [2]rotaxane **1** through this methodology proceeds with the formation of U-shaped diamide precursor **5**. The structural complementarity between this U-shaped intermediate and the thread favors the formation of a supramolecular structure stabilized by four hydrogen bonds, established between the carbonyl groups of the thread and the NH groups of the other counterpart (Figure 2, bottom right). The formation of this supramolecular complex arranges the components in the proper orientation and favors the formation of the mechanically interlocked structure. The subsequent reaction with another molecule of isophthaloyl chloride leads to the target interlocked product **1**.^26^

Method B is based on the direct reaction of the previously synthesized U-shaped diamide **5** with isophthaloyl chloride (Figure 2, Method B). In this three-component coupling reaction, the direct formation of the supramolecular complex is facilitated, and thus, an improvement in the yield of the intertwined species is expected. The importance of the supramolecular complex in the formation of rotaxane **1** can be substantiated with the obtained yields by using both methodologies.

To favor the formation of the supramolecular complex and thus, avoid the formation of polyamides and other byproducts resulting from the direct reaction of isophthaloyl dichloride and *p*-xylylenediamine (macrocycle and catenane; see page S16 in the SI Instructor notes), both coupling reactions must be carried out under high dilution conditions and by slow addition of the reactants.^26,27^ An array of different concentrations and reactant addition rates is employed in both methods, A and B, to illustrate the influence of both factors.

After the synthesis and isolation steps, the structural characterization of [2]rotaxane **1** is addressed by comparing its ^1^H NMR spectrum to that of thread **2**. In particular, attention is focused on the diamagnetic shielding experienced by the olefinic protons of the fumaramide of the thread placed at the void of the benzylic amide macrocycle within **1**. 1D- and 2D-NOESY experiments allow to unequivocally conclude the successful formation of the intertwined species.

## Hazards

Students must wear a lab coat and safety goggles before entering the lab. Additionally, they are provided with gloves. They must wear these safety elements during all of the experiment. Hydrochloric acid and sodium hydroxide are corrosives and can cause severe skin burns and eye damage. Furthermore, hydrochloric acid is toxic if inhaled. For this reason, the preparation of 1 M solutions of these chemicals should be carried out in a fume hood. Similarly, due to the toxicity of chloroform and diethyl ether, as well as some other reactants, such as isophthaloyl dichloride and triethylamine, all chemicals must be handled in a well-ventilated fume hood (see the Supporting Information (SI) Student handout draft). Indeed, students are warned of the toxicity of these compounds during the sessions as well as the danger regarding eye and skin contact and inhalation. Safety information for all reagents is available via the appropriate Safety Data Sheets. The Chemical Abstracts Service (CAS) Numbers and Globally Harmonized System (GHS) Hazards of the chemical compounds and solvents employed for these experiments can be found in the SI.

## Practical Considerations

The material described herein was prepared with the involvement of students who had an upper-division internship in the Department of Organic Chemistry (Universidad de Murcia, Spain). The internship is optional and comprises up to 30 h per year granting participants a certificate, which is included as an additional merit in their academic records. Throughout the internship, students have the opportunity to put classroom theory into practice through specific research and laboratory experiments.

The preparation of the material was carried out following the steps indicated below: (i) definition of pedagogical objectives; (ii) preparation of theoretical and experimental protocol drafts that are distributed to the participating students (see the SI Student handout draft); (iii) development of the experimental work by the undergraduate students under the supervision of the instructors; and (iv) modification and validation of the content for the laboratory (see the SI Student handout modified on the basis of students’ suggestions and direct observation by the instructors).

This experiment has been designed to be carried out in three sessions of 3 h each in the context of the optional internship (see above) with up to 6 students each term when syringe pumps are employed for the slow addition of the reagents. Nevertheless, when syringe pumps are replaced by dropping funnels, the number of students could be increased to 12 students per session, though the second session would require 6 h. The organization of the experimental sessions, including the key points for their correct implementation, is specified below. From a practical point of view, the number of injection pumps for the slow addition of the reagents (see below) is a limiting factor. For that reason, the students can do the experimental work in small groups. Nevertheless, all of the students should analyze the spectroscopic data by themselves, and the learning assessment of every student should be carried out individually.

Thread **2** and precursor **5** are not commercially available; therefore, these compounds should be prepared in advance by the students or instructors following the protocols reported in the SI Instructor notes.

### Seminar Session

One week before the seminar session, the instructor sends to the students the “Student handout” by e-mail, containing a background, bibliographic information, and the prelabs. Students are expected to work on it and submit them during the next session for group assessment.

During the first session, the instructor gives a short talk aided by a slides-based presentation focused on basic features of mechanically interlocked molecules. The presentation emphasizes the points indicated below:

- Definition of mechanical bond and main types of MIMs. In this part, the instructor should explain the difference between MIMs and supramolecular complexes.
- Dynamics and selected applications of rotaxanes. This part is the most appealing one and should encourage students to accomplish the experiment. The selected examples should be grounded in the chemistry knowledge that they have acquired during their undergraduate curriculum.
- Synthetic methodologies to obtain rotaxanes. In this section, the key role of the template effect and the formation of a supramolecular complex between the thread and the U-precursor, which finally leads to the intertwined product, should be highlighted.
- Alternative synthetic methods, A and B, which furnish the fumaramide-based [2]rotaxane **1**. In this part, the mechanism of the multicomponent coupling reactions should be explained.
- Crucial aspects to consider during the laboratory experiment.

During the session, the prelab activities are discussed with the instructor (see section 9 of the SI Student handout modified). Later on, the hazards of selected chemicals are emphasized (all safety and hazard considerations are listed in section 5 of the SI Instructor notes). Finally, some key concepts of spectroscopic techniques are revised, taking the ^1^H and ^13^C NMR spectra of thread **2** as an example.

### First Laboratory Session

During this session, the instructor assigns to every student different procedures (method A, five-component coupling reaction or method B, three-component coupling reaction) for the preparation of the fumaramide-based [2]rotaxane **1**. The procedures given to the students not only differ in the structure of the reagents but also in their addition times and concentrations.

**Figure 3:**
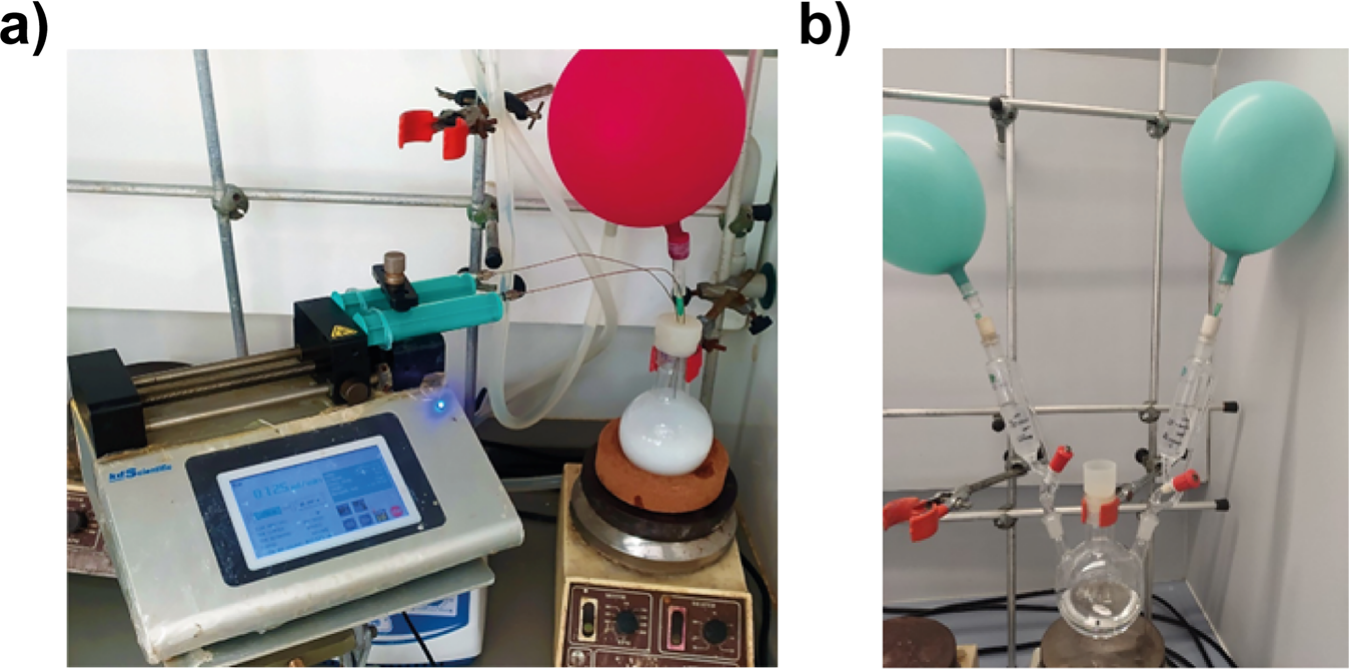
Experimental setups for the synthesis of [2]rotaxane **1**, consisting of: (a) a motor-driven syringe pump, a round-bottom flask, a septum, two syringes, two bent needles, a magnetic stir plate, a nitrogen filled balloon, and an elevator (the picture was taken almost at the end of the addition time, showing a white precipitate that corresponds to polymeric byproducts); and (b) a round-bottom three-neck flask equipped with two addition funnels and two nitrogen balloons (the picture was taken before the addition of the solutions of the reactants).

In method A, thread **2** and Et_3_N are dissolved in CHCl_3_ and stirred vigorously under an inert atmosphere, while solutions of (i) *p*-xylylenediamine **4** and Et_3_N and (ii) isophthaloyl dichloride **3**, both in the same solvent, are simultaneously added by using a motor-driven syringe pump or two independent addition funnels. At this point, the instructor underlines the importance of accurately assembling the setup including the proper position of the two syringes and the right addition flow rate (Figure 3).

Method B comprises the vigorous stirring of a solution of thread **2**, Et_3_N and the precursor **5** in CHCl_3_ under an inert atmosphere while a solution of isophthaloyl dichloride **3** in the same solvent is added using a motor-driven syringe pump.

After the slow addition of the reagents, the workup is similar in both methodologies and consists on filtration of the reaction mixture through a Celite pad,^28^ and sequential washing of the filtrate with water, 1 M HCl solution, 1 M NaOH solution and brine. The organic phase is then dried over MgSO_4_, filtered, and the solvent is removed under reduced pressure. The resulting solid is washed with diethyl ether until all the unreacted thread is removed^29^ and then dried under vacuum (for more details, see the SI).

After this stage, the students should perform the structural characterization of the interlocked product **1**, by measuring its melting point, recording the FT-IR spectrum of a nujol mull, and preparing an NMR sample in CDCl_3_, which will be recorded by the technical staff.

The students are reminded during the reaction times as well as any other timeouts about some key concepts of spectroscopic techniques, highlighting those related to NMR and, particularly, 2D experiments such as COSY and NOESY.^30^ It should be noted that these concepts do not go into theoretical details but are focused on a mere interpretation of the spectra. Thus, practical knowledge of structural elucidation is intended, not a full understanding of each one of the NMR experiments.

### Second Laboratory Session

The students receive the NMR spectra of their products beforehand so that they can examine their own data, which include ^1^H, ^13^C, DEPT, APT, COSY, HSQC, HMBC, and NOESY spectra (see the SI Instructor notes for a full assignment of the signals). At this point, the student should be able to give an almost full assignment of the ^1^H and ^13^C signals. Nevertheless, key points of each NMR experiment^30^ are discussed during this session for guiding students in the signals’ assignment. Finally, typical features showed by MIMs, e.g., how the encapsulation of the thread inside the cavity of the macrocycle can be detected by the shifts of selected resonances, are explained to the students.

After the data analysis of rotaxane **1**, the students report their reaction yields while describing the reaction conditions used to prepare it, and all the relevant details are organized in a table. With all the data in hand, every student should draw their own conclusions and be able to answer a key question through a guided-inquiry process: how do the addition time and the dilution conditions affect to the formation of benzylic amide rotaxanes?

## Results and Discussion

### Experiment Performance

With all the information provided in Table 1, students can identify key elements that have the greatest impact on the yield of **1**, i.e., slow addition and high dilution, which in turn are the reaction conditions which favor the formation of the supramolecular complex.

**Table 1:** Selected Screening Data for the Synthesis of [2]Rotaxane **1**

Entry	**2** (mmol)	**3** (mmol)	**4** (mmol)	Et_3_N (mmol)	CHCl_3_ (mL)	Addition time (min)	Yield of **1** (%)	Addition method
1	0.59	4.72	4.72	14.16	180	30	29–33	Syringe pump
2	0.59	4.72	4.72	14.16	180	60	37–39	Syringe pump
3	0.59	4.72	4.72	14.16	180	90	40–42	Syringe pump
4	0.59	4.72	4.72	14.16	360	90	69–71	Syringe pump
5	0.59	4.72	4.72	14.16	360	240	64–67	Dropping funnel

Relevant details for the students are (i) larger addition times enhance the yield; (ii) larger reaction volumes also improve the yield; and (iii) when comparing methodologies A and B, the use of the U-shaped precursor **5** leads to a notable improvement of the yield of the reaction (see Tables S3 and S4 of the SI Instructor notes).

At this point, it is worth noting that the implementation of method A (five-component coupling reaction) turns out to be more feasible compared to method B, due to the synthetic steps required to prepare compound **5**, which is not commercially available. For pedagogical and practical purposes, we recommend employing the reaction conditions outlined in Table 1, which are based on method A (for a complete picture, see the SI, where the whole set of experiments based on methods A and B have been included).

The array of experiments depicted in Table 1 clearly shows the enhancement of the yield (from 29 to 33% to 40–42%) by increasing the reaction time from 30 to 90 min (Table 1 entries 1–3). Another interesting observation is that larger volumes (360 mL instead of 180 mL) also lead to better yields (69–71%). The use of a dropping funnel instead of an addition pump results in longer reaction times due to the decreased accuracy of the latter in controlling the added volume. However, comparable results can be obtained when a total volume of 360 mL of CHCl_3_ is used (entries 4 and 5). Curiously, the use of a dropping funnel also led to a better yield when the reaction takes place with less solvent (results shown in the SI). These results are rationalized as a consequence of the use of longer reaction times. Even the dropping funnel present a good alternative, we recommend the use of automatic syringe addition pumps to increase the instructor–student feedback during sessions.

Narrowing down the experimental procedure to the exclusive performance of the reaction conditions detailed in entries 4 and 5 (Table 1) turned out to be advantageous. By adopting this strategy, all students conduct the same experiments and the evaluation can be focused on the determination of the interlocked nature of the molecule, as well as in the improvement of their laboratory skills.

In the following session, the students determine, guided by the instructor, the interlocked nature of the product through the analysis of the available spectroscopic data. Interestingly, the IR band associated with the carbonyl groups of the fumaramide appears at 1621 cm^–1^ in thread **2**, while this value is reduced by 25 cm^–1^ in [2]rotaxane **1** as a consequence of the hydrogen bonds established with the amide protons of the macrocycle.^31−34^

**Figure 4:**
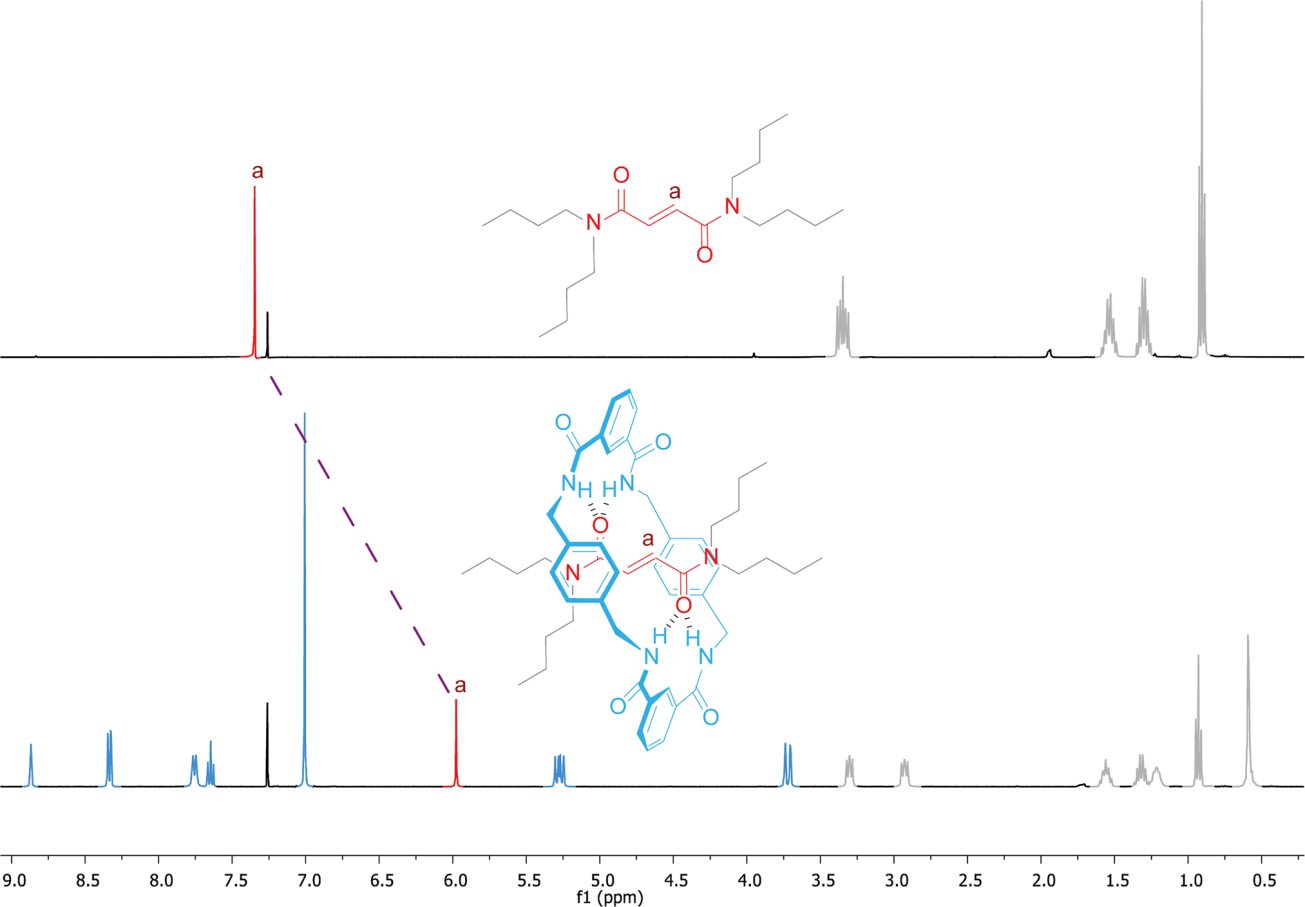
Stacked ^1^H NMR spectra (400 MHz, CDCl_3_, 298 K) of thread **2** (top) and [2]rotaxane **1** (below). The signals of the macrocycle are colored in blue, that of the methinic of the fumaramide is colored in red and those corresponding to the butyl chains are colored in gray. The dashed purple line connects the fumaramide signals (H_a_) of both spectra, allowing the chemical shift of this signal to be compared in the presence or absence of the mechanical bond.

Regarding the NMR data, the students should focus on the fumaramide singlet (H_a_) both in the spectrum of starting thread **2** and in that of rotaxane **1**. For this purpose, they have previously made an assignment using all of the provided spectra (see SI Instructor notes for a complete assignment of the NMR signals). Not surprisingly, students found it challenging to establish a relationship between the shielding experienced by the fumaramide signal of **1** (s, 5.98 ppm, 2H, Ha) in comparison with that of thread **2** (s, 7.35 ppm, 2H, Ha) and the formation of the mechanical bond (Figure 4). Some hints on concepts related to anisotropy in aromatic rings help them to reach this conclusion.^35,36^ After these indications, the undergraduate students associate the diamagnetic shielding of H_a_ in product **1** (Δδ = 1.37 ppm) to the fact that the tetralactam macrocycle is placed over the fumaramide.

**Figure 5:**
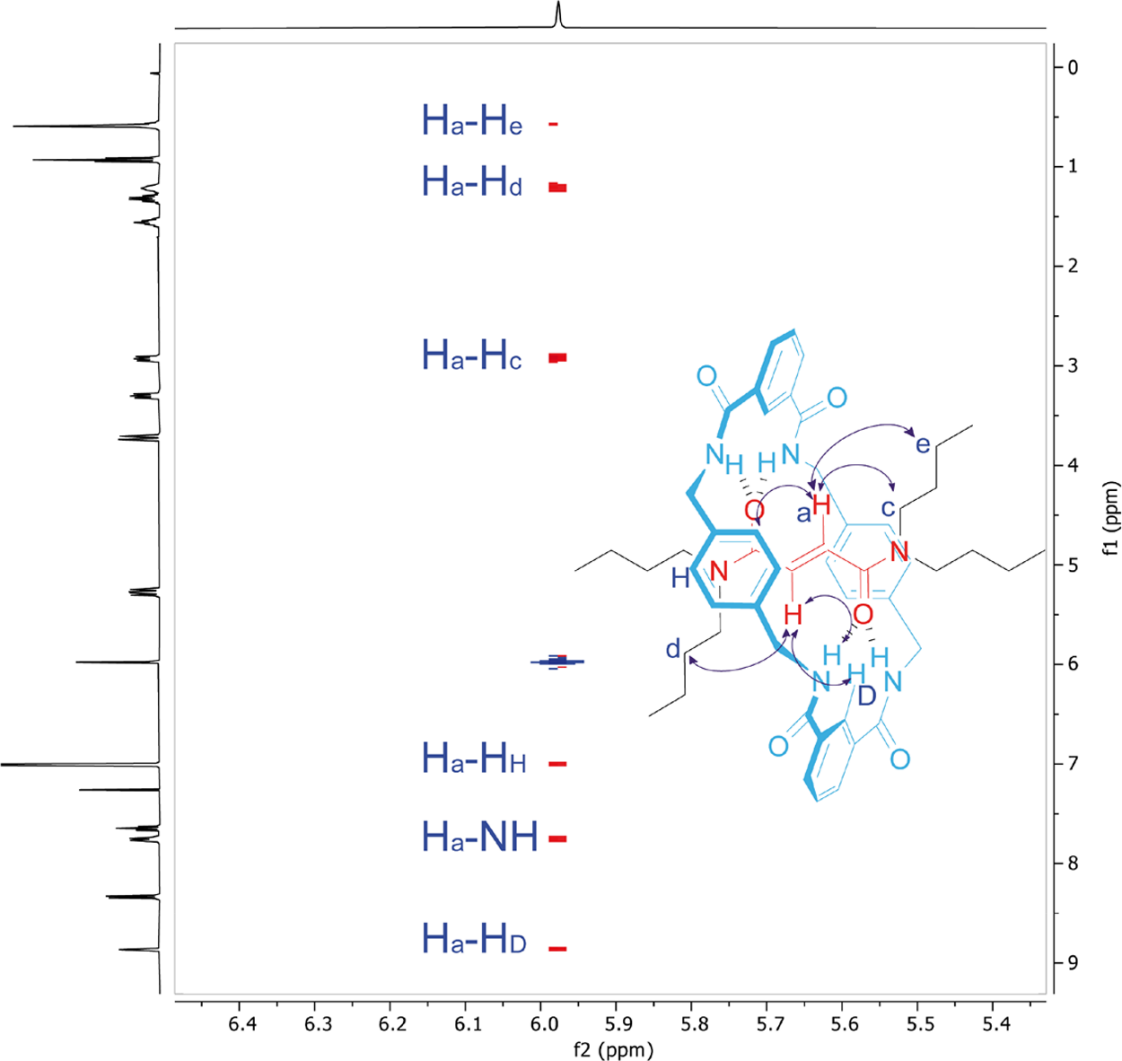
Partial ^1^H,^1^H-NOESY NMR spectrum (400 MHz, CDCl_3_, 298 K) of [2]rotaxane **1**. The spatial couplings between the linear and cyclic counterparts are indicated by using the assigned labeling shown in the structure. For clarity, the spatial couplings in the structure are indicated with only one of the equivalent protons.

Additionally, selected spatial couplings in the NOESY spectrum (400 MHz, CDCl_3_, 298 K) allow them to confirm the presence of the mechanical bond (Figure 5). Thus, cross peaks relating the fumaramide signal (H_a_) and selected protons of the tetraamide macrocycle: the *p*-xylylendiamide protons (H_H_), one of the protons of the isophthalamide motifs (H_D_) and the NH protons prove their closeness and, therefore, the structure of **1**. A deeper analysis of the NOESY experiment also allows to discriminate between the transoid and cisoid butyl groups of both amide functionalities respect to the carbonyl group.

After the spectroscopic analysis, the students report their reaction outcomes while describing what data allow them to come to their specific conclusions.

As a whole, all of the students accomplished the synthesis and structural characterization of a rotaxane and seemed to enjoy the process. The difficulties found by some students were overcome with the assistance of the instructor (e.g., misassigned signals in the NMR spectra or the use of specific technical language). After this laboratory experience, the students reinforced key concepts previously acquired, including those related to spectroscopic techniques. Besides, they could execute more complex laboratory operations, especially those in the context of the chemistry of MIMs, a completely new field for them. Overall, this practical experience stimulated them to acquire new knowledge about rotaxanes in a more proactive way than with the use of written sources.

### Modification of the Content for the Laboratory

After the experiment was carried out by the students, the background section was revised and some key concepts were incorporated (see SI Student handout modified): (i) a part dedicated to basic concepts of supramolecular chemistry; and (ii) a section focused on the template effect. These additions were made after verifying the lack of knowledge in this area and the relevance of this topic in the complete understanding of the experiment. Furthermore, the questions planned to be asked during the sessions were incorporated into the student material to strengthen fundamental learning. Thus, students be able to prepare them before starting the sessions.

The direct observation of the experimental work of the students allowed the instructors to improve the schedule and also to be aware of some specific experimental issues which were needed to be emphasized, such as the correct position of the syringes in the addition pump and the adequate compaction of the Celite pad.

### Evaluation of the Methodology

The laboratory experience described herein turned out to be enlightening for both students and instructors and allows us to develop experimental content in a proactive way. The experiment accomplished the expected goals, and students not only increased their interest and understanding about supramolecular chemistry and the mechanical bond but also reinforced their experimental skills, as well as the ability to analyze spectroscopic data. Furthermore, NMR techniques were introduced as robust techniques to identify interlocked structures. Additionally, students increased their confidence in addressing questions about unknown outcomes.

Regarding the instructors, a closer assessment of the learning outcomes as well as those concepts that become difficult to fully grasp by the students can be estimated before implementing this content into the academic curricula. Thus, further modifications can be included to easily reach the pedagogic goals. Below are the primary observations from the assessment of the learning objectives: (i) all students successfully synthesized and isolated the target rotaxane; (ii) almost all students found the NMR assignment of cisoid and transoid butyl groups of the threads difficult by using bidimensional NMR experiments; (iii) the yield of the rotaxane using the best tested conditions both employing syringe pumps and dropping funnels ranged from 62% to 71%; and (iv) all students found useful and interesting the experience according to the provided feedback, concluding that they learned key concepts, including the definition of mechanical bond and rotaxane, as well as analytical techniques to determine the interlocked nature of these compounds.

The dynamic role of the students in this proactive methodology is helpful for the performance of the instructors in the following terms: (i) direct observation of the experimental work which allows the identification of some experimental problems and the estimated duration times of the experiments; (ii) better perception of the difficulties that students may have with the structural elucidation; (iii) more productive feedback to the students during the performance of the experiments and seminars; and (iv) comments from the students about the strengths and weaknesses of the experience at the end of the laboratory practice.

## Experiment Customization

The current protocol can be easily customized from an experimental point of view. In this sense, a longer version has been designed that incorporates chromatography purification. Thus, the rotaxane could be purified by using silica gel as stationary phase and a 9/1 (v/v) chloroform/acetone mixture as the eluent, requiring an additional period of 2–3 h.

Even more, this experiment could be incorporated into the schedule of an experimental subject for upper-division undergraduate students. Hence, a document including an adaptation proposal is also provided (see SI Proposal). This new concept involves additional and more complete prelab and postlab activities, including a final research report. This document should comprise the following aspects: (i) presentation; (ii) coverage; (iii) analysis and evaluation of results; (iv) discussion and conclusions. Additionally, the spectroscopic analysis of the interlocked product **1** is, by itself, a useful learning tool for a seminar in the context of structural determination. For its implementation, we suggest providing the FAIR (Findable, Accessible, Interoperable, and Reusable) data of the NMR spectra to the students so that they can assign the signals using the MestreNova Software. If a license is not available, we suggest two free available options, iNMR and NMRium.

Although the current content has been designed for an optional internship, with a limited number of upper-division undergraduate students working individually, it is possible to increase the number of students by grouping them into pairs. Finally, if automatic addition pumps^37^ are not available, a two necked flask having attached two dropping funnels can be used, but this will lead to less reliable results since it will depend on the student precision. The use of dropping funnels will require an occasional monitoring of the addition rate (at least two–four times along the addition process), limiting the time available for additional explanations during the experiment. Furthermore, the flow rate cannot be finely modulated, leading to less accuracy in controlling the added volume and the different addition rates of both reactants. Cheaper homemade alternatives constructed with or without parts using a 3D printer can be considered.^38^

## Conclusions

The hands-on learning experience described herein is focused on the synthesis of a [2]rotaxane, having a tetrabutylfumaramide and a tetralactam macrocycle, by using two different methodologies: three- and five-component coupling reactions. Through different dilution conditions and addition times, the key role of the supramolecular complex in the formation of the target intertwined compound is studied based on the obtained yield. NMR spectroscopic data are employed to unequivocally confirm the interlocked nature of the product. This research activity introduces students to the chemistry of the mechanical bond, which sparks their curiosity and engages their interest. The dynamic role of the students has allowed us to make a more realistic estimation of the time required for the development of the practical lesson as well as those theoretical aspects which need more emphasis in order to successfully conduct the experiment.

## Supporting Information Available

Proposal (PDF, DOCX)
Instructor notes (PDF, DOCX)
NMR spectra (ZIP)
Student handout draft (PDF, DOCX)
Student handout modified (PDF, DOCX)
Seminar presentation (ZIP)

## Dedication

We dedicate this work to Professors Inmaculada Cartagena and Antonio Arques for their excellent teaching work at the Universidad de Murcia.
