# Supplementary material for: Exploring the Chemistry of the Mechanical Bond: Synthesis of a [2]Rotaxane through Multicomponent Reactions
Source: J Chem Educ. 2023 Aug 1;100(9):3355–63. doi: 10.1021/acs.jchemed.3c00163 (PMC10501439; doi:10.1021/acs.jchemed.3c00163)
Supplement: Supplementary file 2 — ed3c00163_si_002.docx [file ed3c00163_si_002.docx]

Exploring the Chemistry of the Mechanical Bond: Synthesis of a [2]Rotaxane through Multicomponent Reactions

Adrian Saura-Sanmartin,* Jorge Lopez-Sanchez, Carmen Lopez-Leonardo, Aurelia Pastor and Jose Berna*

Departamento de Química Orgánica, Facultad de Química, Regional Campus of International Excellence “Campus Mare Nostrum”, Universidad de Murcia, E-30100, Murcia, Spain. E-mail: adrian.saura@um.es, ppberna@um.es

**Supporting Information Proposal**

This document includes a proposal for adapting this experiment to the syllabus of an evaluable experimental subject. To this end, a modified student handout is provided, including a tentative standard grading rubric.

**1 Aims**

- To introduce students to the the usual methods for the synthesis of rotaxanes.

- To highlight the key role of the template effect in the synthesis of interlocked compounds.

- To synthesize a [2]rotaxane through a five or three-components clipping reaction.

- To reinforce key organic chemistry techniques and learn new ones.

- To consolidate previously acquired principles of FT-IR and NMR spectroscopy and apply them to the structural elucidation of an interlocked compound.

**2 Learning Outcomes**

At the end of these sessions, you should be able to:

- Extract the relevant information from the bibliography given by the instructor.

- Learn experimental techniques employed in laboratories of organic chemistry.

- Learn and develop skills regarding to the performance of a research project.

- Perform chemical reactions under high dilution conditions.

- Compare, discuss, and interpret a set of results obtained under different reaction conditions on the basis of the provided background.

- Check the purity of organic compounds by thin layer chromatography (TLC).

- Increase your knowledge of FT-IR and one-dimensional (^1^H, ^13^C, DEPT, APT and 1D-NOESY) and two-dimensional (^1^H-^1^H-COSY, ^1^H,^1^H-NOESY, ^1^H,^13^C-HSQC and ^1^H,^13^C-HMBC) NMR spectroscopic techniques and their application to the structural characterization of the synthesized rotaxane.

- Relate the knowledge acquired with that studied all along the Degree in Chemistry.

**3 Schedule**

This experience will be carried out in three 3-hour laboratory periods. You will organize yourselves in pairs, but you must submit your reports individually.

This laboratory experiment addresses important concepts of organic chemistry linked to amide chemistry, mechanical bonds, melting point, FT-IR and NMR techniques.

One week before the beginning of the sessions, the instructor will send an e-mail to the student with the “Student handout” containing bibliographic information and the pre-labs that you will have to bring with you for correction on the first day.

Day 1. Seminar.

Before carrying out the experiments, an introductory session is planned, including a power point presentation entitled “Mechanically Interlocked Molecules and the particular case of rotaxanes”. You will also carry out the structural assignment of the starting materials for which you will be provided with IR and NMR spectra, followed by the resolution of your pre-labs (sheets 1, 2 and 3). After this session you will have to do the post-lab 1 (sheet 4).

Day 2. Lab Session 1.

This class involves the preparation and isolation of a [2]rotaxane based on a fumaramide thread under the reaction conditions assigned by the instructor. You will perform an IR spectrum of the obtained product and prepare a sample for NMR analysis. Instructors can use free time during the sessions to teach/remind you of the spectroscopic techniques used in this experiment.

Day 3. Lab Session 2.

The results of all the experiments will be compared and the reaction conditions for which the highest yield was obtained will be determined. In addition, prior to this session you will receive the NMR spectra of your products and you will make a tentative assignment which will be discussed with the instructor. After this session you will have to do the lab 1 (sheet 5).

Finally, you must submit a written report which should contain the following sections: introduction, objectives, results and discussion and conclusions.

**4 Background^[[1]](#footnote-1)^**

Mechanically interlocked molecules (MIMs) are a type of compounds constituted by at least two submolecular components.^1,2^ These components are not covalently linked, but they are topologically interlocked one to each other. This particular link between the subcomponents of these molecules is known as the mechanical bond.^3^

In order to form a mechanical bond, it is necessary to arrange the different components in an orthogonal geometry. For this purpose, different methods for the assembly of MIMs have been developed over the years. The assembly employing transition metals as templates allows this union to be established by coordination of both components with a metal ion. Through the hydrophobic effect, several components can be joined by forming a hydrophobic cavity. The host-guest interactions between π-donors and acceptors allow the interconnection by the attraction of the components. Another particularly useful approach for obtaining MIMs is the establishment of hydrogen bond interactions between both components.^4,5,6,7,8^

Hydrogen bonds are characterized by their directionality and ability to act cooperatively, forming associations between donor and acceptor groups (Figure S1). In this type of bond, the donor group, the hydrogen atom, and the acceptor one (usually a heteroatom) are aligned. These bonds have a high dipolar character.^9,10^

***Figure S1.*** *Water molecules associated by hydrogen bonds. Reprinted with permission of the author from* [*http://hdl.handle.net/10201/104842*](http://hdl.handle.net/10201/104842)*.*

Within the wide range of synthetic MIMs, two families must be highlighted due to their versatility and their extraordinary properties: catenanes and rotaxanes (Figure S2). Catenanes are molecules in which at least two macrocycles are interlocked, mimicking the chain links.^11^ In the particular case of rotaxanes, the two components are of different type, one linear and one cyclic. The simplest example of a rotaxane could be considered as a dumbbell, a linear component with bulky groups at the ends, surrounded by a cyclic one. The bulky groups are known as stoppers and their purpose is to prevent the dethreading of the components.^12^ One would think that, to achieve the synthesis of a rotaxane, it is necessary to know how to sew molecules. The linear component, better known as axis or thread, should be introduced through the cyclic component or macrocycle.


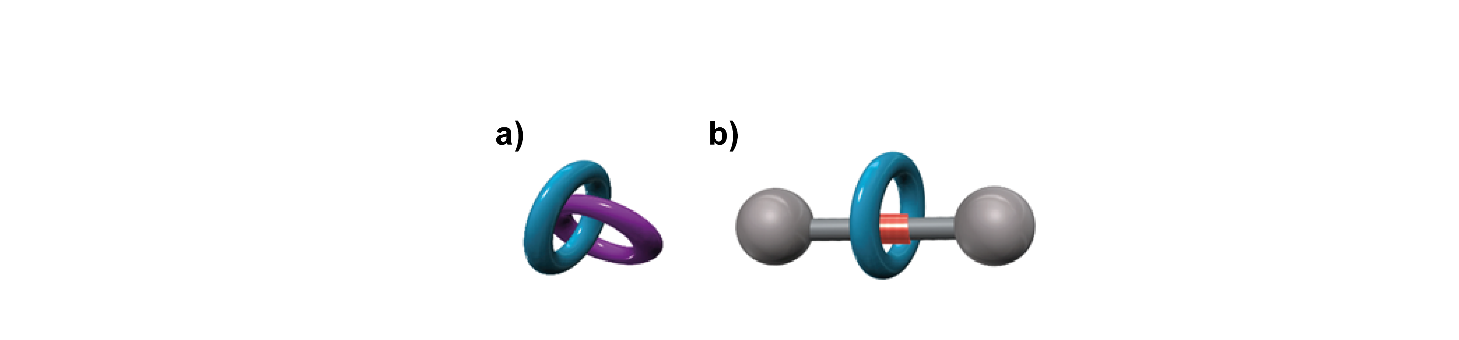


***Figure S2.*** *Cartoon representation of a: (a) catenane; and (b)rotaxane*. *Reprinted with permission of the author from* [*http://hdl.handle.net/10201/104842*](http://hdl.handle.net/10201/104842)*.*

The first rotaxane was synthesized by Harrison and Harrison more than five decades ago via a statistical approach (Figure S3a).^13^ This type of protocol is characterized by an absence of interactions between the precursors to allow their efficient orientation, affording the interlocked species in very low yields. Later, Professor Schill used a covalent-bond-directed synthesis, which involved the formation of a reversible covalent bond between linear and cyclic components, to obtain another rotaxane (Figure S3b). Although the product was obtained in a higher yield, the synthetic route involved a high number of reaction steps.^14^

 ***Figure S3.*** *Structure of (a) a [2]rotaxane prepared by a statistical synthesis ^13^ and (b) a [2]rotaxane prepared by a covalent bond-directed synthesis.^14^ Reprinted with permission of the author from* [*http://hdl.handle.net/10201/104842*](http://hdl.handle.net/10201/104842)*.*

Since the publication of these early examples, the synthesis of rotaxanes has undergone a remarkable development characterized by the variety of available methods and a significant increase in reaction yields. The most advantageous synthetic methods employ a template that appropriately orients the precursors in space. These methods involve the prior formation of a supramolecular complex stabilized by non-covalent interactions.^15^ Subsequent covalent modifications prevent the dissociation of these components.

Template-based methods for the obtention of rotaxanes can be classified into five main types (Figure S4). The capping methodology (Figure S4a), which involves the prior formation of a pseudorotaxane and its subsequent capping. In the snapping methodology (Figure S4b), once a semirotaxane has been formed, the capping of the non-stopper end is accomplished. In the slipping methodology (Figure S4c), the cyclic component is threaded onto a thread having stoppers under high temperature conditions. The clipping methodology (Figure S4d) involves the cyclisation of a ligand around a thread having stoppers. In the metal-active template method (Figure S4e), the metal plays a dual role, orienting the ligands in the appropriate geometry and catalyzing the formation of the covalent bond that captures the interlocked species.


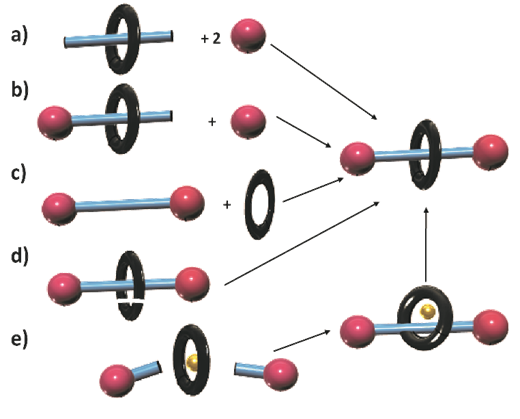


***Figure S4.*** *Main methods for the synthesis of [2]rotaxanes: (a) capping, (b) snapping, (c) slipping, (d) clipping and (e) active metal templating.*

The template syntheses are based on the preorganization of different molecules to form a supramolecular complex by introducing a guest molecule that can establish interactions with a host molecule. One well-known strategy using template synthesis is that based on metal ion chelates which act as templates. Thus, the synthesis of crown ether macrocycles is a really instructive example. In the Figure S5, first a supramolecular complex is form by wrapping the linear ligand around the potassium ion. A subsequent ring closing reaction leads to the target crown ether.^16^

***Figure S5.*** *Template synthesis of a crown ether.*

Rotaxanes are the most interesting type of MIMs because of their greater variety of motions and a large number of applications.^12^

In Nature, MIMs play a fundamental role in many processes occurring within the organism. The establishment of mechanical bonds is critical for the development of various biological processes, such as mitochondrial scission, selective ion transport or DNA replication, which proceed through mechanically interlocked intermediates.^1,16,17,18,19^ Nature is always ahead of Science. Whatever a scientist thinks of designing, Nature did it previously. This is why Nature is a great source of inspiration, in particular a wide number of biological systems. There is a duality that may seem contradictory. In order to understand Nature, it is necessary the progress in Science and, for the improvement in research, it is essential to interpret Nature. Thus, chemists have dedicated a lot of effort to understand the chemistry of the mechanical bond, thus overcoming the inherent difficulty in achieving the adequate spatial arrangement of the different components.


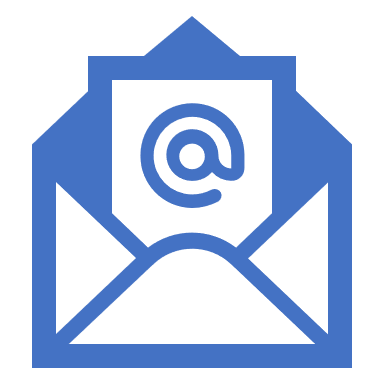
Here fill in the Pre-lab 1 (correction day 1 – Seminar) and send your answers recorded as "lastname_firstname_pre-lab-1" as a .pdf file, within 5 days at the latest.

**5 Safety and Hazards**

Chemistry laboratories are potentially dangerous places and therefore must be treated with respect. The following rules are for the safety of you, other students and members of staff and should be followed at all times.

- You should wear appropriate personal protective equipment, such as disposable gloves, goggles, closed shoes and a lab coat.

- The procedures must be performed in a fume hood or similarly ventilated workspace.

- Make sure that all glassware containing chemicals is properly labelled.

- Liquid and solid waste must be disposed into sealed and appropriately labeled containers.

- The rinsed syringe should be disposed into a dedicated, appropriately labelled disposal container. The rinsed needle can be reused after being dried in an oven.

- Report all accidents or near accidents to the lecturer in charge of the class.

- Do not remove samples or chemicals from the laboratory.

Safety information for all reagents is available via the appropriate Safety Data Sheet (SDS).


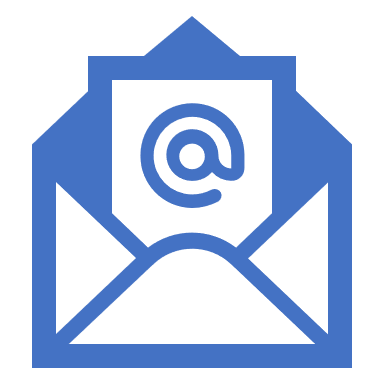
Here fill in the Pre-lab 2 (correction day 1 – Seminar) and send your answers recorded as "lastname_firstname_pre-lab-2" as a .pdf file, within 5 days at the latest.

**6 General Experimental Information**

All commercially available compounds were purchased from Merck, Acros Organics, or Alfa-Aesar Chemical Co. and used without purification. *N*^1^,*N*^1^,*N*^4^,*N*^4^-Tetrabutylfumaramide (**2**) was prepared from fumaroyl dichloride and dibutylamine ^20^ and the pre-formed U-shaped component. *N*^1^,*N*^3^-Bis[4-(aminomethyl)benzyl]isophthalamide (**5**), was generated in situ by the acidic deprotection of the corresponding di-Boc derivative, ^21^ following the described procedures. HPLC grade solvents (Scharlab) were nitrogen-saturated as well dried and deoxygenated using an Innovative Technology Inc. Pure-Solv 400 Solvent Purification System. Deionized water is used in the preparation of all aqueous solutions. Brine refers to a saturated aqueous solution of sodium chloride.

**TLC** chromatographies are performed on precoated silica gel on aluminum cards (0.25 mm thick, with 254 nm fluorescent indicator, Fluka) and observed under UV light (254 or 365 nm).

The **additions** are made with *kd* Scientific motor-driven syringe pumps (model number 101) and stainless steel 304 syringe needle, noncoring point (30 cm) from Sigma-Aldrich.

**Under reduced pressure** refers to the use of a Büchi Rotavapor R-3000 or a Heidolph Hei-Vap Value G3 apparatus with a Vacuumbrand CVC 3000 vacuum pump equipped with a water bath for aiding to remove the solvent.

**Melting point (m.p.)** is determined on a Kofler hot-plate melting point apparatus and is uncorrected.

**Infrared spectroscopy** is performed using a PerkinElmer Spectrum 65, FT-IR Spectrometer (ATR) in the range 4000-600 cm^-1^. The intensities of the absorption bands are indicated as vs (very strong), s (strong), m (middle) and w (weak).

**NMR** spectra are recorded at 298 K using a Bruker Avance 400 instrument (400 MHz ^1^H frequency, 101 MHz ^13^C frequency). Chemical shifts are quoted in parts per million (ppm), referenced to residual chloroform (7.26 ppm for ^1^H NMR, 77.00 ppm for ^13^C NMR), as internal standard, whereas coupling constants, *J*, are quoted in Hz. Multiplicities are quoted as s (singlet), d (doublet), dd (doublet of doublet), t (triplet) and m (multiplet). Signals in the ^1^H and ^13^C NMR spectra of the synthesized compounds are assigned with the aid of DEPT, APT, or two-dimensional NMR experiments (COSY, HMQC, HMBC and NOESY). All NMR data are processed using the MestReNova software (from MestreLab Research S. L.).

**7 Experimental Procedure for the five-component synthesis of [2]rotaxane 1**^22^

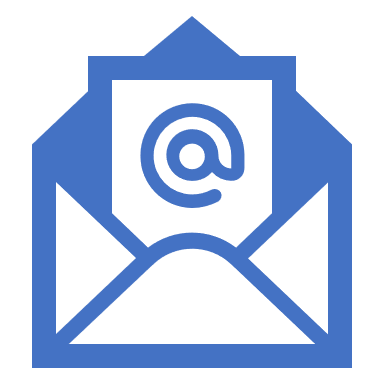
Here fill in the Pre-lab 3 (correction day 1 – Seminar) and send your answers recorded as "lastname_firstname_pre-lab-3" as a .pdf file, within 5 days at the latest.

**Protocol**:

The amounts of each reagent and solvent used, as well as the addition time for each pair of students are specified in Table S1.

1. CHCl_3_ (n - 40 mL) is added to a round bottom flask (RBF) equipped with a stir bar.
2. The thread **2** (x equiv) and Et_3_N (z/2 equiv) were added to the RBF.
3. The RBF is sealed with a septum and the solution is stirred vigorously.
4. Two 25 mL syringes are prepared with the needles previously dried in the oven or alternatively, two dried dropping funnels.
5. The first syringe or dropping funnel is filled with a solution of isophthaloyl dichloride (**3**) (y equiv) in CHCl_3_ (20 mL).
6. A solution of *p*-xylylenediamine (**4**) (y equiv) and Et_3_N (z/2 equiv) in CHCl_3_ (20 mL) is loaded into a second syringe or dropping funnel.
7. The two syringes are adapted to the motor-driven syringe pump and programmed for the assigned addition time according to table S1. When dropping funnels are used instead of syringes, the addition rate should be controlled manually.
8. After completion of the addition, the reaction mixture is stirred for additional 10 minutes.
9. The resulting suspension is filtered through a Celite pad.
10. The filtrate is sequentially washed with water (2 x 50 mL), a solution of HCl 1 M (2 × 50 mL), a saturated solution of NaOH 1 M (2 × 50 mL) and brine (2 × 50 mL).
11. The organic phase was then dried with anhydrous MgSO_4_.
12. The solution is filtered and the solvent was removed under reduced pressure.
13. The resulting solid is filtered and washed with diethyl ether (3 x 15 mL) until all the unreacted thread **2** is extracted. To identify the product on TLC it is a common practice to run a side-by-side comparison of all material potentially present in the crude mixture. Make a TLC (SiO_2_, CHCl_3_/acetone 9:1) after each extraction with 5 mL of ether, comparing with a pure sample of fumaramide **2**.
14. The solid [2]rotaxane **1** is dried under vacuum.

**Product identification:**

1. Measure the melting point of your product and compare it with that described in the literature.
2. Performs an IR-ATR spectrum of your product.
3. Prepare a sample for NMR analysis with 15 mg of your product and CDCl_3_.
4. With the help of your instructor, analyze the following one-dimensional spectra: ^1^H, ^13^C and DEPT-135 (or APT) and two-dimensional spectra: ^1^H,^1^H-COSY, ^1^H,^1^H-NOESY, HSQC and HMBC.
5. Process your NMR data using the MestReNova software.


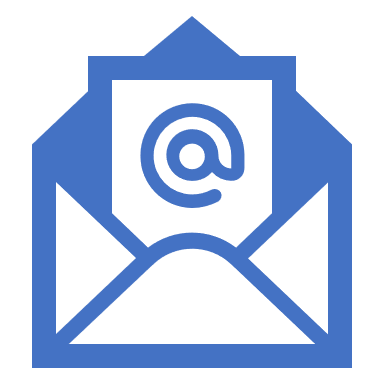
Here fill in the Lab 1 (to be completed in Session 2) and send your answers recorded as "lastname_firstname_lab-1" as a .pdf file, within 5 days at the latest.

**8 Experimental Procedure for the three-component synthesis of [2]rotaxane 1**

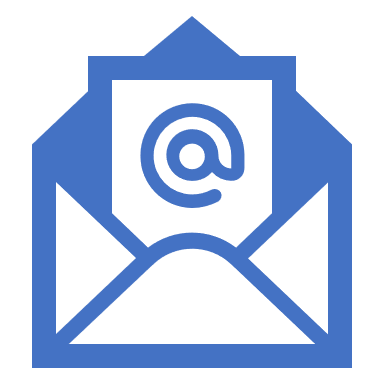
Here fill in the Pre-lab 3 (correction day 1 – Seminar) and send your answers recorded as "lastname_firstname_pre-lab-3" as a .pdf file, within 5 days at the latest.

**Protocol**:

The amounts of each reagent and solvent used, as well as the addition time for each pair of students are specified in Table S1.

1. CHCl_3_ (n - 20 mL) is added to a round bottom flask (RBF) equipped with a stir bar.
2. The thread **2** (x equiv), the pre-formed U-shaped **5** (y equiv) and Et_3_N (z equiv) were added to the RBF.
3. The RBF is sealed with a septum and the solution is stirred vigorously.
4. One 25 mL syringe is prepared with the needle previously dried in the oven.
5. The syringe is filled with a solution of isophthaloyl dichloride (**3**) (y equiv) in CHCl_3_ (20 mL).
6. The syringe is adapted to the motor-driven syringe pump and programmed for the assigned addition time according to table S1.
7. After completion of the addition, the reaction mixture is stirred for additional 10 minutes.
8. The resulting suspension is filtered through a Celite pad.
9. The filtrate is sequentially washed with water (2 x 50 mL), a solution of HCl 1 M (2 × 50 mL), a saturated solution of NaOH 1 M (2 × 50 mL) and brine (2 × 50 mL).
10. The organic phase was then dried with anhydrous MgSO_4_.
11. The solution is filtered and the solvent was removed under reduced pressure.
12. The resulting solid is filtered and washed with diethyl ether (3 x 15 mL) until all the unreacted thread **2** is extracted. To identify the product on TLC it is a common practice to run a side-by-side comparison of all material potentially present in the crude mixture. Make a TLC (SiO_2_, CHCl_3_/acetone 9:1) after each extraction with 5 mL of ether, comparing with a pure sample of fumaramide **2**.
13. The solid [2]rotaxane **1** is dried under vacuum.

**Product identification:**

1. Measure the melting point of your product and compare it with that described in the literature.
2. Performs an IR-ATR spectrum of your product.
3. Prepare a sample for NMR analysis with 15 mg of your product and CDCl_3_.
4. With the help of your instructor, perform the following one-dimensional spectra: ^1^H, ^13^C and DEPT-135 (or APT) and two-dimensional spectra: ^1^H,^1^H-COSY, ^1^H,^1^H-NOESY, HSQC and HMBC.
5. Process your NMR data using the MestReNova software.


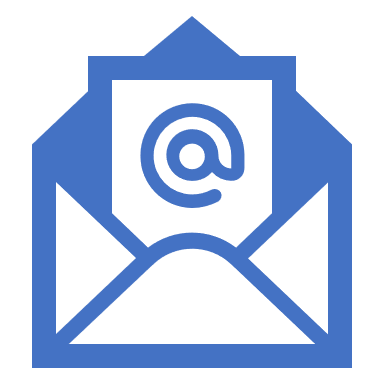
Here fill in the Lab 1 (to be completed in Session 2) and send your answers recorded as "lastname_firstname_lab-1" as a .pdf file, within 5 days at the latest.

Each pair of students will carry out the synthesis of the [2]rotaxane **4** according to the distribution in Table S1.

**Table S1**. Reaction conditions assigned to each pair of students

| Group | Five-Component  Reaction^a^ | Three-Component  Reaction^b^ | Solvent  (n mL) | Addition Time  (min) |
| --- | --- | --- | --- | --- |
| 1, 2 | Conditions A |  | 90 | 30 |
| 3, 4 | Conditions A |  | 90 | 60 |
| 5, 6 | Conditions A |  | 90 | 90 |
| 7, 8 | Conditions A |  | 180 | 30 |
| 9, 10 | Conditions A |  | 180 | 60 |
| 11, 12 | Conditions A |  | 180 | 90 |
| 13, 14 | Conditions A |  | 360 | 90 |
| 15, 16 | Conditions B |  | 90 | 90 |
| 17, 18 | Conditions B |  | 180 | 90 |
| 19, 20 |  | Conditions A | 180 | 90 |
| 21, 22 |  | Conditions B | 180 | 90 |

^a^ Conditions A: **2** (1 equiv), **3** (8 equiv), **4** (8 equiv), Et_3_N (24 equiv). Conditions B: **2** (8 equiv), **3** (1 equiv), **4** (1 equiv), Et_3_N (8 equiv).

^b^ Conditions A: **2** (1 equiv), **5** (8 equiv), **3** (8 equiv), Et_3_N (24 equiv). Conditions B: **2** (8 equiv), **5** (1 equiv), **3** (1 equiv), Et_3_N (8 equiv).

**9 Pre and post-lab sheets**

Pre-lab 1 sheet 1 (correction day 1 – Seminar). (4 pt/100 pt)

1. Define the following concepts:

(a) supramolecular chemistry

(b) supramolecular complex

(c) chemical bond

(d) hydrogen bond

(e) mechanical bond

2. Search on the internet for information about the 2016 Nobel Prize in Chemistry and answer the following questions:

(a) What was the topic of the prize?

(b) What is a molecular machine?

(c) What is a rotaxane?

3. Draw the structure of:

(a) a fumaramide

(b) a tetralactam macrocycle

4. Which bibliographical reference is closely related to the work we are going to develop in the laboratory?

Pre-lab 1 sheet 2 (correction day 1 – Seminar). (4 pt/100 pt)

CAS Registry Numbers (often referred to as CAS RN^®^ or CAS Numbers) are universally used to provide a unique, unmistakable identifier for chemical substances.

The Globally Harmonised System (GHS) is a single worldwide system for classifying and communicating the hazardous properties of industrial and consumer chemicals.

Some CAS Numbers and GSH Hazards of the substances you are going to use are listed below. Complete the tables S2 and S3 by filling in the blanks.

**Table S2.** Hazards of the chemical compounds employed in the laboratory experiment.

| **Chemical Compound** | **CAS Number** | **GHS Hazards** |
| --- | --- | --- |
| Celite | 68855-54-9 | H372: Causes damage to lungs through prolonged or repeated exposure if inhaled. |
| Hydrochloric acid | 7647-01-0 | H314: Causes severe skin burns and eye damage.  H331: Toxic if inhaled. |
| Isophthaloyl dichloride | ­__________ | H312: __________________________  H314: Causes severe skin burns and eye damage.  H318: Causes serious eye damage.  H331: Toxic if inhaled. |
| Magnesium sulphate anhydrous | 7487-88-9 | _______________________________ |
| Sodium chloride | ____________ | H319: Causes serious eye irritation. |
| Sodium hydroxide | 1310-73-2 | ______________________________ |
| Triethylamine | ____________ | H225: _________________________  H302: _________________________  H312: _________________________  H314: Causes severe skin burns and eye damage.  H332: _________________________ |
| *p*-Xylylenediamine | 539-48-0 | ______________________________ |

**Table S3.** Hazards of the solvents employed in the laboratory experiment.

| **Solvent** | **CAS Number** | **GHS Hazards** |
| --- | --- | --- |
| Acetone | ____________ | H225:  H319: Causes serious eye irritation.  H336: May cause drowsiness or dizziness. |
| Chloroform | 67-66-3 | ______________________________ |
| Chloroform-*d* | ____________ | H302: ­­_________________________  H315: _________________________  H319: Causes serious eye irritation.  H331: Toxic if inhaled.  H336: May cause drowsiness or dizziness.  H351: _________________________  H361: _________________________  H372: Causes damage to lungs through prolonged or repeated exposure if inhaled.  H373: _________________________ |
| Diethyl ether | 60-29-7 | ______________________________ |

Pre-lab 3 sheet 3 (correction day 1 – Seminar). (6 pt/100 pt)

1. it is necessary to prepare 100 mL of HCl 1 M from the commercial one. Calculate the amount (mL) you need of the commercial one (37%) and explain how you would prepare it.

2. Calculate the amount of NaOH you need to weigh in order to obtain 100 mL of a 1 M solution. Explain how you would prepare it.

3. Complete the following tables (S4 and S5) with appropriate values for the reagents to be used. Some information has been provided to guide you.

**Table S4**. Data for the five-component synthesis of [2]rotaxane **1**

|  | **2** | **3** | **4** | **Et_3_N** |
| --- | --- | --- | --- | --- |
| MF | C_20_H_38_N_2_O_2_ | C_8_H_4_Cl_2_O_2_ | C_8_H_12_N_2_ | C_6_H_15_N |
| MW | 338.54 | 203.02 | 136.20 | 101.19 |
|  | Conditions A | | | |
| equiv | 1 | 8 | 8 | 24 |
| mmol | ___ | ___ | ___ | ___ |
| g or mL | 0.2 g | ___ g | ___ g | ___ g (___ mL) |
|  | Condition B | | | |
| equiv | 8 | 1 | 1 | 8 |
| mmol | ___ | ___ | ___ | ___ |
| g or mL | 0.6 g | ___ g | ___ g | ___ g (___ mL) |

**Table S5**. Data for the three-component synthesis of [2]rotaxane **1**

|  | **2** | **5** | **3** | **Et_3_N** |
| --- | --- | --- | --- | --- |
| MF | C_20_H_38_N_2_O_2_ | C_24_H_26_N_4_O_2_ | C_8_H_4_Cl_2_O_2_ | C_6_H_15_N |
| MW | 338,54 | 402.49 | 203.02 | 101,19 |
|  | Conditions A | | | |
| equiv | 1 | 8 | 8 | 24 |
| mmol | ___ | ___ | ___ | ___ |
| g or mL | 0.2 g | ___ g | ___ g | ___ g (___ mL) |
|  |  |  |  |  |
|  | Conditions B | | | |
| equiv | 8 | 1 | 1 | 8 |
| mmol | ___ | ___ | ___ | ___ |
| g or mL | 0.6 g | ___ g | ___ g | ___ g (___ mL) |

Post lab 1 sheet 4 (6 pt/100 pt)


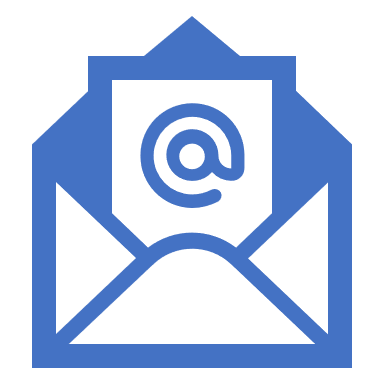
Here fill in the Post lab 1 (concerning the assignment done in the Seminar under the supervision of the instructor) and send your answers recorded as "lastname_firstname_post-lab-1" as a .pdf file, within 5 days at the latest.

1. Draw the structure of:

(a) *N*^1^,*N*^1^,*N*^4^,*N*^4^-tetrabutylfumaramide (**2**)

(b) *N*^1^,*N*^3^-bis[4-(aminomethyl)benzyl]isophthalamide (**5**)

2. Assign the most representative bands from the IR spectra of the above compounds.

3. Make a tentative assignment of the ^1^H NMR spectra of the above compounds.

Lab 1 sheet 5 (20/100 pt)

1. Determine which spot corresponds to your product based on comparison with the starting material.
2. Calculate the R_f_ value of your product.
3. Determine the most significant bands in the IR-ATR spectrum of the obtained rotaxane. Compare the bands due to the CO groups in the free fumaramide and when this is the thread of the rotaxane.
4. Interpret the ^1^H NMR spectrum by creating a table that lists the chemical shift, integral, and coupling constant (if applicable) for each signal.
5. Are the 4 butyl substituents equivalent?
6. Determine with the help of ^1^H,^1^H-NOESY which is the butyl group *transoid* to the CO of the fumaramide subcomponent.
7. Assign all proton signals with the help of the ^1^H,^1^H-COSY and ^1^H,^1^H-NOESY experiments.
8. List the signals observed in the ^13^C NMR spectrum.
9. Assign all carbon signals with the help of HMQC and HMBC.
10. Which are the interactions between the two subcomponents (fumaramide and macrocycle units) observed in the ^1^H,^1^H-NOESY experiment?

**10 Standard grading rubrics**

Session 1 (20 pt/100 pt).

Pre-lab sheet 1 (4 pt/100 pt)

Pre-lab sheet 2 (4 pt/100 pt)

Pre-lab sheet 3 (6 pt/100 pt)

Post-lab sheet 4 (6 pt/100 pt)

Session 2 (20 pt/100 pt).

Safety (4 pt/100 pt).

Execution of the experimental procedure (4 pt/100 pt).

Observations (2 pt/100 pt).

Theoretical yield calculation (2 pt/100 pt).

% Yield calculation (2 pt/100 pt).

Successful Product synthesis (2 pt/100 pt).

Performance of the IR spectrum (2 pt/100 pt).

Sample preparation for NMR analysis (2 pt/100 pt).

Session 3 (20 pt/100 pt).

Lab 1 sheet 5 (20 pt/100 pt)

Report (40 pt/100 pt)

The written component of the work constitutes 40% of the marks available for the project. The following sections will be assessed by ticking the box corresponding to each assessment criterion.

a) **Presentation –** Technical presentation and layout of report; clarity of language, figures, tables etc. and completeness of referencing.

| Poor (0-4.9 pt) | Acceptable (5-6.9 pt) | Good (7-8.9 pt) | Excellent (9-10 pt) |
| --- | --- | --- | --- |
|  |  |  |  |

b) **Coverage –** Coverage and comprehension of relevant source materials

| Poor (0-4.9 pt) | Acceptable (5-6.9 pt) | Good (7-8.9 pt) | Excellent (9-10 pt) |
| --- | --- | --- | --- |
|  |  |  |  |

c) **Analysis and evaluation of results** – Quality of analysis and evaluation of results

| Poor (0-4.9 pt) | Acceptable (5-6.9 pt) | Good (7-8.9 pt) | Excellent (9-10 pt) |
| --- | --- | --- | --- |
|  |  |  |  |

d) **Discussion and conclusions** – Quality of discussion and conclusion

| Poor (0-4.9 pt) | Acceptable (5-6.9 pt) | Good (7-8.9 pt) | Excellent (9-10 pt) |
| --- | --- | --- | --- |
|  |  |  |  |

**11 References**

1 C. J. Bruns, J. F. Stoddart. The Mechanical Bond: A Work of Art. *Top. Curr. Chem.* **2011**, *323*, 19-72.

2. J. E. M. Lewis, M. Galli, S. M. Goldup. Properties and Emerging Applications of Mechanically Interlocked Ligands. *Chem. Commun.* **2017**, *53*, 298-312.

3. E. A. Neal, S. M. Goldup. Chemical Consequences of Mechanical Bonding in Catenanes and Rotaxanes: Isomerism, Modification, Catalysis and Molecular Machines for Synthesis. *Chem. Commun.* **2014**, *50*, 5128-5142.

4. S. Erbas-Cakmak, D. A. Leigh, C. T. McTernan, A. L. Nussbaumer. Artificial Molecular Machines. *Chem. Rev.* **2015**, *115*, 10081-10206.

5. D. B. Amabilino, J. F. Stoddart. Interlocked and Intertwined Structures and Superstructures. *Chem. Rev.* **1995**, *95*, 2725-2828.

6. F. Vögtle, T. Dünnwald, T. Schmidt. Catenanes and Rotaxanes of the Amide Type. *Acc. Chem. Res.* **1996**, *29*, 451-460.

7. J. E. Beves, B. A. Blight, C. J. Campbell, D. A. Leigh, R. T. McBurney. Strategies and Tactics for the Metal-Directed Synthesis of Rotaxanes, Knots, Catenanes and Higher Order Links. *Angew. Chem., Int. Ed.* **2011**, *50*, 9620-9327.

8. N. H. Evans. Recent Advances in the Synthesis and Application of Hydrogen Bond Templated Rotaxanes and Catenanes. *Eur. J. Org. Chem.* **2019**, 3320-3343.

9. L. Pauling. The Structure and Entropy of Ice and of Other Crystals with Some Randomness of Atomic Arrangement. *J. Am. Chem. Soc.* **1935**, *57*, 2680-2684.

10. P. A. Kollman, L. C. Allen. The Theory of the Hydrogen Bond. *Chem. Rev.* **1972**, *72*, 283-303.

11. J. F. Stoddart. The Chemistry of the Mechanical Bond. *Chem. Soc. Rev.* **2009***, 38*, 1802-1820.

12. M. Xue, Y. Yang, X. Chi, X. Yan, F. Huang. Development of Pseudorotaxanes and Rotaxanes: From Synthesis to Stimuli-Responsive Motions to Applications. *Chem. Rev.* **2015**, *115*, 7398-7501.

13. I. T. Harrison, S. Harrison. Synthesis of a stable complex of a macrocycle and a threaded chain. *J. Am. Chem. Soc.* **1967**, *89*, 5723-5724.

14. G. Schill, H. Zollenkopf, Rotaxan‐Verbindungen, I. *Justus Liebigs Ann. Chem.* **1969**, *721*, 53-74.

15. F. G. Gatti, D. A. Leigh, S. A. Nepogodiev, A. M. Z. Slawin, S. J. Teat, J. K. Y. Wong. Stiff and Sticky in the Right Places: The Dramatic Influence of Preorganizing Guest Binding Sites on the Hydrogen Bond-Directed Assembly of Rotaxanes. *J. Amer. Chem. Soc.* **2001**, *123*, 5983-5989.

16. J. A. Mears, L. L. Lackner, S. Fang, E. Ingerman, J. Numari, J. E. Hinshaw. Conformational Changes in Dnm1 Support a Contactile Mechanism for Mitocondrial Fission. *Nat. Struct. Mol. Biol.* **2011**, *18*, 20-26.

17. E. Gouaux, R. MacKinnon. Principles of Selective Ion Transport in Channels and Pumps. *Science* **2005**, *310*, 1461-1465.

18. A. Tonath. Hibernating Bears, Antibiotics and the Evolving Ribosome. *Angew. Chem., Int. Ed.* **2010**, *49*, 4340-4354.

19. K. N. Kreuzer, N. R. Cozzarelli. Formation and Resolution of DNA Catenanes by DNA Gyrase. *Cell* **1980**, *20*, 245-254.

20. C. Lopez-Leonardo, A. Martinez-Cuezva, D. Bautista, M. Alajarin, J. Berna. Homo and heteroassembly of amide-based [2]rotaxanes using α,α'-dimethyl-*p*-xylylenediamines. *Chem. Commun.* **2019**, *55*, 6787-6790.

21. D. Gonzalez Cabrera, B. D. Koivisto, D. A. Leigh. A metal-complex-tolerant CuAAC ‘click’ protocol exemplified through the preparation of homo- and mixed-metal-coordinated [2]rotaxanes. *Chem. Commun.* **2007**, 4218-4220.

22. The conditions were modified based on the previously reported work: A. Martinez-Cuezva, F. Morales, G. R. Marley, A. Lopez-Lopez, J. C. Martinez-Costa, D. Bautista, M. Alajarin, J. Berna. Thermally and Photochemically Induced Dethreading of Fumaramide-Based Kinetically Stable Pseudo[2]rotaxanes. *Eur. J. Org. Chem.* **2019**, 3480-3488.

1. This text has been rewritten based on the introduction of the Doctoral Thesis entitled ‘Benzylic amide rotaxanes: study of the switching of the motion of their components and their organization in metal-organic frameworks’ (Spanish title: ‘Rotaxanos de amidas bencílicas: studio de la conmutación del movimiento de sus componentes y su organización en sistemas metal-orgánicos’) defended by Adrian Saura-Sanmartin on February 2021 at the Universidad de Murcia (<http://hdl.handle.net/10201/104842>). The author of the Thesis, a coauthor of this article, has consented to its use. [↑](#footnote-ref-1)
