# Supplementary material for: Exploring the Chemistry of the Mechanical Bond: Synthesis of a [2]Rotaxane through Multicomponent Reactions
Source: J Chem Educ. 2023 Aug 1;100(9):3355–63. doi: 10.1021/acs.jchemed.3c00163 (PMC10501439; doi:10.1021/acs.jchemed.3c00163)

Exploring the Chemistry of the Mechanical Bond: Synthesis of a [2]Rotaxane through Multicomponent Reactions

Adrian Saura-Sanmartin,* Jorge Lopez-Sanchez, Carmen Lopez-Leonardo, Aurelia Pastor and Jose Berna*

Departamento de Química Orgánica, Facultad de Química, Regional Campus of International Excellence "Campus Mare Nostrum", Universidad de Murcia, E-30100, Murcia, Spain. E-mail: adrian.saura@um.es, ppberna@um.es

Supporting Information Instructor Notes

1 Introduction

The following notes will help instructors to guide students through the organic synthesis experiments and the chemical analyses of the reaction product and by-products and to troubleshoot the project.

2 Purpose

This laboratory experiment pretends: (i) to introduce the undergraduate student into the world of supramolecular chemistry, focusing on mechanically interlocked molecules, with remarkable interest in rotaxanes; and (ii) to synthesize a [2]rotaxane through a multicomponent reaction and its characterization by IR and NMR spectroscopy.

3 Background[i]

[i] Part of this text has been rewritten based on the introduction of the Doctoral Thesis entitled 'Benzylic amide rotaxanes: study of the switching of the motion of their components and their organization in metal-organic frameworks' (Spanish title: 'Rotaxanos de amidas bencílicas: studio de la conmutación del movimiento de sus componentes y su organización en sistemas metal-orgánicos') defended by Adrian Saura-Sanmartin on February 2021 at the Universidad de Murcia (http://hdl.handle.net/10201/104842). The author of the Thesis, a coauthor of this article, has consented to its use.

Mechanically interlocked molecules (MIMs) are a type of compounds constituted by at least two submolecular components.[1,2] These components are not covalently linked, but they are topologically interlocked one to each other. This particular link between the subcomponents of these molecules is known as the mechanical bond.[3]

In order to form a mechanical bond, it is necessary to arrange the different components in an orthogonal geometry. For this purpose, different methods for the assembly of MIMs have been developed over the years. The assembly employing transition metals as templates allows this union to be established by coordination of both components with a metal ion. Through the hydrophobic effect, several components can be joined by forming a hydrophobic cavity. The host-guest interactions between π-donors and acceptors allow the interconnection by the attraction of the components. Another particularly useful approach for obtaining MIMs is the establishment of hydrogen bond interactions between both components.[4,5,6,7,8]

Hydrogen bonds are characterized by their directionality and ability to act cooperatively, forming associations between donor and acceptor groups (Figure S1). In this type of bond, the donor group, the hydrogen atom, and the acceptor one (usually a heteroatom) are aligned. These bonds have a high dipolar character.[9,10]

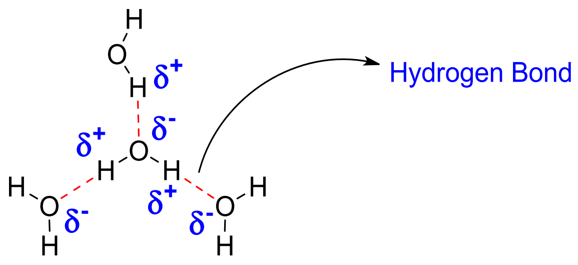

Figure S1. *Water molecules associated by hydrogen bonds. Reprinted with permission of the author from http://hdl.handle.net/10201/104842.*

Within the wide range of synthetic MIMs, two families must be highlighted due to their versatility and their extraordinary properties: catenanes and rotaxanes (Figure S2). Catenanes are molecules in which at least two macrocycles are interlocked, mimicking the chain links.[11] In the case of rotaxanes, the two components are of different type, one linear and one cyclic. The simplest example of a rotaxane could be considered as a dumbbell, a linear component with bulky groups at the ends, surrounded by a cyclic one. The bulky groups are known as stoppers and their purpose is to prevent the dethreading of the components.[12] One would think that, to achieve the synthesis of a rotaxane, it is necessary to know how to sew molecules. The linear component, better known as axis or thread, should be introduced through the cyclic component or macrocycle.

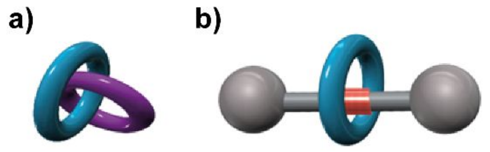

Figure S2. *Cartoon representation of a: (a) catenane; and (b)rotaxane. Reprinted with permission of the author from http://hdl.handle.net/10201/104842.*

The first rotaxane was synthesized by Harrison and Harrison more than five decades ago via a statistical approach (Figure S3a).[13] This type of protocol is characterized by an absence of interactions between the precursors to allow their efficient orientation, affording the interlocked species in very low yields. Later, Professor Schill used a covalent-bond-directed synthesis, which involved the formation of a reversible covalent bond between linear and cyclic components, to obtain another rotaxane (Figure S3b). Although the product was obtained in a higher yield, the synthetic route involved a high number of reaction steps.[14]

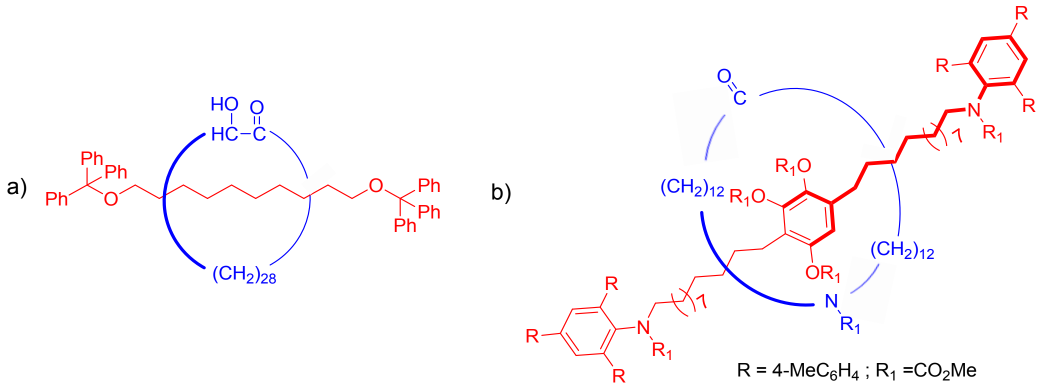

Figure S3. *Structure of (a) a [2]rotaxane prepared by a statistical synthesis* [13] *and (b) a [2]rotaxane prepared by a covalent bond-directed synthesis.*[14] *Reprinted with permission of the author from http://hdl.handle.net/10201/104842.*

Since the publication of these early examples, the synthesis of rotaxanes has undergone a remarkable development characterized by the variety of available methods and a significant increase in reaction yields. The most advantageous synthetic methods employ a template that appropriately orients the precursors in space. These methods involve the prior formation of a supramolecular complex stabilized by non-covalent interactions.[15] Subsequent covalent modifications prevent the dissociation of these components.

Template-based methods for the obtention of rotaxanes can be classified into five main types (Figure S4). The capping methodology (Figure S4a), which involves the prior formation of a pseudorotaxane and its subsequent capping. In the snapping methodology (Figure S4b), once a semirotaxane has been formed, the capping of the non-stopper end is accomplished. In the slipping methodology (Figure S4c), the cyclic component is threaded onto a thread having stoppers under high temperature conditions. The clipping methodology (Figure S4d) involves the cyclisation of a ligand around a thread having stoppers. In the metal-active template method (Figure S4e), the metal plays a dual role, orienting the ligands in the appropriate geometry and catalyzing the formation of the covalent bond that captures the interlocked species.

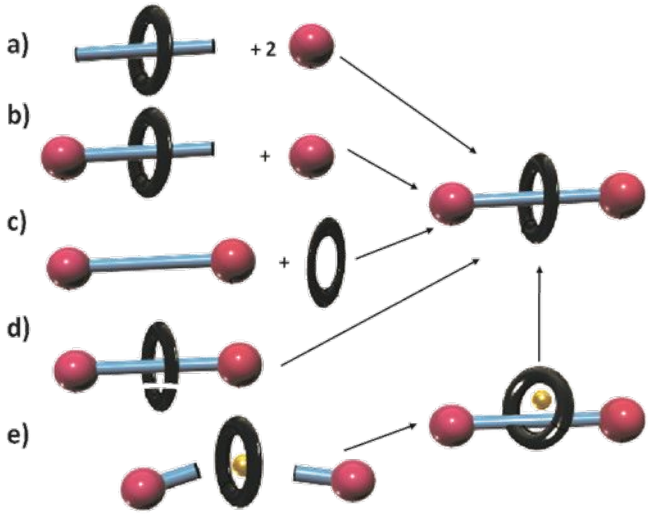

Figure S4. *Main methods for the synthesis of [2]rotaxanes: (a) capping, (b) snapping, (c) slipping, (d) clipping and (e) active metal templating.*

The template syntheses are based on the preorganization of different molecules to form a supramolecular complex by introducing a guest molecule that can establish interactions with a host molecule. One well-known strategy using template synthesis is that based on metal ion chelates which act as templates. Thus, the synthesis of crown ether macrocycles is a really instructive example. In the Figure S5, first a supramolecular complex is form by wrapping the linear ligand around the potassium ion. A subsequent ring closing reaction leads to the target crown ether.[16]

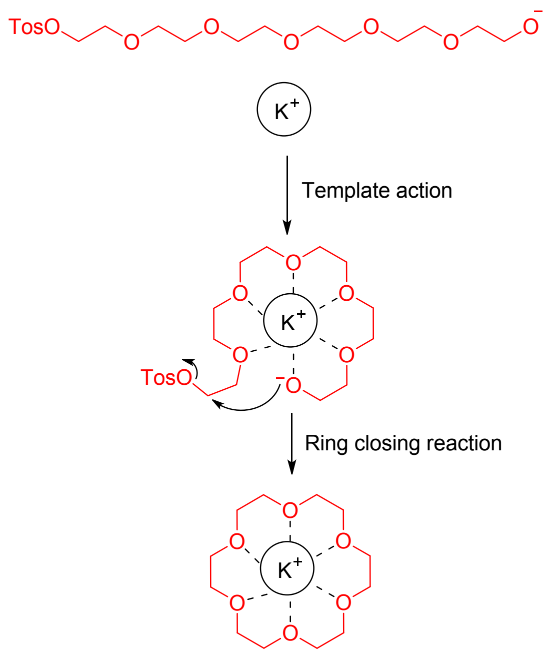

Figure S5. *Template synthesis of a crown ether.*

Rotaxanes are the most interesting type of MIMs because of their greater variety of motions and a large number of applications.[13]

In Nature, MIMs play a fundamental role in many processes occurring within organisms. The establishment of mechanical bonds is critical for the development of various biological processes, such as mitochondrial scission, selective ion transport or DNA replication, which proceed through mechanically interlocked intermediates.[1,17,18,19,20] Nature is always ahead of science. Whatever a scientist thinks of designing, Nature did it previously. Therefore, Nature is a great source of inspiration, in particular a wide number of biological systems. There is a duality that may seem contradictory. In order to understand Nature, it is necessary the progress in science and, for the improvement in research, it is essential to interpret Nature. Thus, chemists have dedicated a lot of effort to understand the chemistry of the mechanical bond, thus overcoming the inherent difficulty in achieving the adequate spatial arrangement of the different components.

4 Organization

This laboratory experience was designed to be carried out in three 3 h laboratory periods. This experiment is a part of an optional advance organic chemistry internship developed in the Department of Organic Chemistry at the Universidad de Murcia. Each year, the Faculty of Chemistry offers students to carry out an internship in one of its departments. This internship has up to three assigned credits per year (equivalent to 30 hours),

granting participants a certificate that is included as an additional merit in their academic records. Along this internship the students can apply classroom theory to research laboratory experiments. The organic chemistry experience reported herein was carried out by upper-division undergraduate students (students in the last years of Bachelor's Degree).

The current lab experience has been designed as a research project whereby every student conducts different experiments and after the analysis of the results the students conclude not only the most appropriate methodology to synthesize the rotaxane but also why the reaction conditions greatly influence the reaction outcome. The appealing aesthetic of rotaxanes and their challenging synthesis constitute an extra stimulus for the students to be engaged into the experiment.

This laboratory experiment addresses important concepts of organic chemistry: amide chemistry, supramolecular chemistry, mechanical bond and, also, structural determination techniques such as FT-IR and NMR. Instructors may use the free time during sessions to remind students of these topics, as well as to teach/recall spectroscopy techniques.

The final organization of the content is described below:

Day 1. Seminar.

The objective of the first session is the establishment of the main goal of the practice, i.e., the search for the best reaction conditions for the synthesis of a [2]rotaxane. Before carrying out the experiments, an introductory session is planned, including a power point presentation entitled "Mechanically Interlocked Molecules and the particular case of rotaxanes" to introduce students to the field of interlocked molecules. In this session, the instructor will help the students to interpret the spectroscopic data (IR and ^{1}H and ^{13}C NMR) of the starting material **2**. The resolution of the pre-labs will be discussed with the students in a short debate.

The instructor is responsible to answer the questions proposed by students. Subsequently, the instructor should emphasize the importance of wearing the protective equipment while performing the experiment and the hazards of the chemicals.

Day 2. Lab Session 1.

This class involves the preparation and isolation of a fumaramide-based [2]rotaxane using the reaction conditions assigned by the instructor. Each student will carry out the reaction under three different conditions and isolate the product obtained from each

experiment. After confirmation of the right outcome of the reaction by TLC by comparing the Rf of the obtained product with an authentic sample, students will measure the melting point of one of them, perform an IR spectrum and prepare a sample for MNR analysis.

Prior to next session the students will receive the spectra of their products in order to make a tentative assignment.

Day 3. Lab Session 2.

The results of all the students' experiments will be compared, and the best reaction conditions will be determined. The students would be encouraged to debate the reasons why the selected reaction conditions are the optimum for the synthesis of [2]rotaxane **1** on the basis of the background received.

Finally, the complete structural determination of the interlocked product will be discussed in depth.

5 Safety and Hazards

Appropriate personal protective equipment, such as disposable gloves, goggles, closed shoes, and a lab coat, must be worn. Procedures must be performed in a fume hood or a similarly ventilated workspace. Liquid and solid waste must be disposed into sealed and appropriately labeled containers. The rinsed syringe should be disposed into an appropriately labelled disposal container. The rinsed needle can be reused after being dried in an oven.

Safety information for all reagents is available via the appropriate SDS.

The Chemical Abstracts Service (CAS) Numbers and Globally Harmonized System (GHS) Hazards of the chemical compounds and solvents used in this work are listed below:

Table S1. Hazards of the chemical compounds employed in the laboratory experiment.

Chemical Compound	CAS Number	GHS Hazards
Celite	68855-54-9	H372: Causes damage to organs (Lungs) through prolonged or repeated exposure if inhaled.

Hydrochloric acid	7647-01-0	H314: Causes severe skin burns and eye damage.<br>H331: Toxic if inhaled.
Isophthaloyl dichloride	99-63-8	H312: Harmful in contact with skin.<br>H314: Causes severe skin burns and eye damage.<br>H318: Causes serious eye damage.<br>H331: Toxic if inhaled.
Magnesium sulphate anhydrous	7487-88-9	H302: Harmful if swallowed.<br>H312: Harmful in contact with skin.<br>H332: Harmful if inhaled.
Sodium chloride	7647-14-5	H319: Causes serious eye irritation.
Sodium hydroxide	1310-73-2	H314: Causes severe skin burns and eye damage.
Triethylamine	121-44-8	H225: Highly flammable liquid and vapor.<br>H302: Harmful if swallowed.<br>H312: Harmful in contact with skin.<br>H314: Causes severe skin burns and eye damage.<br>H332: Harmful if inhaled.
p-Xylylenediamine	539-48-0	H314: Causes severe skin burns and eye damage
Fumaryl chloride	627-63-4	H302 + H312: Harmful if swallowed or in contact with skin.<br>H314: Causes severe skin burns and eye damage.

1-(*N*-Boc-aminomethyl)-4-(aminomethyl)benzene	108468-00-4	H315: Causes skin irritation.<br>H319: Causes serious eye irritation.<br>H335: May cause respiratory irritation.
Trifluoroacetic acid	76-05-1	H314: Causes severe skin burns and eye damage.<br>H332: Harmful if inhaled.<br>H412: Harmful to aquatic life with long lasting effects.

Table S2. Hazards of the solvents employed in the laboratory experiment.

**Solvent**	**CAS Number**	**GHS Hazards**
Acetone	67-64-1	H225: Highly Flammable liquid and vapor.<br>H319: Causes serious eye irritation.<br>H336: May cause drowsiness or dizziness.
Chloroform	67-66-3	H302: Harmful if swallowed.<br>H315: Causes skin irritation.<br>H319: Causes serious eye irritation.<br>H331: Toxic if inhaled.<br>H351: Suspected of causing cancer.<br>H361d: May damage the unborn child.<br>H372: Causes damage to organs through prolonged or repeated exposure.

Chloroform-*d*	865-49-6	H302: Harmful if swallowed.<br><br>H315: Causes skin irritation.<br><br>H319: Causes serious eye irritation.<br><br>H331: Toxic if inhaled.<br><br>H336: May cause drowsiness or dizziness.<br><br>H351: Suspected of causing cancer.<br><br>H361: Suspected of damaging fertility or the unborn child.<br><br>H372: Causes damage to organs through prolonged or repeated exposure.<br><br>H373: Causes damage to organs through prolonged or repeated exposure.
Diethyl ether	60-29-7	H224: Extremely flammable liquid and vapour<br>H302: Harmful if swallowed<br>H336: May cause drowsiness and dizziness
Dichloromethane	75-09-2	H315: Causes skin irritation.<br>H319: Causes serious eye irritation.<br>H336: May cause drowsiness or dizziness.<br>H351: Suspected of causing cancer.
Hexane	110-54-3	H225: Highly flammable liquid and vapor.

		H304: May be fatal if swallowed and enters airways.<br>H315: Causes skin irritation.<br>H336: May cause drowsiness or dizziness.<br>H361f: Suspected of damaging fertility.<br>H373: May cause damage to organs (Nervous system) through prolonged or repeated exposure if inhaled.<br>H411: Toxic to aquatic life with long lasting effects.
Ethyl acetate	141-78-6	H225 Highly flammable liquid and vapor.<br>H319 Causes serious eye irritation.<br>H336 May cause drowsiness or dizziness.
Methanol	67-56-1	H225: Highly flammable liquid and vapor.<br>H301 + H311 + H331: Toxic if swallowed, in contact with skin or if inhaled.<br>H370: Causes damage to organs (Eyes, Central nervous system).

6 General Experimental Information

All commercially available compounds were purchased from Merck, Acros Organics, or Alfa-Aesar Chemical Co. and used without purification. *N*[1],*N*[1],*N*[4],*N*[4]-Tetrabutylfumaramide (**2**) was prepared from fumaroyl dichloride and dibutylamine[21] and the pre-formed U, *N*[1],*N*[3]-bis(4-(aminomethyl)benzyl)isophthalamide (**5**), was generated

in situ by the acidic deprotection of the di-Boc derivative,[22] following previously described procedures. HPLC grade solvents (Scharlab) were nitrogen saturated and were dried and deoxygenated using an Innovative Technology Inc. Pure-Solv 400 Solvent Purification System. Deionized water was used in the preparation of all aqueous solutions. Brine refers to aqueous sodium chloride solution (saturated).

TLC was performed on precoated silica gel on aluminum cards (0.25 mm thick, with fluorescent indicator 254 nm, Fluka) and observed under UV light (254 or 365 nm).

The **additions** were made with kd Scientific motor-driven syringe pumps (model number 101) and stainless steel 304 syringe needle, noncoring point (30 cm) from Merck.

Under reduced pressure refers to the use of a Büchi Rotavapor R-3000 or Heidolph Hei-Vap Value G3 apparatus with a Vacuumbrand CVC 3000 vacuum pump with a water bath to remove solvent.

Melting point (m.p.) was determined on a Kofler hot-plate melting point apparatus and is uncorrected.

Infrared spectroscopy was performed using a PerkinElmer Spectrum 65, FT-IR Spectrometer (ATR) in the range 4000-600 cm^{-1}. The intensities of the absorption bands are indicated as vs (very strong), s (strong), m (middle) and w (weak).

NMR spectra were recorded at 298 K using a Bruker Avance 400 instrument (400 MHz ^{1}H frequency, 101 MHz ^{13}C frequency). Chemical shifts are quoted in parts per million (ppm), referenced to residual chloroform (7.26 ppm for ^{1}H NMR, 77.00 ppm for ^{13}C NMR) as internal standard, and coupling constants, *J*, are quoted in Hz. Multiplicities quoted as: singlet (s), doublet (d), triplet (t), quartet (q) and multiplet (m). Signals in the ^{1}H and ^{13}C NMR spectra of the synthesized compounds were assigned with the aid of DEPT, APT, or two-dimensional NMR experiments (COSY, HMQC, HMBC and NOESY). All NMR data were processed using the MestReNova software (from MestreLab Research S. L.).

7 Experimental Procedure for the five-component synthesis of [2]rotaxane 1[23]

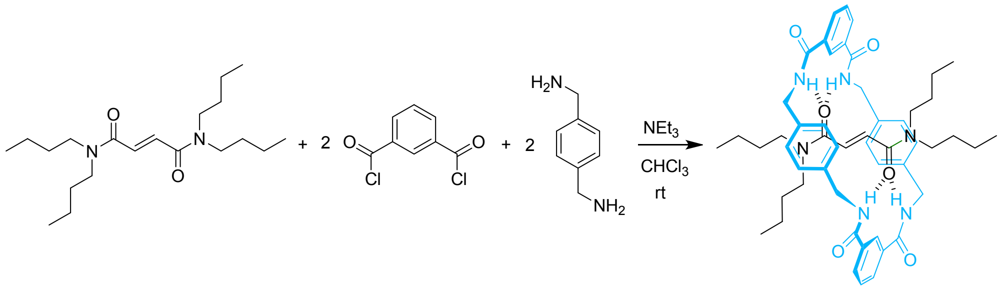

2 **3** **4** **1**

A solution of thread **2** (x equiv.) and Et_3N (z / 2 equiv.) in $CHCl_3$ (n - 40 mL) was stirred vigorously while solutions of *p*-xylylenediamine (**4**) (y equiv.) and Et_3N (z/2 equiv.) in $CHCl_3$ (20 mL) and isophthaloyl dichloride (**3**) (y equiv.) in $CHCl_3$ (20 mL) were simultaneously added using a motor-driven syringe pump or two independent dropping funnels. After finishing the reaction, the resulting suspension was filtered through a Celite pad and the filtrate was washed with water (2 × 50 mL), a solution of HCl 1 M (2 × 50 mL), a solution of NaOH 1 M (2 × 50 mL) and brine (2 × 50 mL). The organic phase was then dried with $MgSO_4$, filtered and then, the solvent was removed under reduced pressure. The resulting solid was filtered and washed with diethyl ether (3 x 15 mL) until all the unreacted thread **2** was removed (R_f = 0.39 for **1**, R_f = 0.70 for **2**; $CHCl_3$/acetone 9:1) (Figure S5) and then, was dried under vacuum.

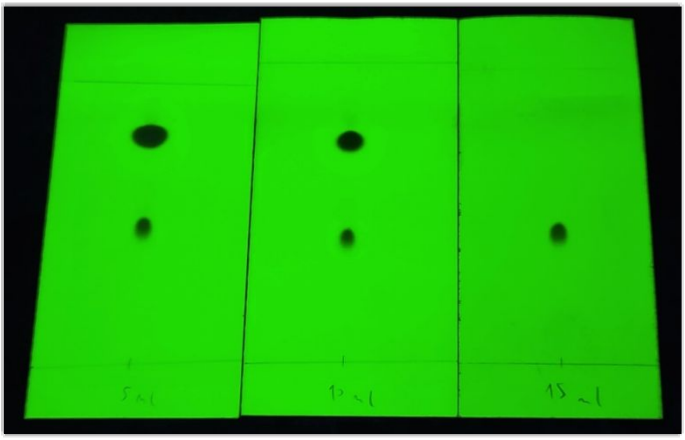

(a) (b) (c)

Figure S6. *TLC of the solid washed with different volumes of diethyl ether: (a) 5 mL, (b) 10 mL and (c) 15 mL.*

Table S3. Screening of the reaction conditions.

Entry	2 (x equiv)	3 and 4 (y equiv)	Et_3N (z equiv)	$CHCl_3$ (n mL)	Addition time (min)	Yield[a] (%)	Addition method
1	1	8	24	90	30	13-17	Syringe pump
2	1	8	24	90	60	18-20	Syringe pump
3	1	8	24	90	90	21-25	Syringe pump
4	1	8	24	180	30	29-33	Syringe pump
5	1	8	24	180	60	37-39	Syringe pump
6	1	8	24	180	90	40-42	Syringe pump
7	1	8	24	360	90	69-71	Syringe pump
8	8	1	8	90	90	10-12	Syringe pump
9	8	1	8	180	90	12-13	Syringe pump
10	1	8	8	90	240	47-50	Dropping funnels
11	1	8	8	180	240	57-59	Dropping funnels
12	1	8	8	360	240	64-67	Dropping funnels

[a]Range of yields obtained by the students

8 Experimental Procedure for the three-component synthesis of [2]rotaxane 1

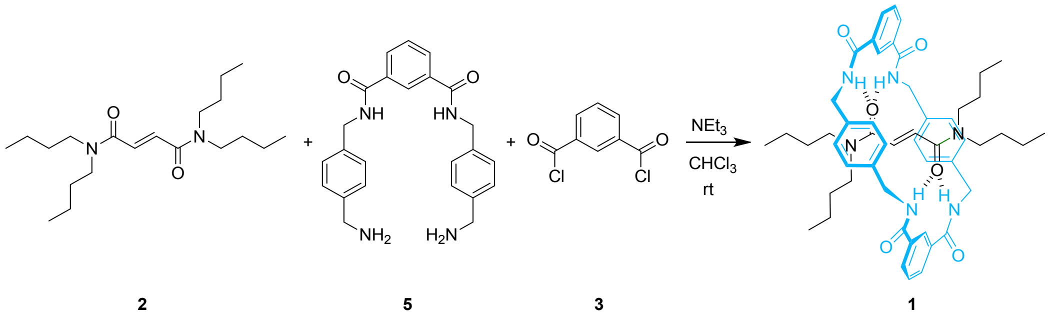

The thread **2** (x equiv.), the pre-formed U **5** (y equiv.) and Et_3N (z equiv) in $CHCl_3$ (n-20 mL) were stirred vigorously while a solution of isophthaloyl dichloride (**3**) (y equiv.) in $CHCl_3$ (20 mL) was added using motor-driven syringe pumps. After finishing the reaction, the resulting suspension was filtered through a Celite pad and the filtrate was washed with water (2 × 50 mL), a solution of HCl 1 M (2 × 50 mL), a solution of NaOH 1 M (2 × 50 mL) and brine (2 × 50 mL). The organic phase was then dried with $MgSO_4$, filtered and then, the solvent was removed under reduced pressure. The resulting solid was filtered and washed with diethyl ether (3 x 15 mL) until all the unreacted thread **1** was extracted (R_f = 0.39 for **1**, R_f = 0.70 for **2**; $CHCl_3$/acetone 9:1) and then, was dried under vacuum.

Table S4. Screening of the reaction conditions.

Entry	2 (x equiv.)	3 and 5 (y equiv.)	Et_3N (z equiv.)	$CHCl_3$ (n mL)	addition time (min.)	Yield[a] (%)
1	1	8	24	180	90	51-52
2	8	1	8	180	90	52-56

[a]Range of yields obtained by the students

Data of rotaxane **1**:

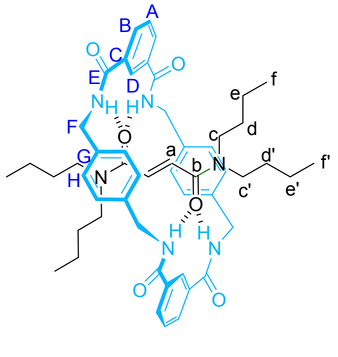

White solid; m.p. >300 ºC; IR (ATR): 3368 (w, NH), 2963 (w, =C-H), 2930 (w, =C-H), 2863 (w), 1658 (s, C=O), 1596 (m), 1531 (vs, C=C), 1444 (m), 1305 (m), 1271 (m), 1200 (w), 1146 (w), 1075 (w), 950 (w), 855 (w), 809 (m), 728 (m), 684 (m), 622 (m) cm^{-1}; ^{1}H NMR (400 MHz, $CDCl_3$, 298 K): δ = 8.67 (s, 2H, H_D), 8.33 (dd, J = 7.8, 0.9 Hz, 4H, H_B), 7.76 (d, J = 8.3 Hz, 4H, NH), 7.65 (t, J = 7.8 Hz, 2H, H_A), 7.01 (s, 8H, H_H), 5.98 (s, 2H, H_a), 5.27 (dd, J = 14.2, 9.1 Hz, 4H, H_{Feq}), 3.72 (dd, J = 14.2, 1.5 Hz, 4H, H_{Fax}), 3.35-3.26 (m, 4H, $H_{c'}$), 2.97-2.88 (m, 4H, H_c), 1.61-1.51 (m, 4H, $H_{d'}$), 1.38-1.27 (m, 4H, $H_{e'}$), 1.26-1.17 (m, 4H, H_d), 0.93 (t, J = 7.3 Hz, 6H, $H_{f'}$), 0.66-0.55 (m, 10H, H_e + H_f); ^{13}C NMR (101 MHz, $CDCl_3$, 298 K): δ = 165.31 (C_E), 165.04 (C_b), 138.05 (C_G), 133.29 (C_C), 132.12 (C_B), 129.51 (C_A), 128.91 (C_H), 128.24 (C_a), 122.66 (C_D), 48.95 (C_c), 47.86 ($C_{c'}$), 43.37 (C_F), 32.18 (C_d), 30.05 ($C_{d'}$), 20.21 ($C_{e'}$), 19.65 (C_e), 13.76 ($C_{f'}$), 13.60 (C_f).

Structure of possible byproducts:

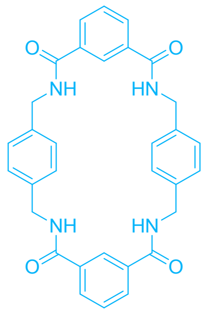

6

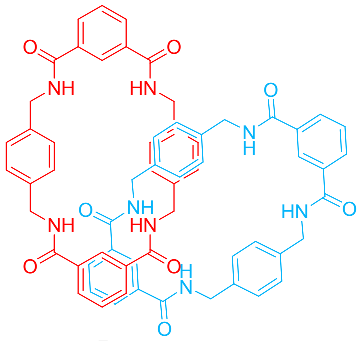

7

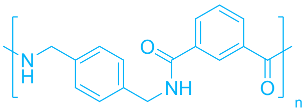

8

9 Synthesis of thread 3 and diamine 5

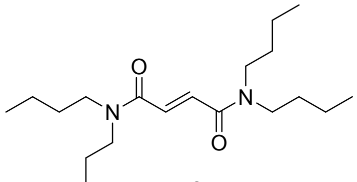

3

To a stirred solution of fumaryl chloride (1 equiv.) in anhydrous CH_2Cl_2 (50 mL per mmol of fumaryl chloride) was added dropwise a solution of dibutylamine (6 equiv.) in CH_2Cl_2 (20 mL per mmol of fumaryl chloride). The reaction mixture was stirred for 1 h and then washed with an aqueous solution of 1 M HCl (2 × 30 mL), saturated $NaHCO_3$ (2 × 30 mL) and brine (30 mL). The organic phase was dried with anhydrous $MgSO_4$ and concentrated under reduced pressure. The resulting residue was subjected to column chromatography (silica gel) using a mixture of

hexane/AcOEt (3:1), giving the title product **3** as a yellow oil (range of yields: 75-84 %).

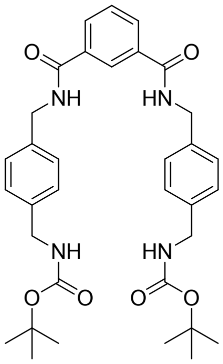

9

To a solution of 1-(*N*-Boc-aminomethyl)-4-(aminomethyl)benzene (2 equiv.) and triethylamine (2.5 equiv.) 7.4 mL, 53.1 mmol) in $CHCl_3$ (7 mL per mmol of *N*-Boc-protected amine) at 0 ºC, a solution of isophthaloyl chloride (1 equiv.) in $CHCl_3$ (75 mL) was added dropwise over 1 hour. The reaction mixture is stirred overnight at room temperature. Collect the precipitate under filtration and wash the resulting solid with diethyl ether to obtain the product **9** as a white product (range of yields: 64-76 %).

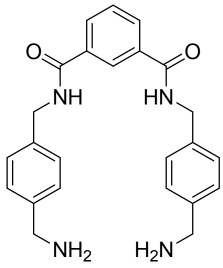

5

Add trifluoroacetic acid (10 equiv.) to a suspension of **9** (1 equiv.) 4.2 g, 6.94 mmol) in CH_2Cl_2 (20 mL per mmol of **9**). The reaction is stirred for 12 hours and, then, concentrated under reduced pressure. The crude is stirred the resulting powder with amberlyst A-21 resin (2.5 equiv.) in a dichoromethane/methanol 1:1 v/v ratio (20 mL per mmol of **9**) for 1 hour and, then filtered to remove the resin. The solvent is concentrated under reduced pressure directly in the flask of the rotaxane reaction, since the product is used directly in the next reaction step without further purification.

10 References

1 C. J. Bruns, J. F. Stoddart. The Mechanical Bond: A Work of Art. *Top. Curr. Chem.* **2011**, *323*, 19-72.

2. J. E. M. Lewis, M. Galli, S. M. Goldup. Properties and Emerging Applications of Mechanically Interlocked Ligands. *Chem. Commun.* **2017**, *53*, 298-312.

3. E. A. Neal, S. M. Goldup. Chemical Consequences of Mechanical Bonding in Catenanes and Rotaxanes: Isomerism, Modification, Catalysis and Molecular Machines for Synthesis. *Chem. Commun.* **2014**, *50*, 5128-5142.

4. S. Erbas-Cakmak, D. A. Leigh, C. T. McTernan, A. L. Nussbaumer. Artificial Molecular Machines. *Chem. Rev.* **2015**, *115*, 10081-10206.

5. D. B. Amabilino, J. F. Stoddart. Interlocked and Intertwined Structures and Superstructures. *Chem. Rev.* **1995**, *95*, 2725-2828.

6. F. Vögtle, T. Dünnwald, T. Schmidt. Catenanes and Rotaxanes of the Amide Type. *Acc. Chem. Res.* **1996**, *29*, 451-460.

7. J. E. Beves, B. A. Blight, C. J. Campbell, D. A. Leigh, R. T. McBurney. Strategies and Tactics for the Metal-Directed Synthesis of Rotaxanes, Knots, Catenanes and Higher Order Links. *Angew. Chem. Int. Ed.* **2011**, *50*, 9620-9327.

8. N. H. Evans. Recent Advances in the Synthesis and Application of Hydrogen Bond Templated Rotaxanes and Catenanes. *Eur. J. Org. Chem.* **2019**, 3320-3343.

9. L. Pauling. The Structure and Entropy of Ice and of Other Crystals with Some Randomness of Atomic Arrangement. *J. Am. Chem. Soc.* **1935**, *57*, 2680-2684.

10. P. A. Kollman, L. C. Allen. The Theory of the Hydrogen Bond. *Chem. Rev.* **1972**, *72*, 283-303.

11. J. F. Stoddart. The Chemistry of the Mechanical Bond. *Chem. Soc. Rev.* **2009***, 38*, 1802-1820.

12. M. Xue, Y. Yang, X. Chi, X. Yan, F. Huang. Development of Pseudorotaxanes and Rotaxanes: From Synthesis to Stimuli-Responsive Motions to Applications. *Chem. Rev.* **2015**, *115*, 7398-7501.

13. I. T. Harrison, S. Harrison. Synthesis of a stable complex of a macrocycle and a threaded chain. *J. Am. Chem. Soc.* **1967**, *89*, 5723-5724.

14. G. Schill, H. Zollenkopf, Rotaxan-Verbindungen, I. *Justus Liebigs Ann. Chem.* **1969**, *721*, 53-74.

15. F. G. Gatti, D. A. Leigh, S. A. Nepogodiev, A. M. Z. Slawin, S. J. Teat, J. K. Y. Wong. Stiff and Sticky in the Right Places: The Dramatic Influence of Preorganizing Guest Binding Sites on the Hydrogen Bond-Directed Assembly of Rotaxanes. *J. Am. Chem. Soc.* **2001**, *123*, 5983-5989.

16. R. Hoss, F. Vögtle. Template Syntheses. *Angew. Chem. Int. Ed. Engl.* **1994**, *33*, 375-384.

17. J. A. Mears, L. L. Lackner, S. Fang, E. Ingerman, J. Numari, J. E. Hinshaw. Conformational Changes in Dnm1 Support a Contactile Mechanism for Mitocondrial Fission. *Nat. Struct. Mol. Biol.* **2011**, *18*, 20-26.

18. E. Gouaux, R. MacKinnon. Principles of Selective Ion Transport in Channels and Pumps. *Science* **2005**, *310*, 1461-1465.

19. A. Tonath. Hibernating Bears, Antibiotics and the Evolving Ribosome. *Angew. Chem. Int. Ed.* **2010**, *49*, 4340-4354.

20. K. N. Kreuzer, N. R. Cozzarelli. Formation and Resolution of DNA Catenanes by DNA Gyrase. *Cell* **1980**, *20*, 245-254.

21. C. Lopez-Leonardo, A. Martinez-Cuezva, D. Bautista, M. Alajarin, J. Berna. Homo and heteroassembly of amide-based [2]rotaxanes using α,α'-dimethyl-*p*-xylylenediamines. *Chem. Commun.* **2019**, *55*, 6787-6790.

22. D. Gonzalez Cabrera, B. D. Koivisto, D. A. Leigh. A metal-complex-tolerant CuAAC 'click' protocol exemplified through the preparation of homo- and mixed-metal-coordinated [2]rotaxanes. *Chem. Commun.* **2007**, 4218-4220.

23. The conditions were modified based on the previously reported work: A. Martinez-Cuezva, F. Morales, G. R. Marley, A. Lopez-Lopez, J. C. Martinez-Costa, D. Bautista, M. Alajarin, J. Berna. Thermally and Photochemically Induced Dethreading of Fumaramide-Based Kinetically Stable Pseudo[2]rotaxanes. *Eur. J. Org. Chem.* **2019**, 3480-3488.

11 NMR and IR Spectra of the synthesized compounds

N[1],*N*[1],*N*[4],*N*[4]-tetrabutylfumaramide (**2**) **(**IR-ATR)

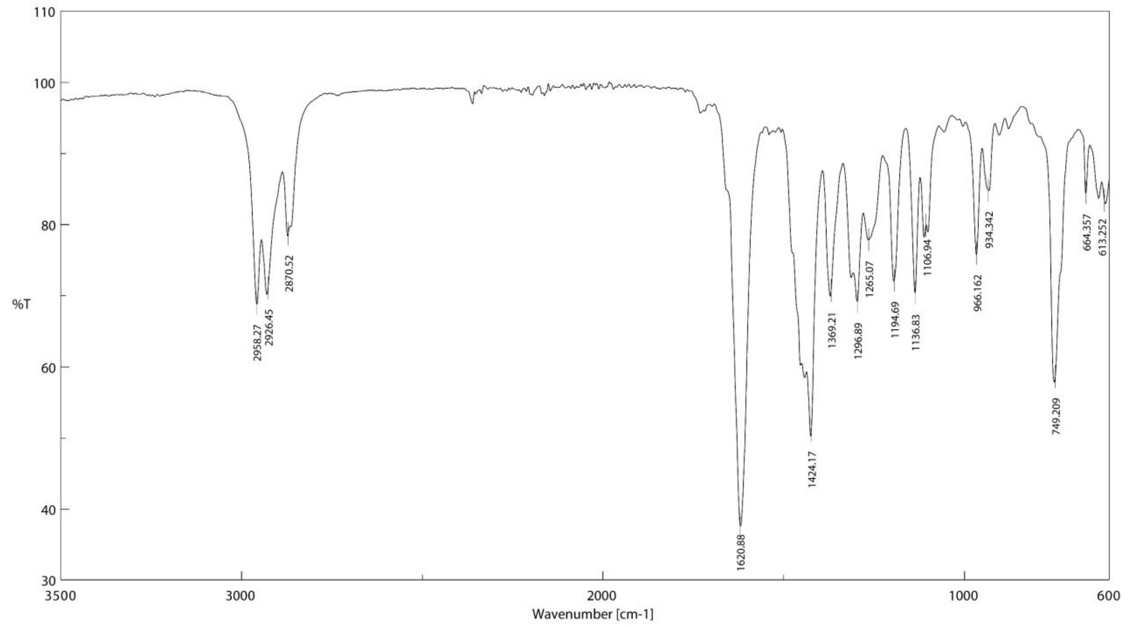

Predicted ^{1}H NMR spectrum of *N*[1],*N*[1],*N*[4],*N*[4]-tetrabutylfumaramide (**2**) using MestReNova software

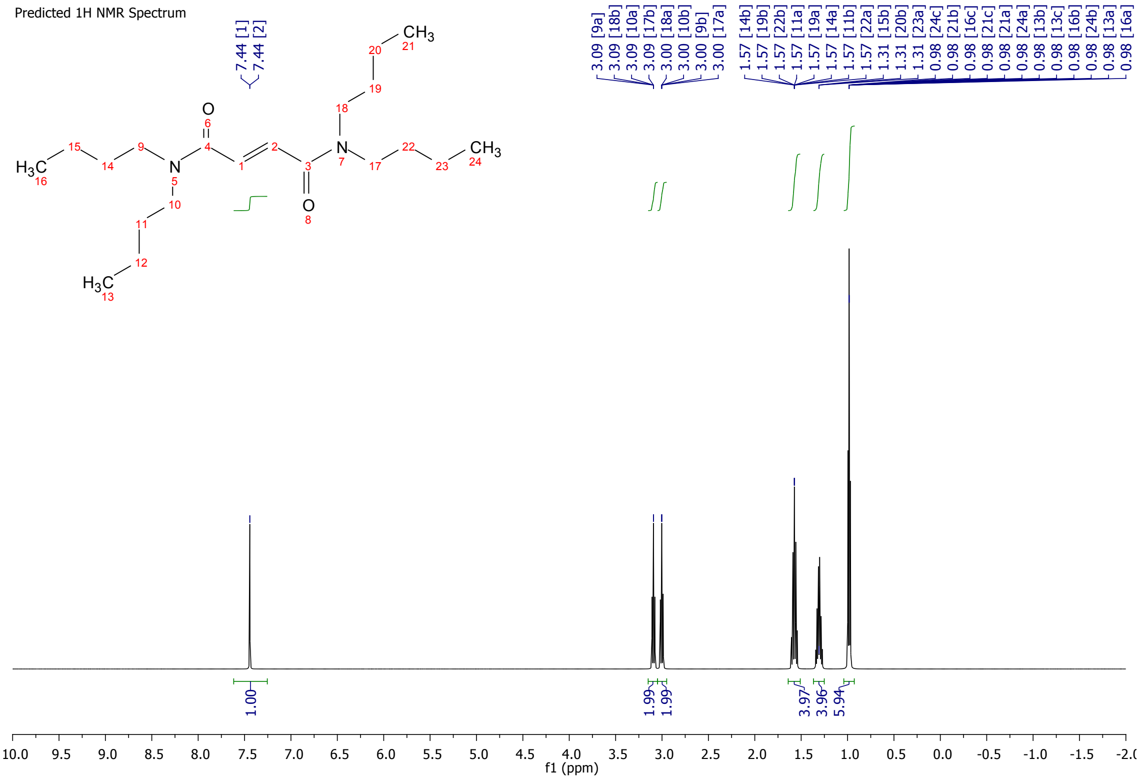

N[1],*N*[1],*N*[4],*N*[4]-tetrabutylfumaramide (**2**) (^{1}H NMR, 400 MHz, $CDCl_3$, 298 K)

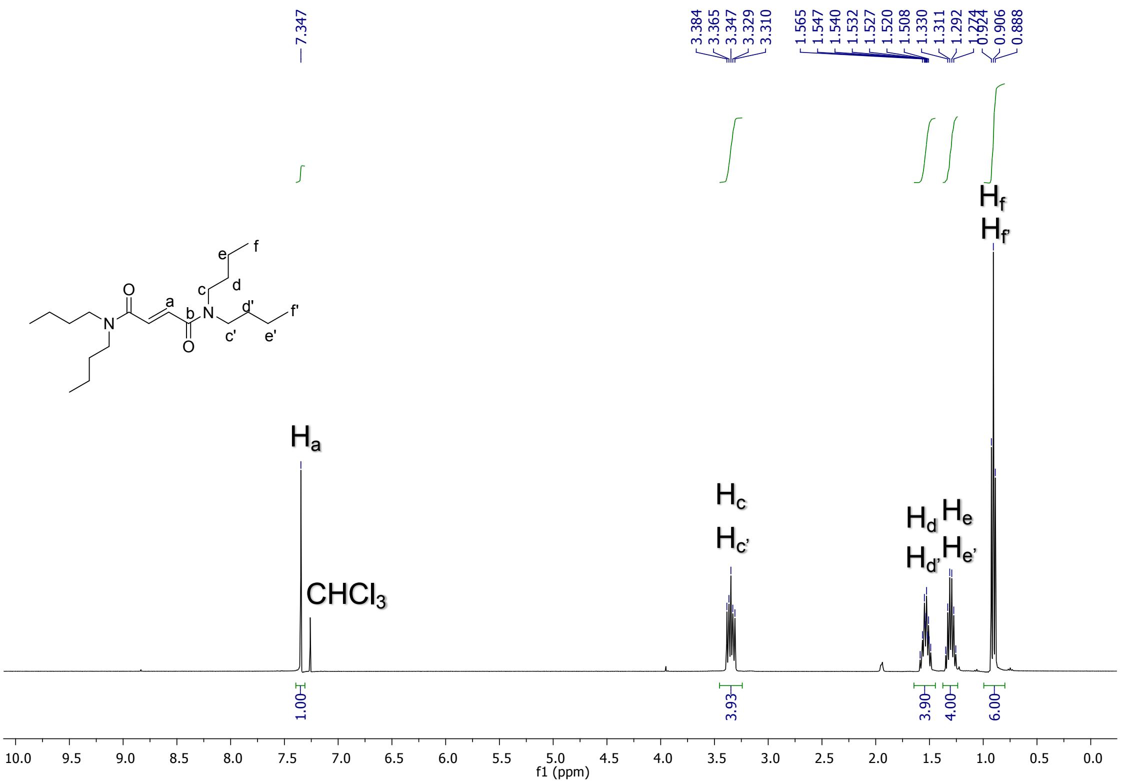

Predicted ^{13}C NMR spectrum of *N*[1],*N*[1],*N*[4],*N*[4]-tetrabutylfumaramide (**2**) using MestReNova software

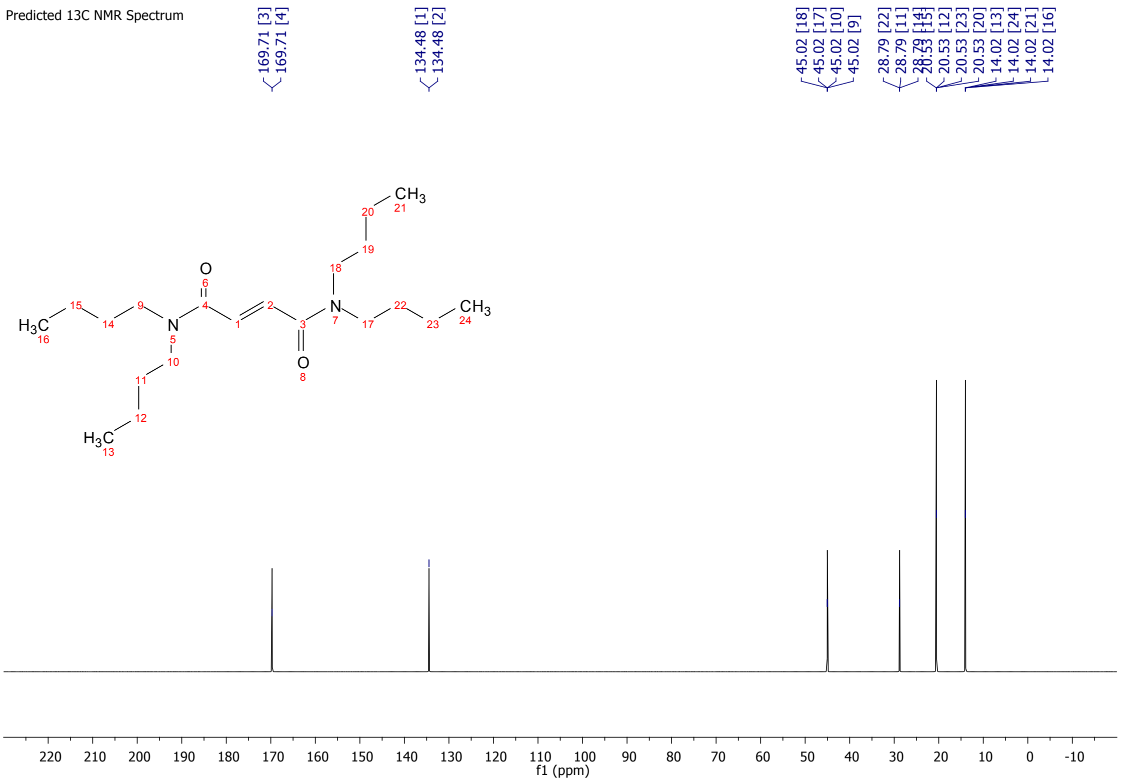

N^1,N^1,N^4,N^4-tetrabutylfumaramide (**2**) (^{13}C NMR, 101 MHz, $CDCl_3$, 298 K)

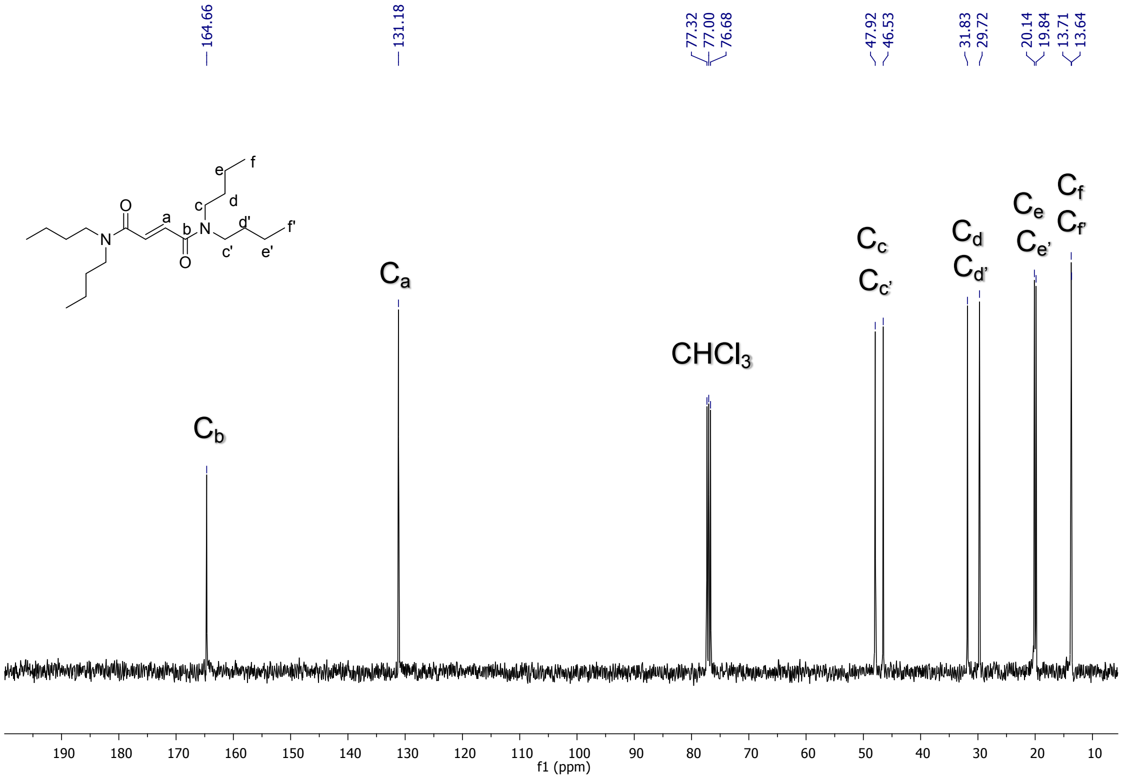

N^1,N^1,N^4,N^4-tetrabutylfumaramide (**2**) (DEPT-135, 101 MHz, $CDCl_3$, 298 K)

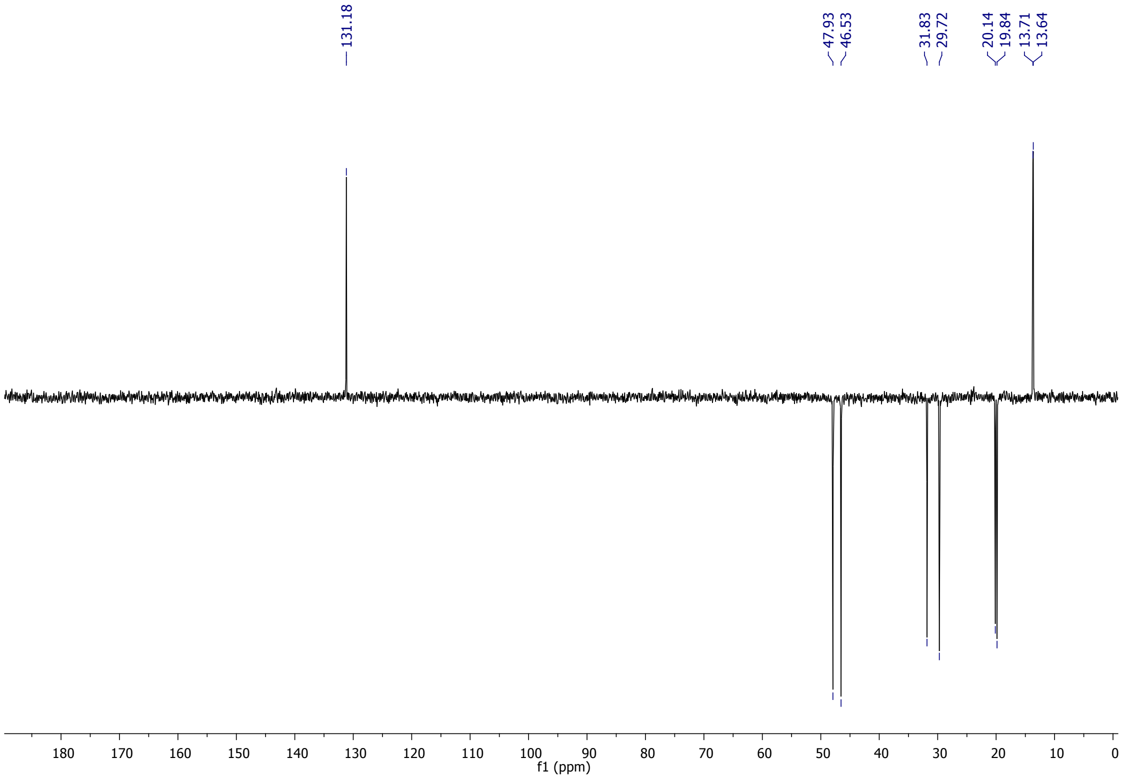

N[1],*N*[3]-bis(4-(aminomethyl)benzyl)isophthalamide (**9**) (IR-ATR)

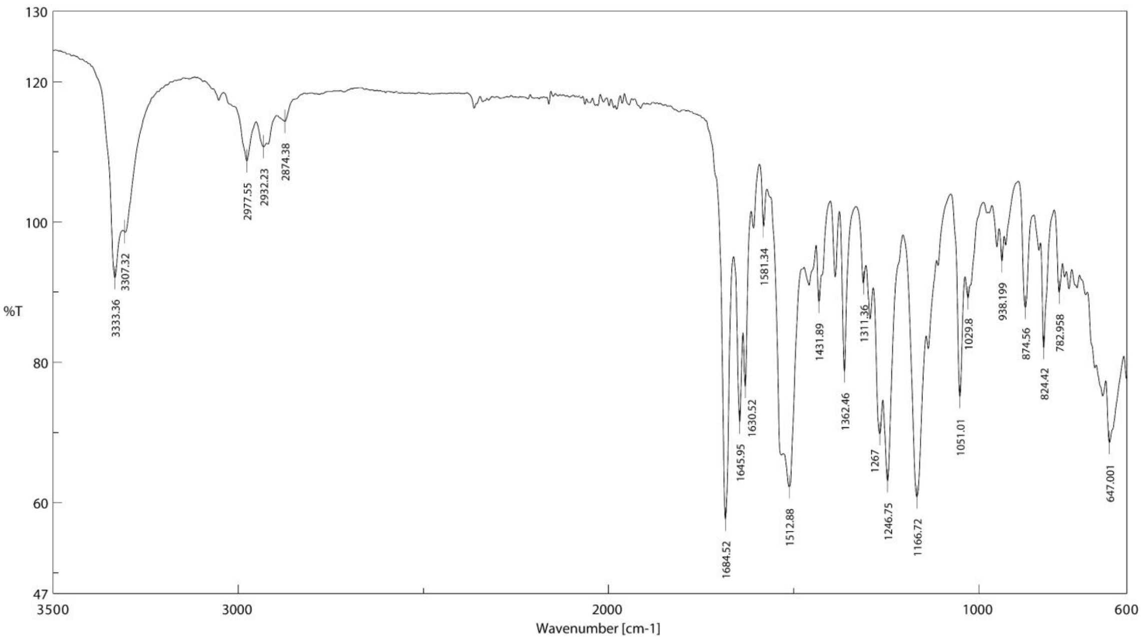

Predicted ^{1}H NMR spectrum of compound **9** using MestReNova software

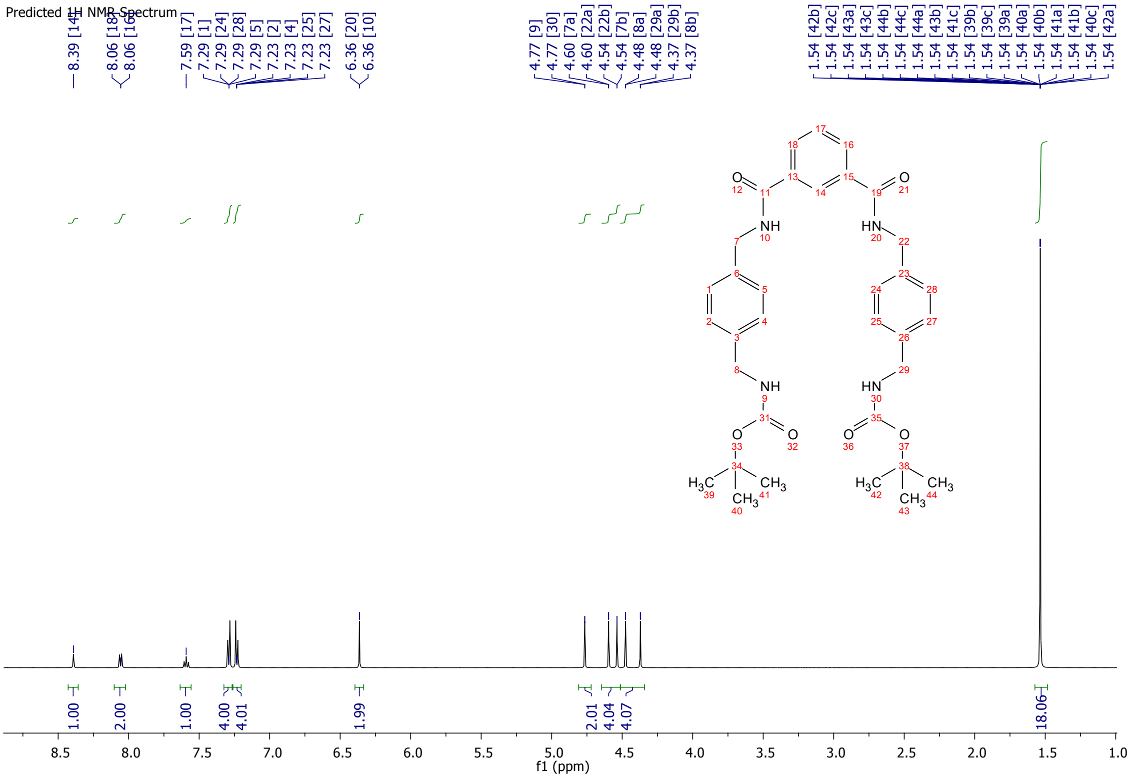

Compound **9** (^1H NMR, 400 MHz, DMSO-d$_6$, 298 K)

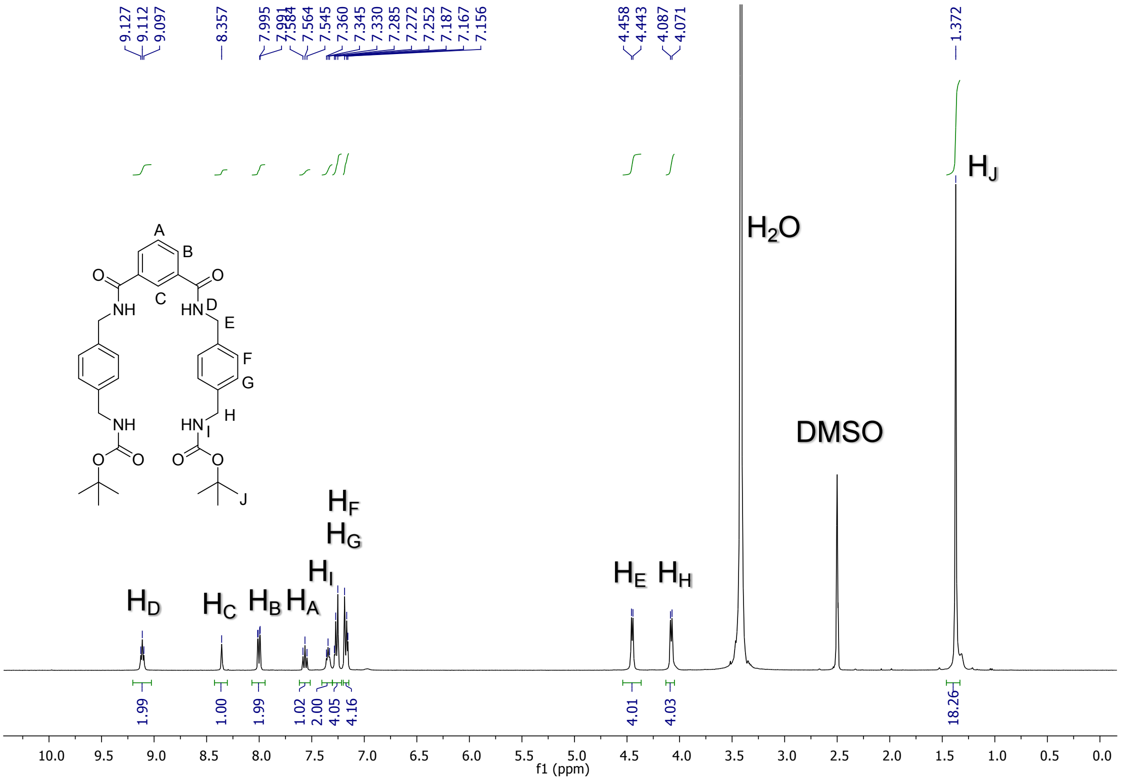

Compound **9** (^1H NMR, 400 MHz, $CDCl_3$, 298 K)

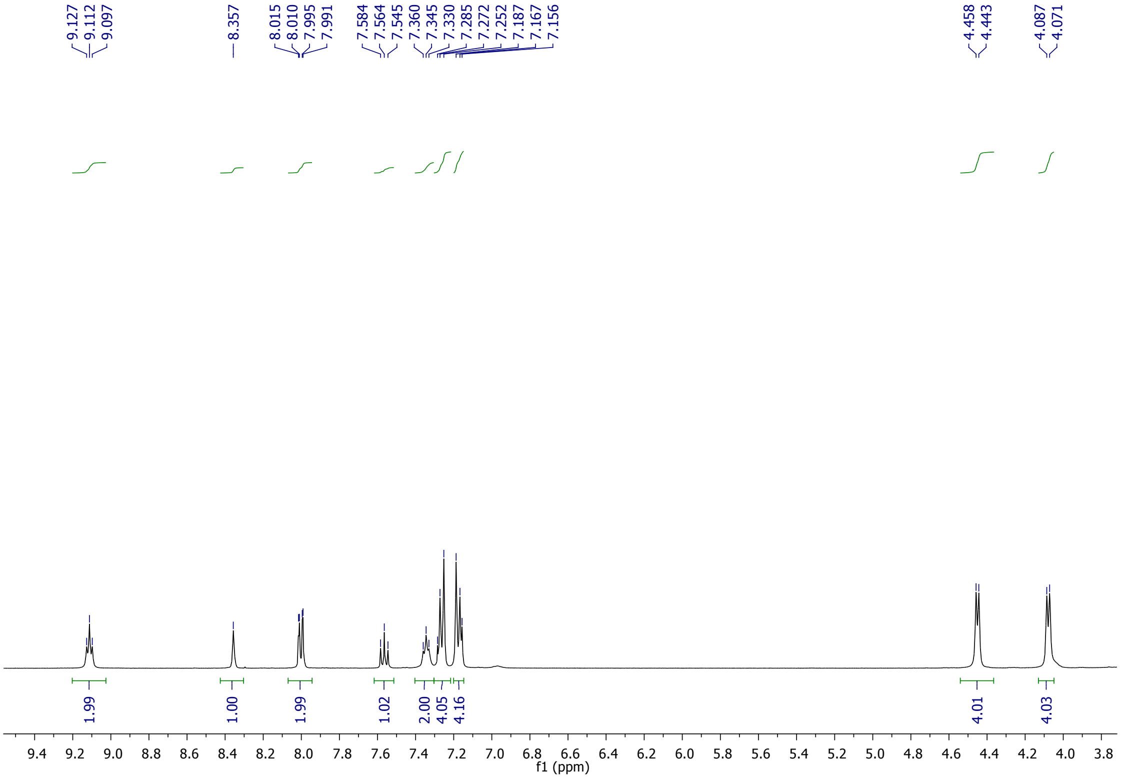

Rotaxane **1 (**IR-ATR)

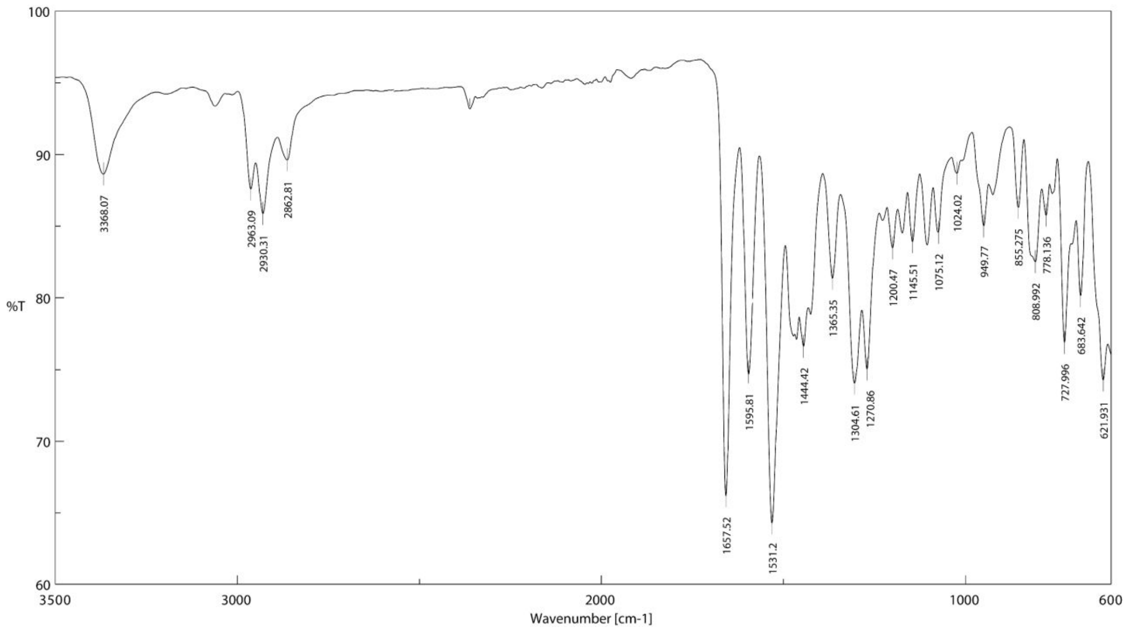

Note: Students can easily distinguish which of the two C=O stretches in the rotaxane IR spectrum is from the thread and which is from the tetralactam macrocycle by the intensity of the peaks. While the macrocycle has 4 carbonyl groups, the thread only has 2.

Rotaxane **1** (^{1}H NMR, 400 MHz, $CDCl_3$, 298 K)

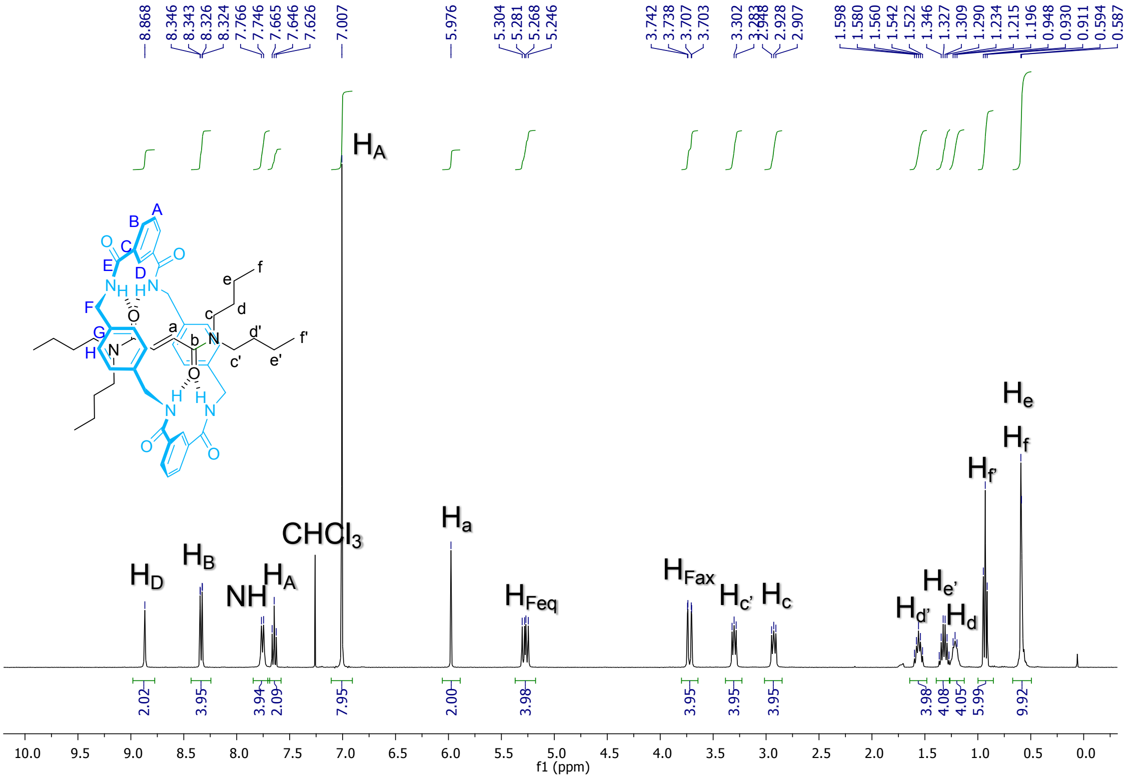

Rotaxane **1** (^{1}H NMR, 400 MHz, $CDCl_3$, 298 K).

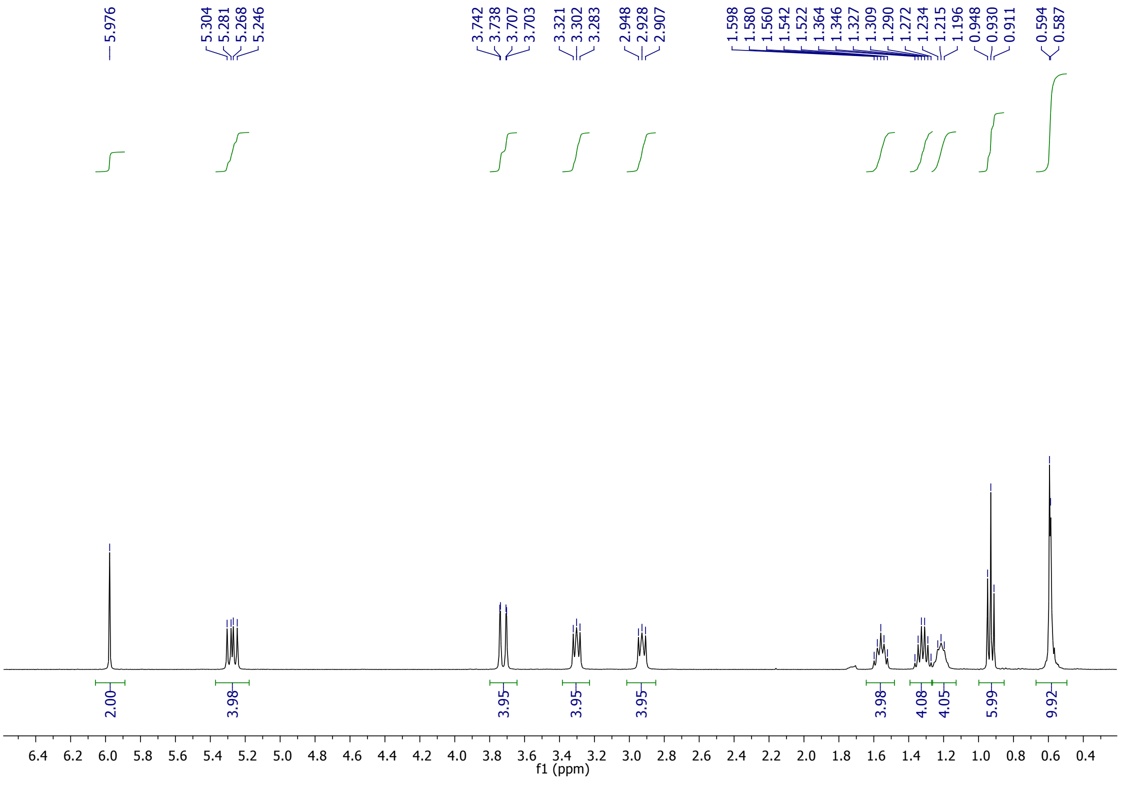

Rotaxane **1** (^1H NMR, 400 MHz, $CDCl_3$, 298 K)

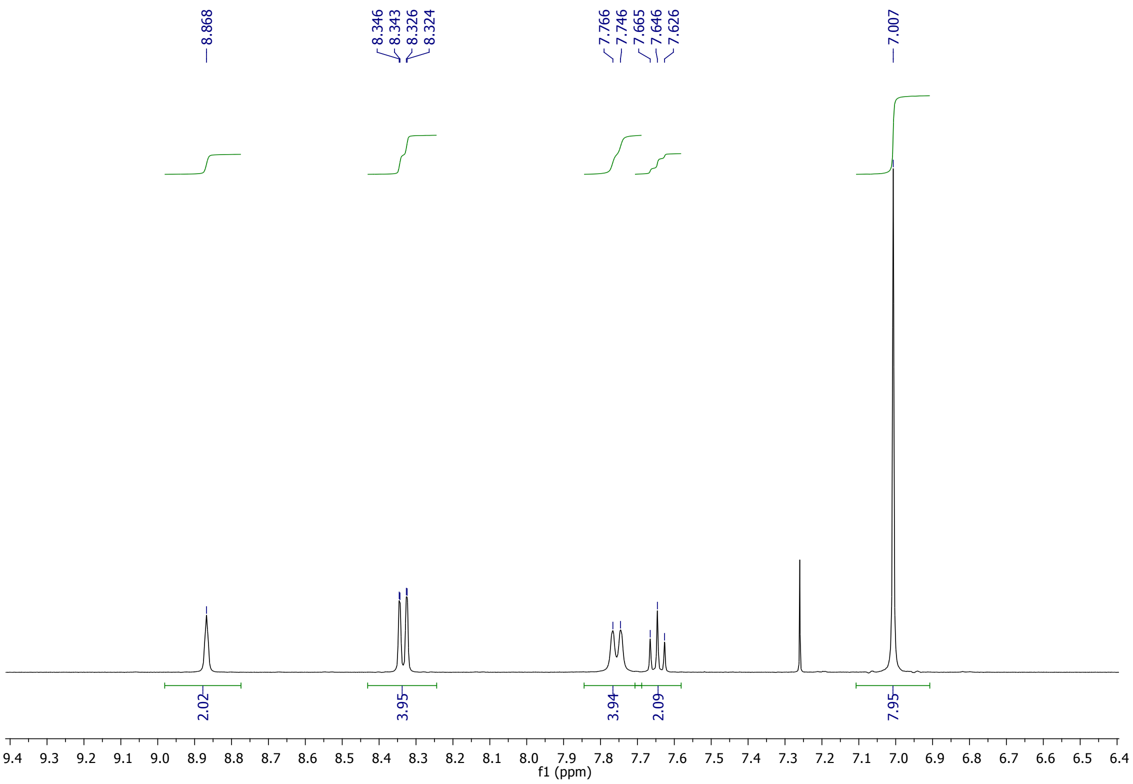

Rotaxane **1** (^{13}C NMR, 101 MHz, $CDCl_3$, 298 K)

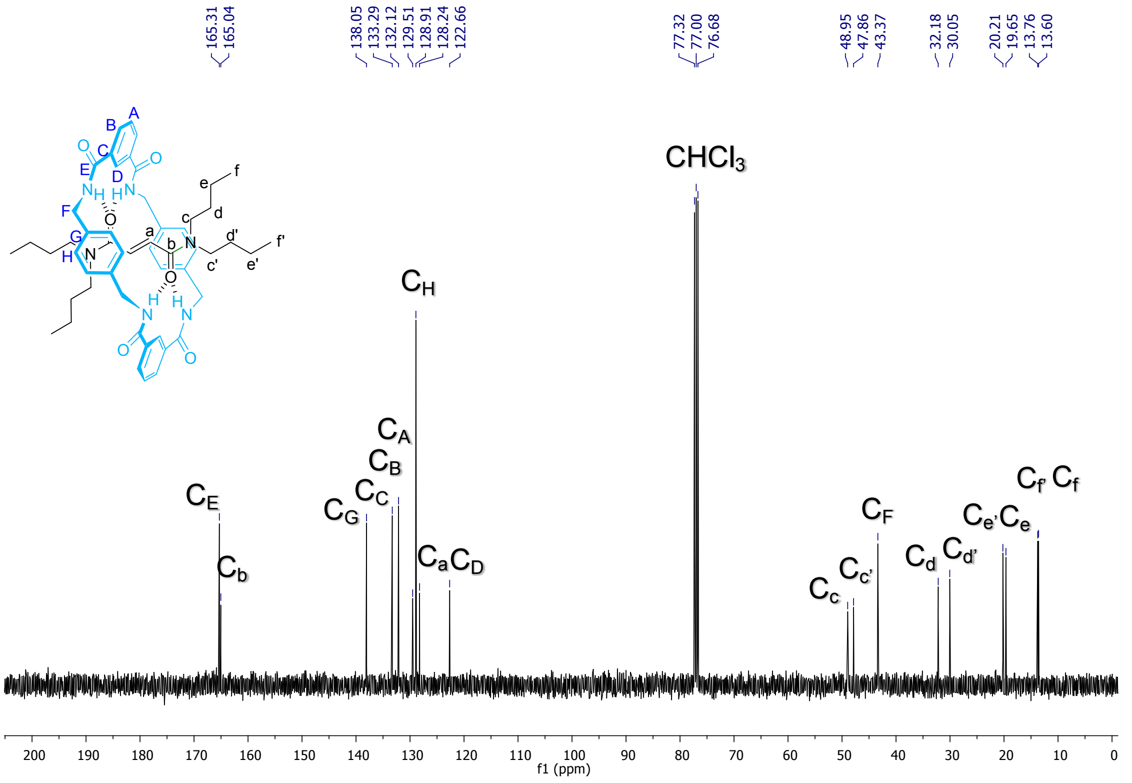

Rotaxane **1** (^{13}C NMR, 101 MHz, $CDCl_3$, 298 K)

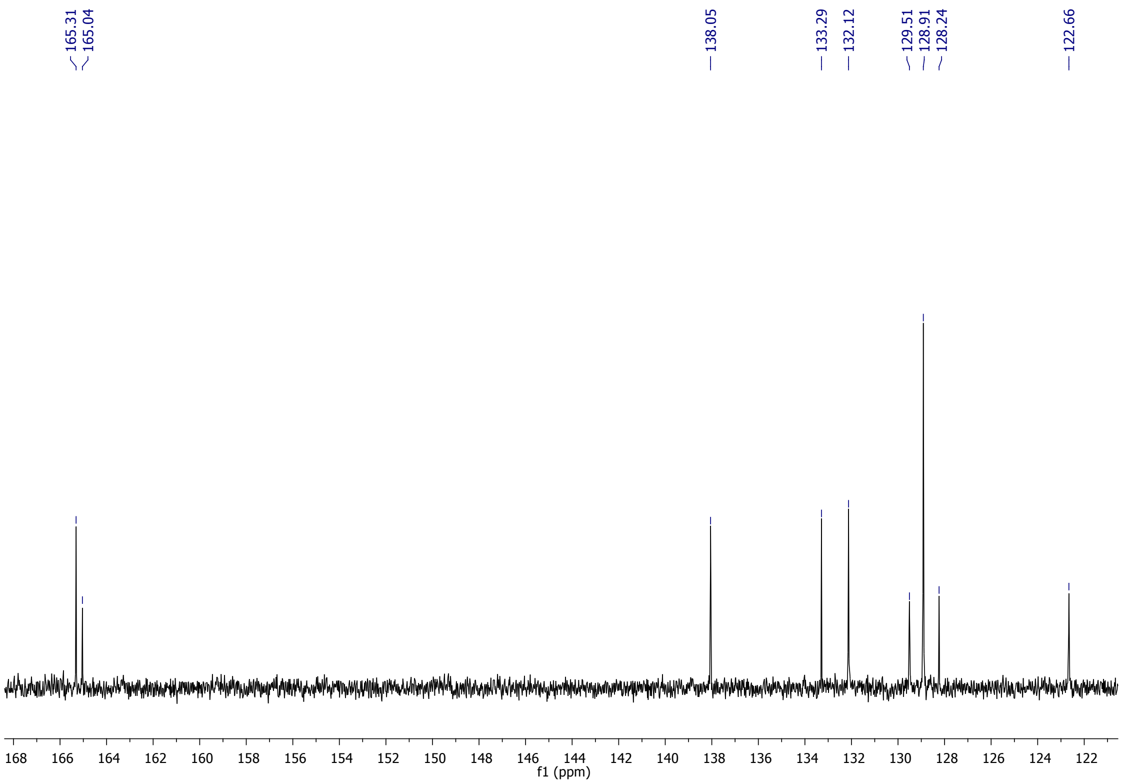

Rotaxane **1** (^{13}C NMR, 101 MHz, $CDCl_3$, 298 K)

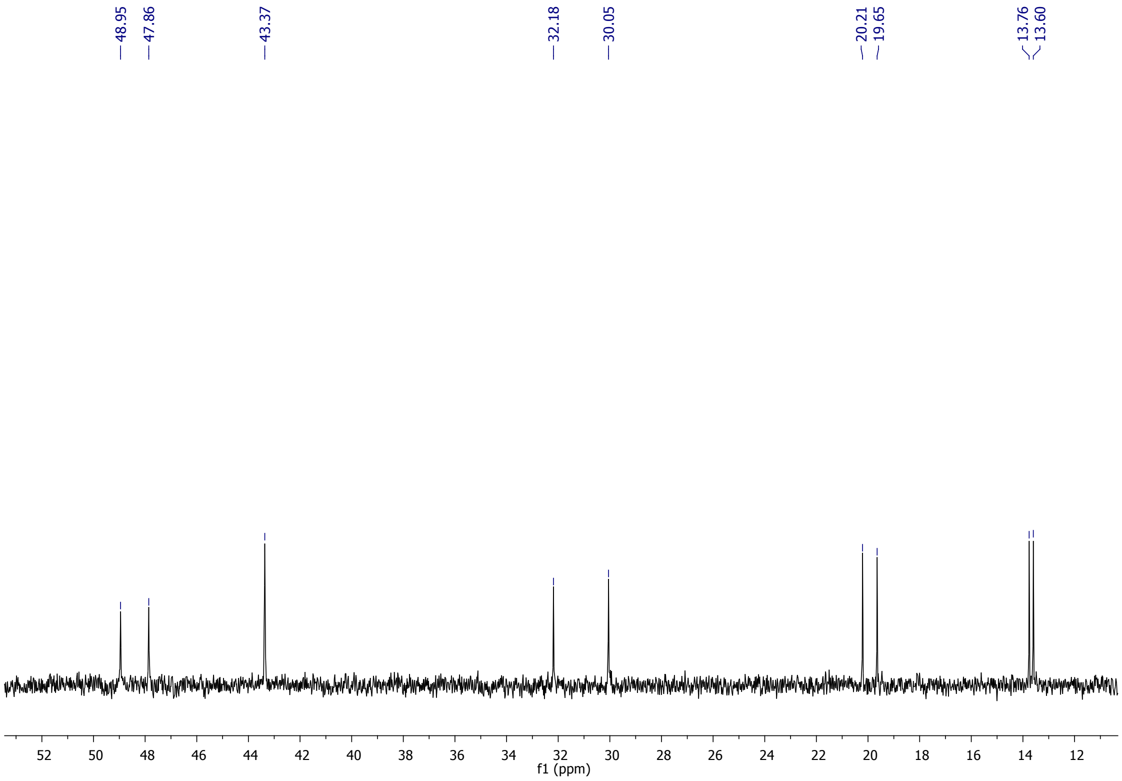

Rotaxane **1** (DEPT-135, 101 MHz, $CDCl_3$, 298 K)

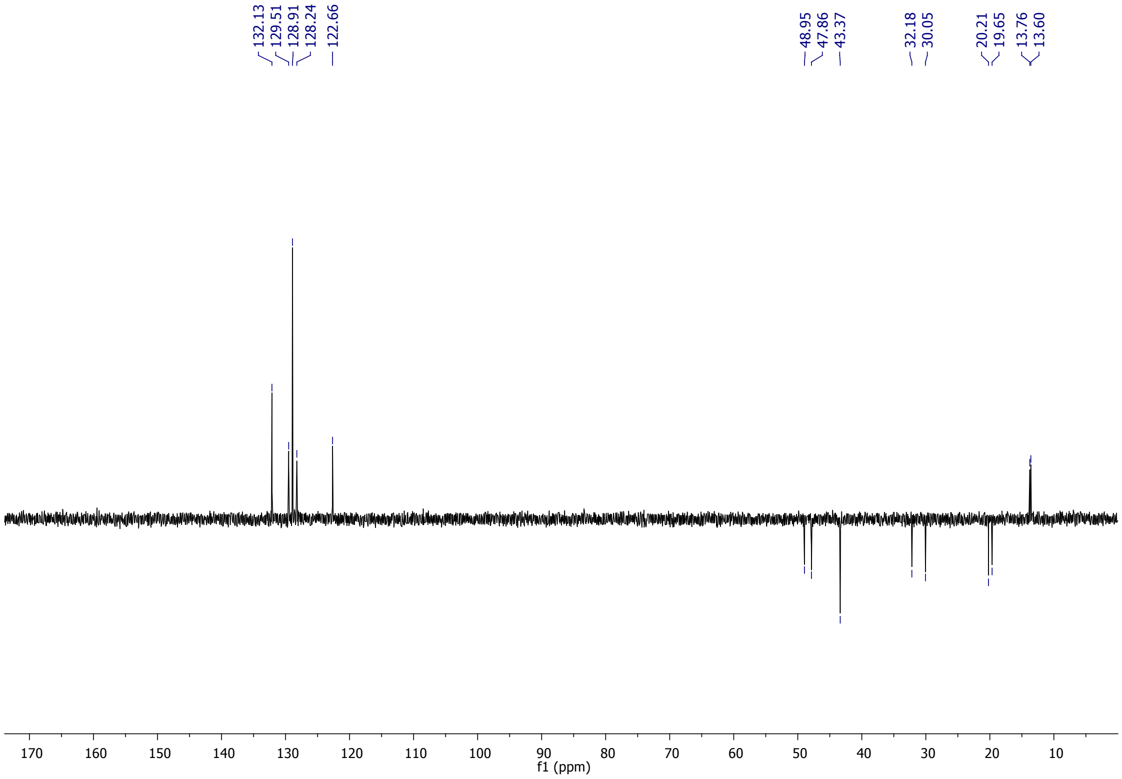

Rotaxane **1** (APT, 101 MHz, $CDCl_3$, 298 K)

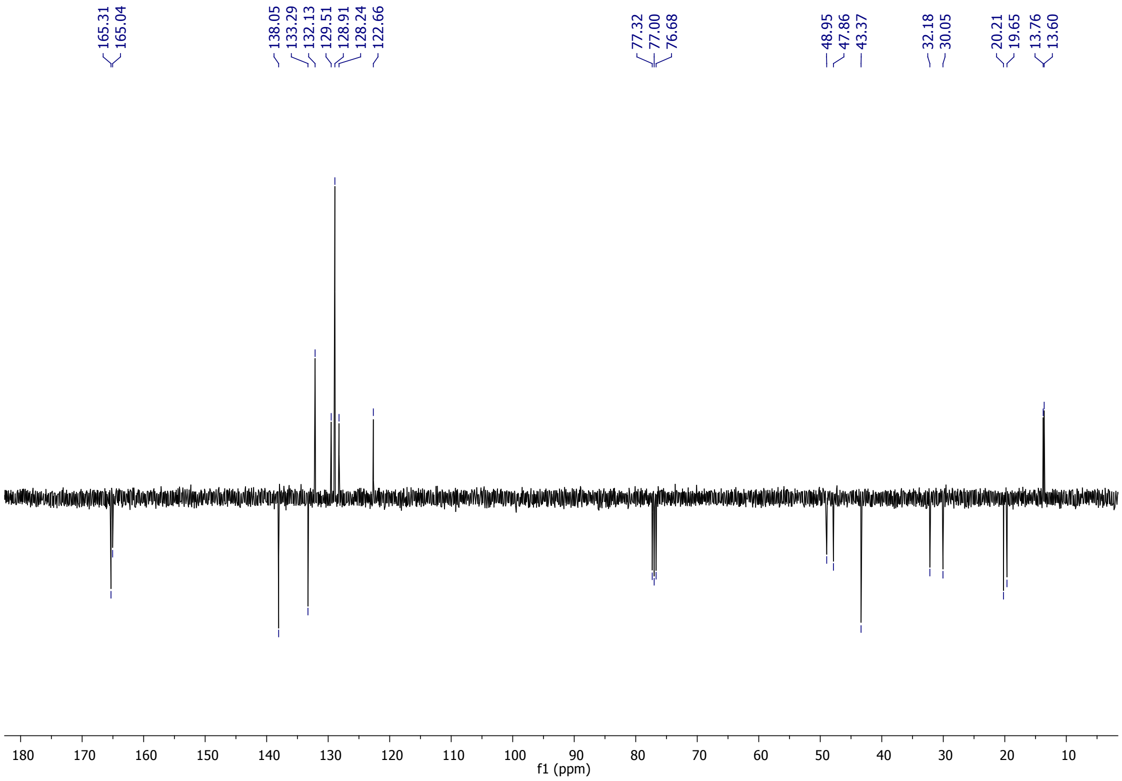

Rotaxane **1** (^{1}H,^{1}H-COSY, 400 MHz, $CDCl_3$, 298 K)

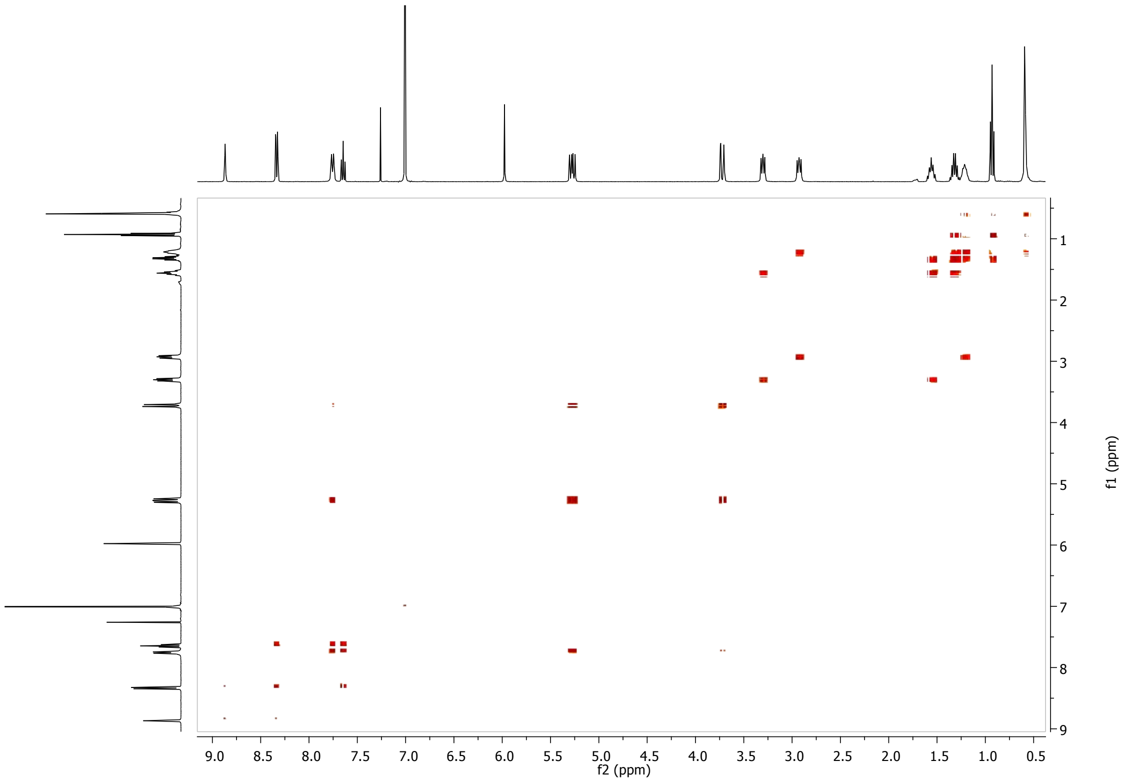

Rotaxane **1** (^{1}H,^{1}H-COSY, 400 MHz, $CDCl_3$, 298 K)

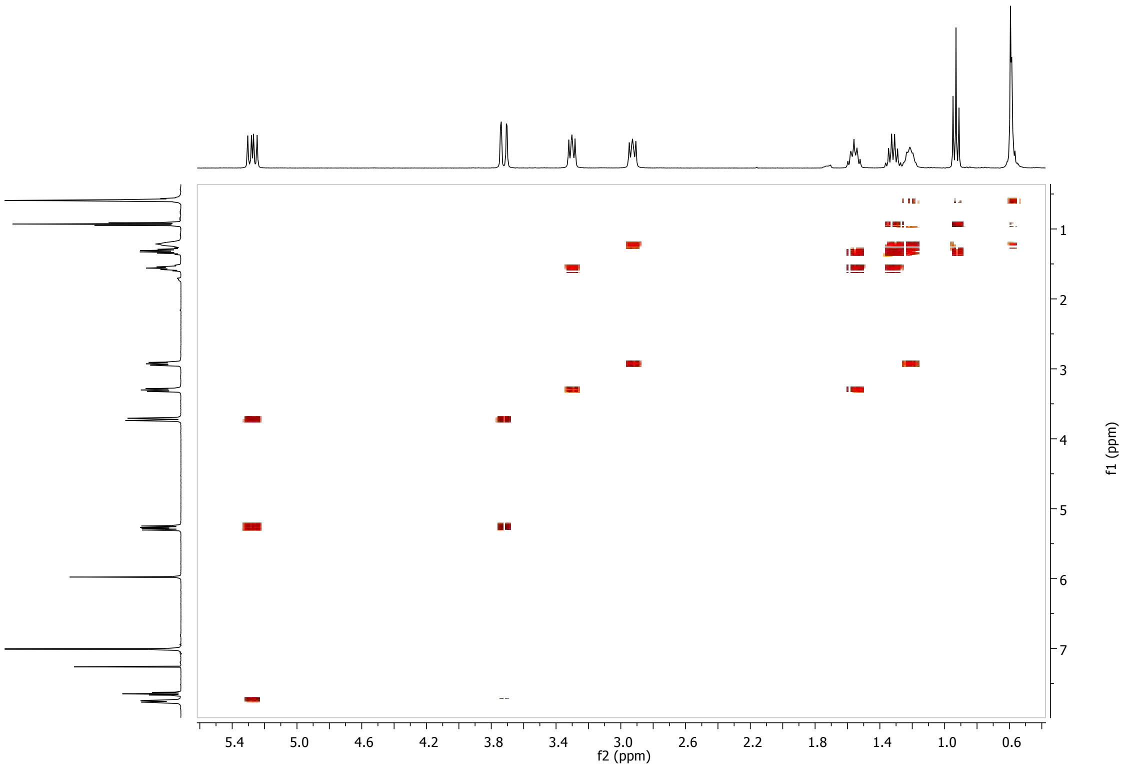

Rotaxane **1** (^{1}H,^{1}H-COSY, 400 MHz, $CDCl_3$, 298 K)

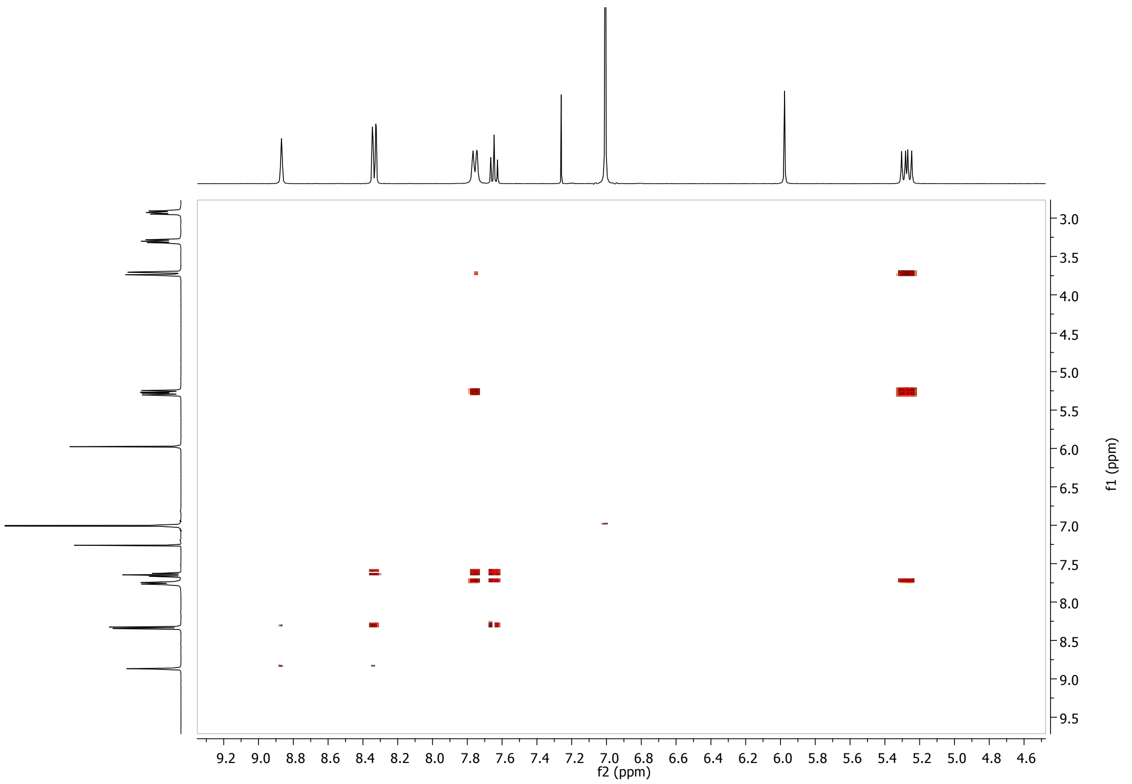

Rotaxane **1** (^{1}H,^{1}H-NOESY, 400 MHz, $CDCl_3$, 298 K)

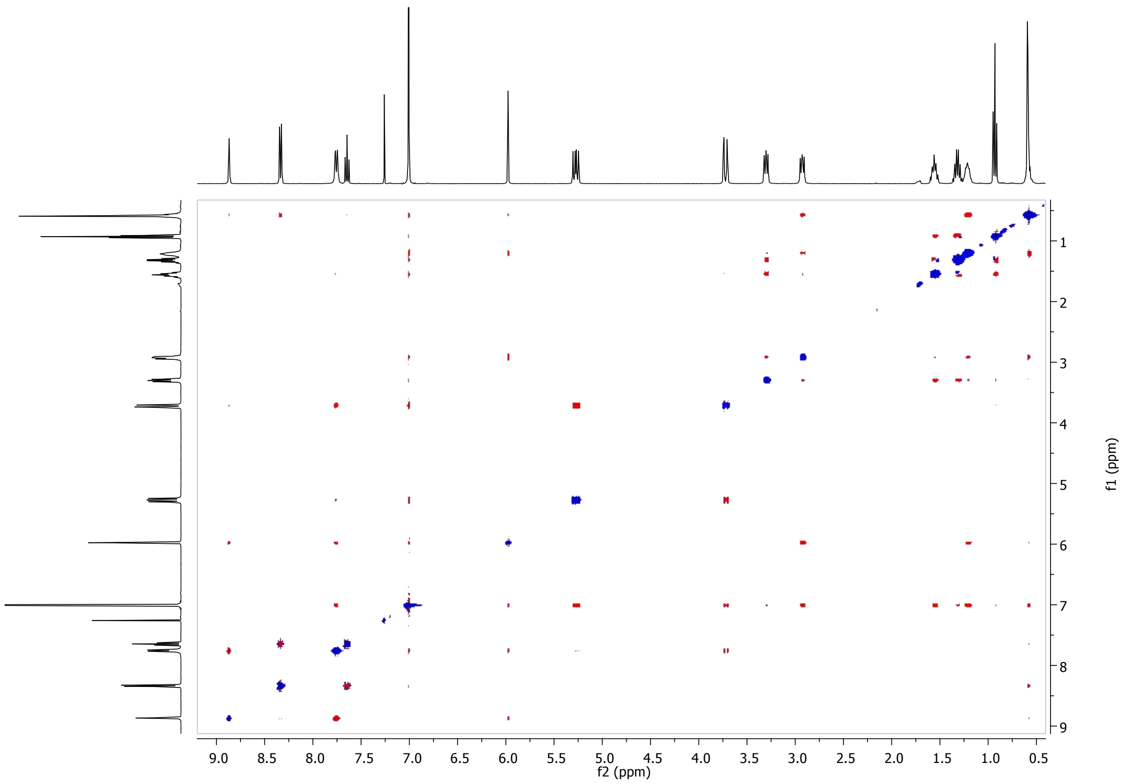

Rotaxane **1** (^{1}H,^{1}H-NOESY, 400 MHz, $CDCl_3$, 298 K)

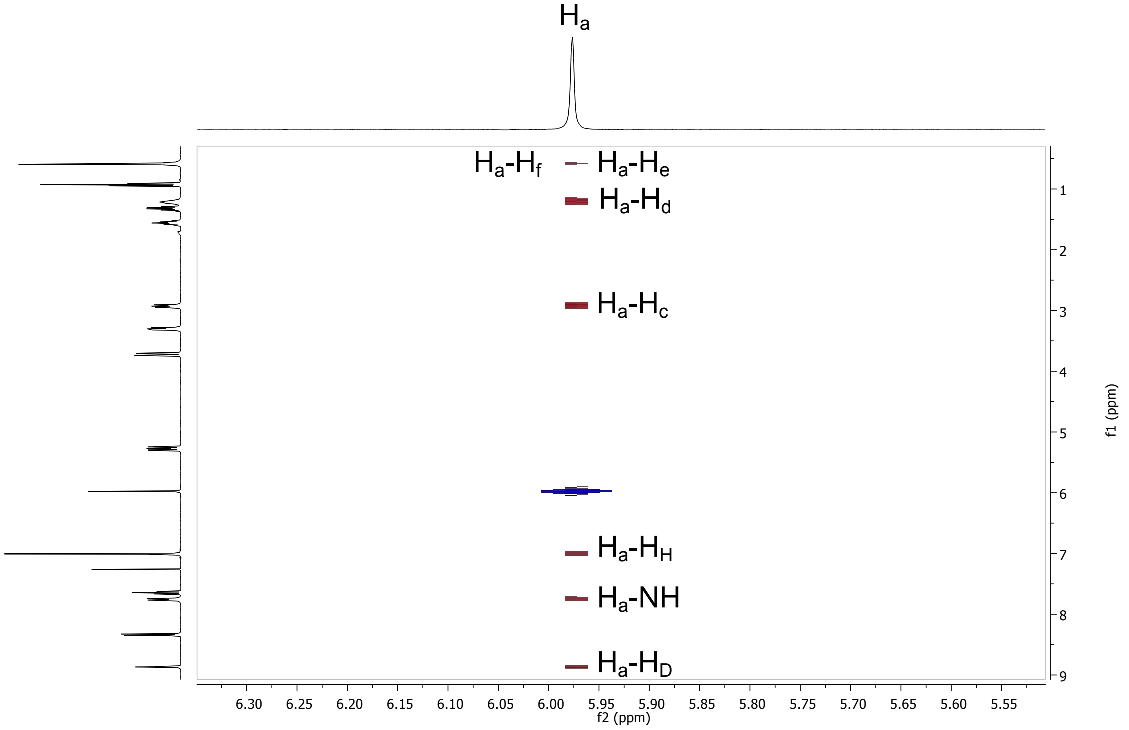

Rotaxane **1** (^{1}H,^{1}H-NOESY, 400 MHz, $CDCl_3$, 298 K)

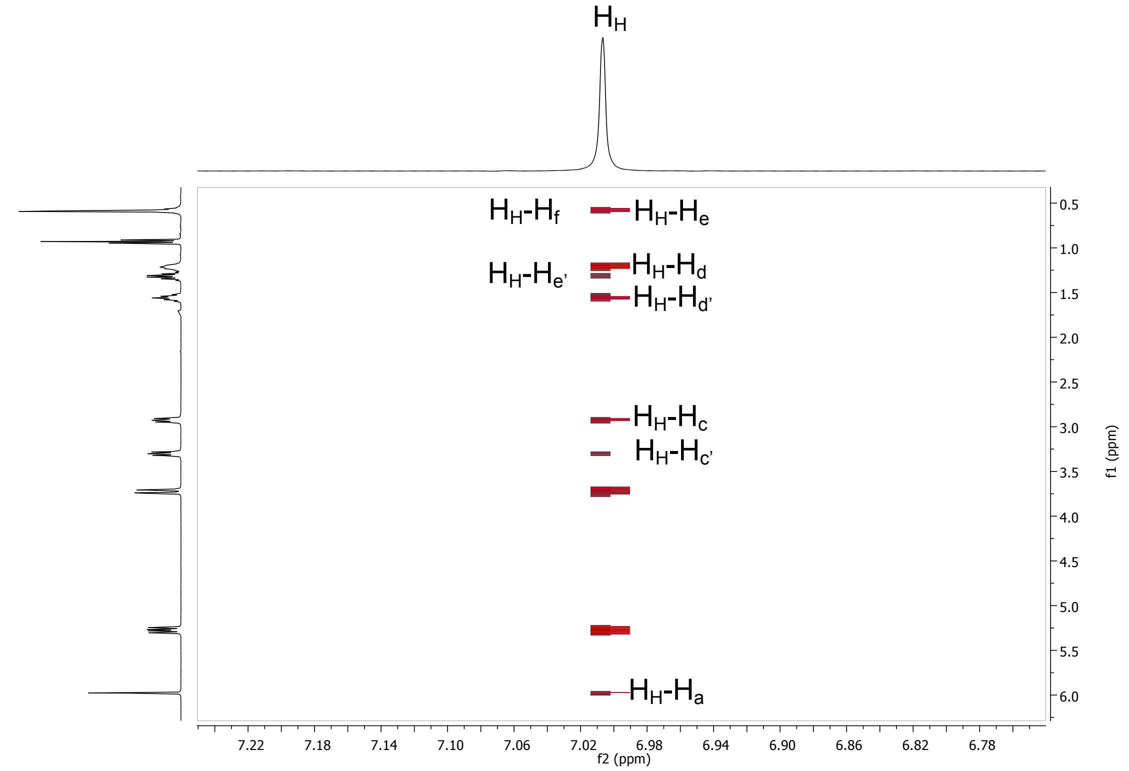

Rotaxane **1** (Selective 1D-NOESY, 400 MHz, $CDCl_3$, 298 K)

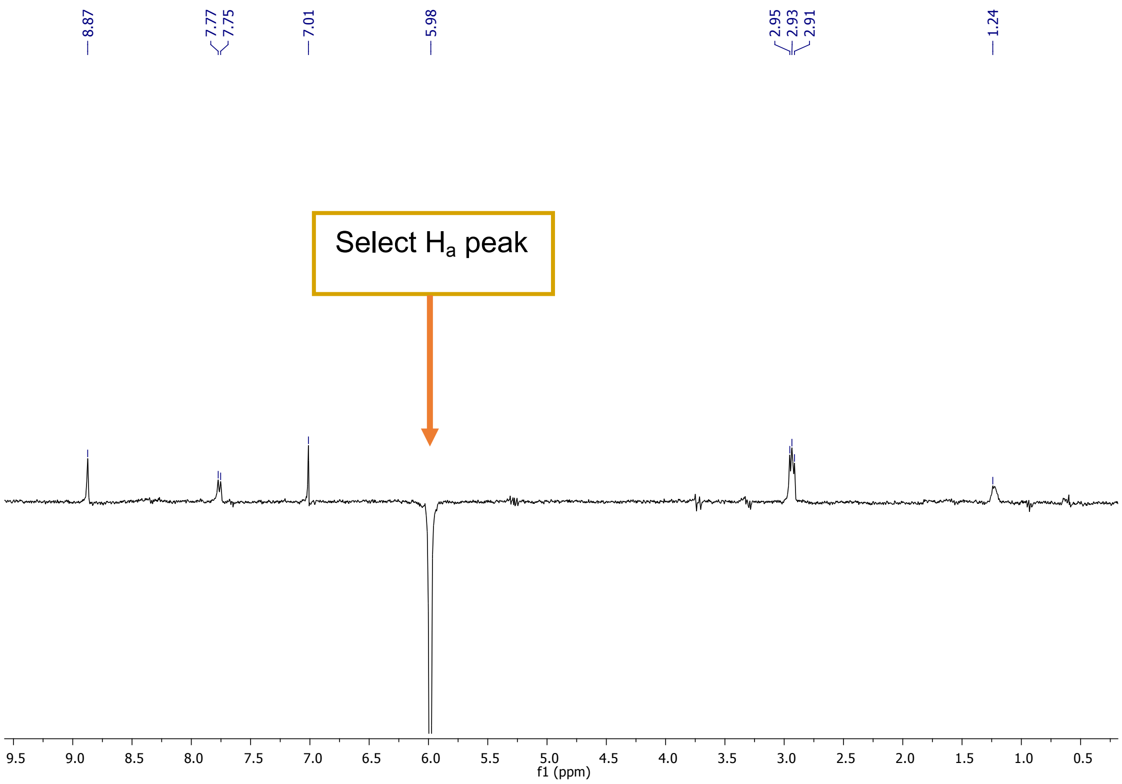

Rotaxane **1** (HSQC, 400 MHz, $CDCl_3$, 298 K)

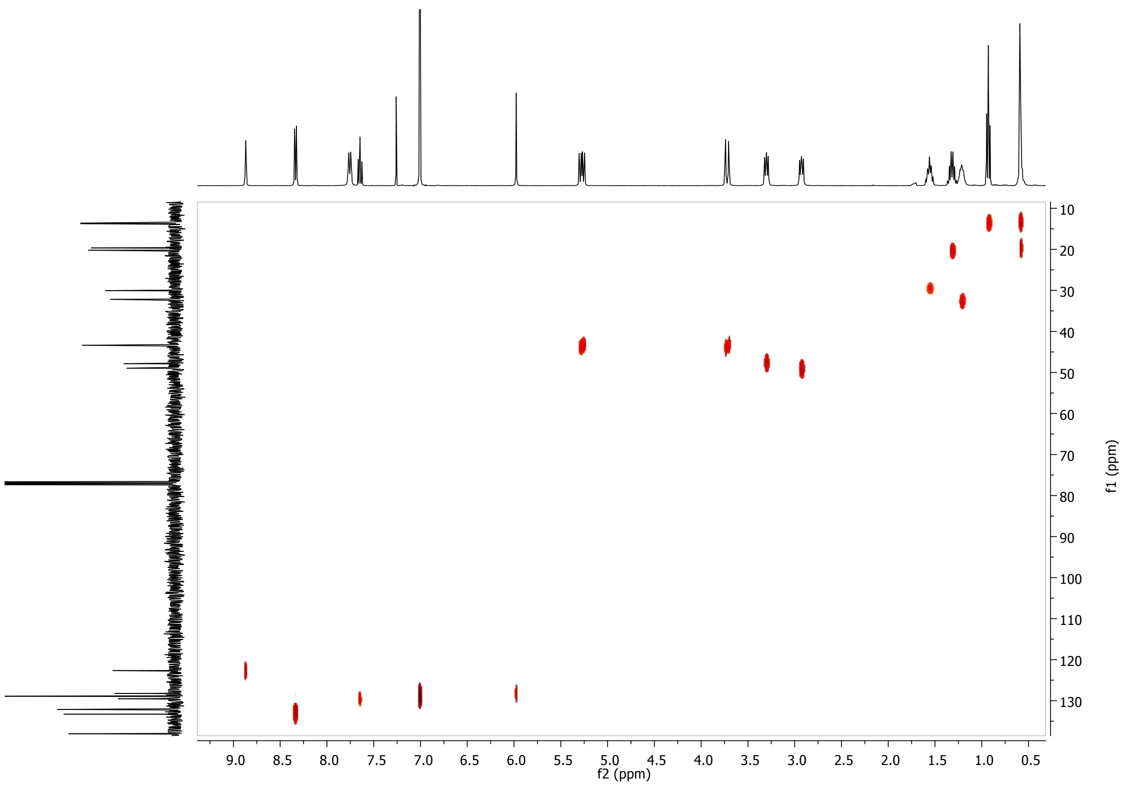

Rotaxane **1** (HSQC, 400 MHz, $CDCl_3$, 298 K)

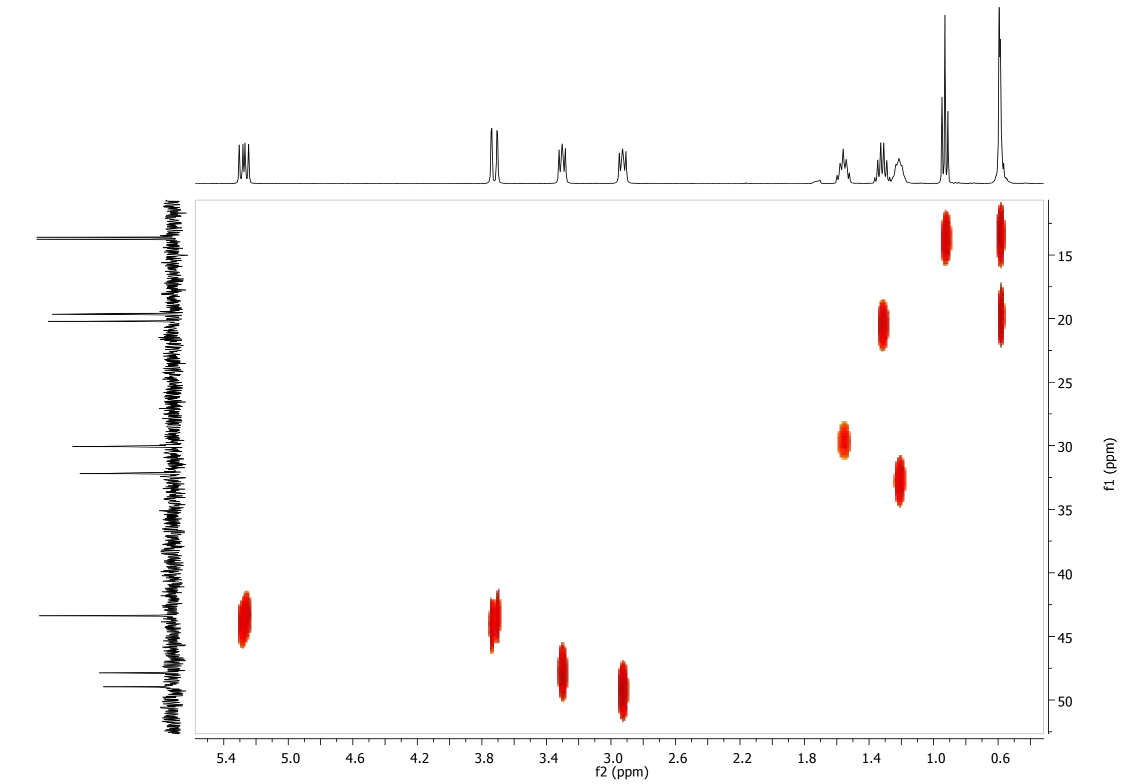

Rotaxane **1** (HSQC, 400 MHz, $CDCl_3$, 298 K)

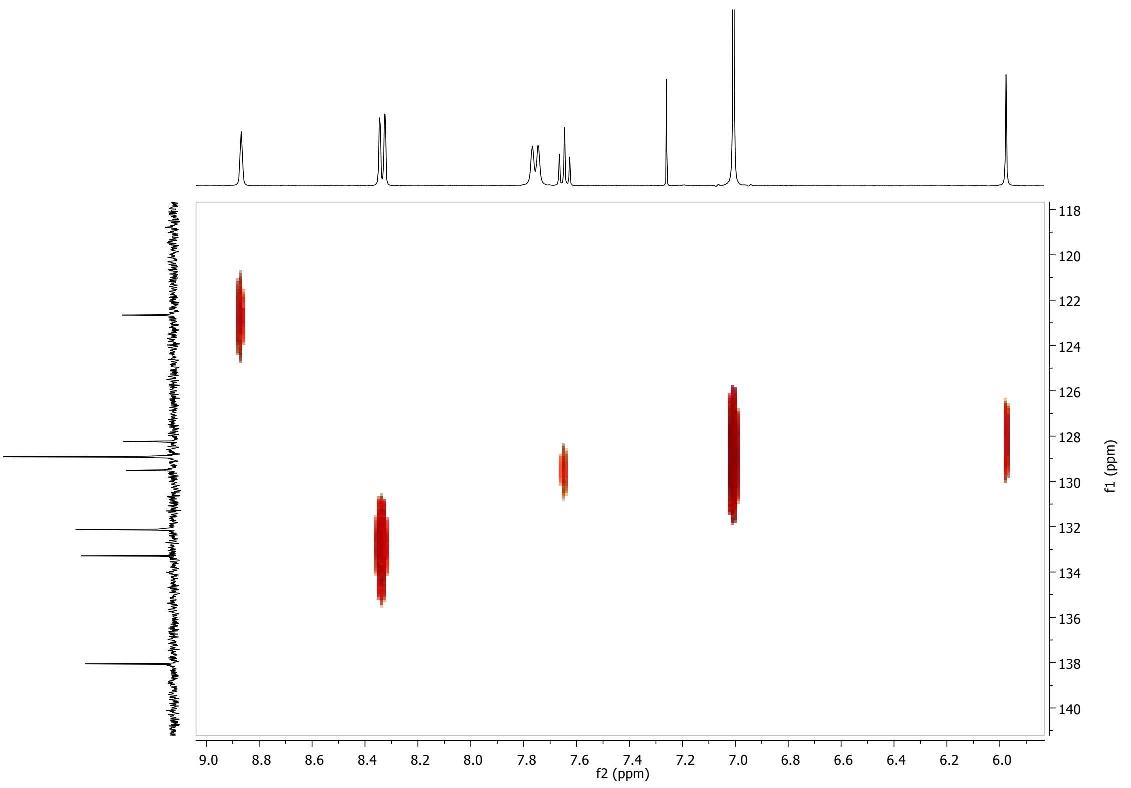

Rotaxane **1** (HMBC, 400 MHz, $CDCl_3$, 298 K)

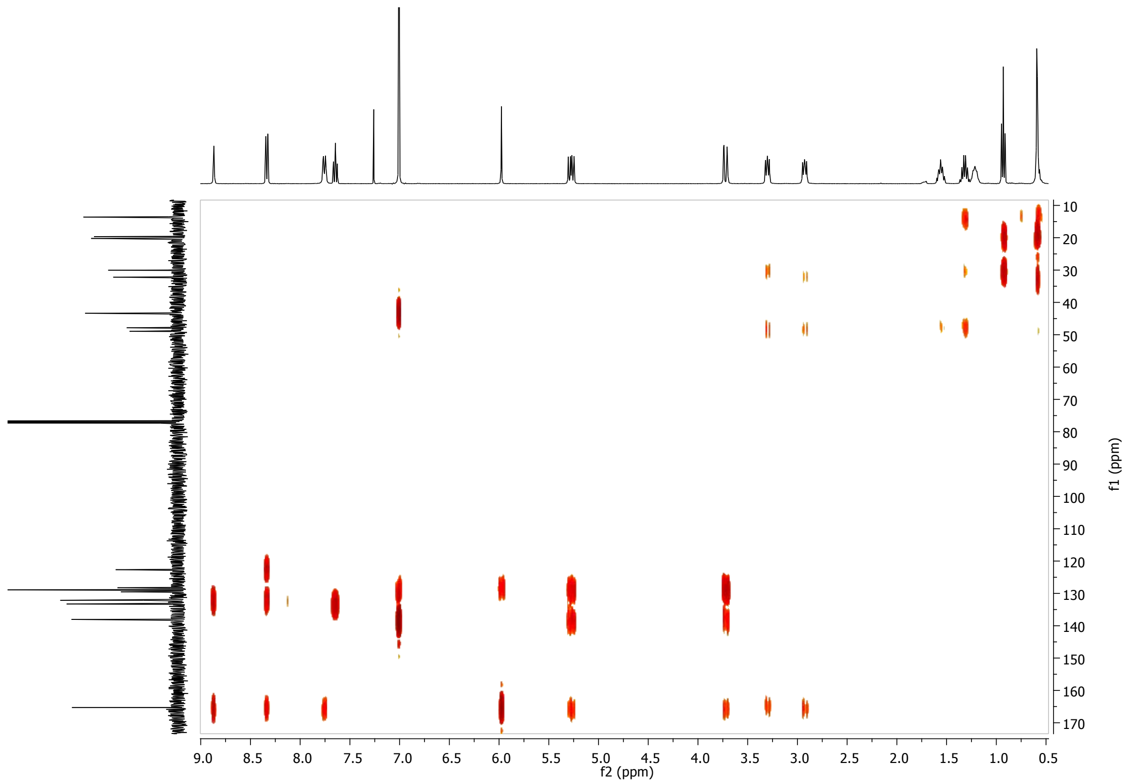

Rotaxane **1** (HMBC, 400 MHz, $CDCl_3$, 298 K)

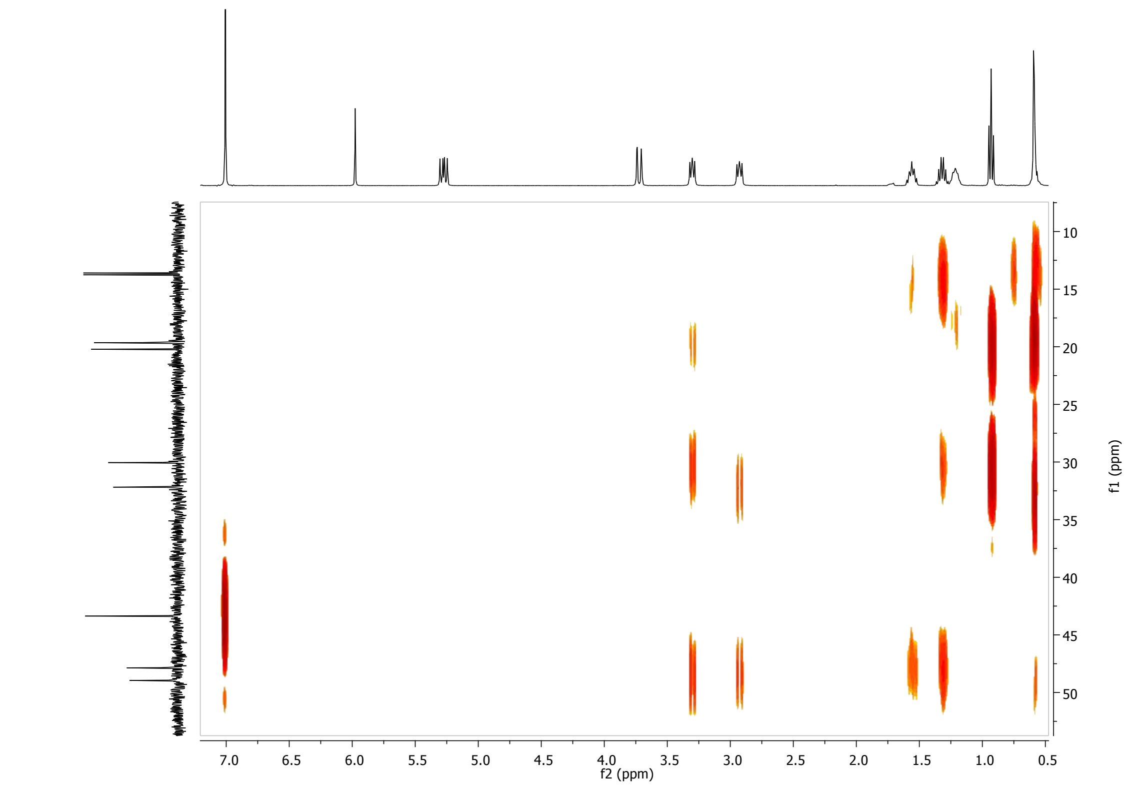

Rotaxane **1** (HMBC, 400 MHz, $CDCl_3$, 298 K)

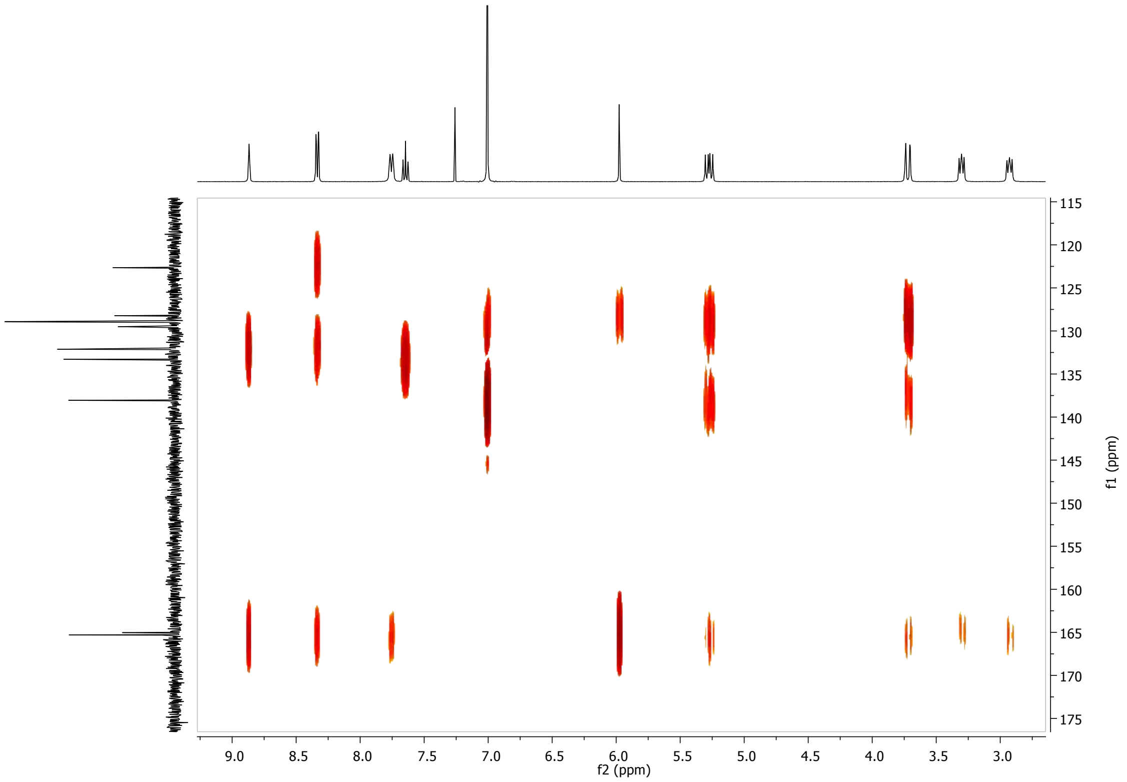

Supplement: Supplementary file 3 — ed3c00163_si_003.pdf [file ed3c00163_si_003.pdf]
