# Supplementary material for: Exploring the Chemistry of the Mechanical Bond: Synthesis of a [2]Rotaxane through Multicomponent Reactions
Source: J Chem Educ. 2023 Aug 1;100(9):3355–63. doi: 10.1021/acs.jchemed.3c00163 (PMC10501439; doi:10.1021/acs.jchemed.3c00163)
Supplement: Supplementary file 8 — ed3c00163_si_008.pdf [file ed3c00163_si_008.pdf]

Exploring the Chemistry of the Mechanical Bond: Synthesis of a [2]Rotaxane through Multicomponent Reactions

Adrian Saura-Sanmartin,* Jorge Lopez-Sanchez, Carmen Lopez-Leonardo, Aurelia Pastor and Jose Berna*

Departamento de Química Orgánica, Facultad de Química, Regional Campus of International Excellence "Campus Mare Nostrum", Universidad de Murcia, E-30100, Murcia, Spain. E-mail: adrian.saura@um.es, ppberna@um.es

Supporting Information Student Handout modified

COMMENT: This document includes the modifications of the student handout. The new additions are in blue, while removed content is struck out.

1 Aims

- To introduce and revise the usual methods for the synthesis of mechanically interlocked molecules and particularly of rotaxanes.
- To synthesize a 2[rotaxane] through a five or three-components clipping reaction.
- To learn key organic chemistry techniques.
- To consolidate previously acquired principles of IR and MNR spectroscopy and to apply it in the characterization of the molecules synthetized as part of the project.

2 Learning Outcomes

At the end of these sessions, you should be able to:

- Extract the relevant information from the bibliography provided in this project.
- Learn experimental techniques employed in laboratories of organic chemistry.
- Learn and develop skills regarding the performance of a research project.
- Perform simple chemical reactions and explain the results in terms of the structure of the compounds obtained.
- Control the purity of the compounds by thin layer chromatography (TLC).
- Increase your knowledge of IR and one-dimensional (^{1}H, ^{13}C, DEPT, APT and 1D-NOESY) and two-dimensional (^{1}H-^{1}H-COSY, ^{1}H,^{1}H-NOESY, ^{1}H,^{13}C-HSQC and ^{1}H,^{13}C-

HMBC) NMR spectroscopic techniques and their application to the characterization of the rotaxane synthesized as part of the project.

- Relate the knowledge acquired with that studied all along the Degree in Chemistry.

3 Schedule

This experience will be carried out in three 3-hour laboratory periods.

This laboratory experiment addresses important concepts of organic chemistry linked to amide chemistry, mechanical bonds, melting point, FT-IR and NMR techniques.

4 Background[i]

[i] This text has been rewritten based on the introduction of the Doctoral Thesis entitled 'Benzylic amide rotaxanes: study of the switching of the motion of their components and their organization in metal-organic frameworks' (Spanish title: 'Rotaxanos de amidas bencílicas: studio de la conmutación del movimiento de sus componentes y su organización en sistemas metal-orgánicos') defended by Adrian Saura-Sanmartin on February 2021 at the Universidad de Murcia (http://hdl.handle.net/10201/104842). The author of the Thesis, a coauthor of this article, has consented to its use.

Mechanically interlocked molecules (MIMs) are a type of compounds constituted by at least two submolecular components.[1,2] These components are not covalently linked, but they are topologically interlocked one to each other. This particular link between the subcomponents of these molecules is known as the mechanical bond.[3]

In order to form a mechanical bond, it is necessary to arrange the different components in an orthogonal geometry. For this purpose, different methods for the assembly of MIMs have been developed over the years. The assembly employing transition metals as templates allows this union to be established by coordination of both components with a metal ion. Through the hydrophobic effect, several components can be joined by forming a hydrophobic cavity. The host-guest interactions between π-donors and acceptors allow the interconnection by the attraction of the components. Another particularly useful approach for obtaining MIMs is the establishment of hydrogen bond interactions between both components.[4,5,6,7,8]

Hydrogen bonds are characterized by their directionality and ability to act cooperatively, forming associations between donor and acceptor groups (Figure S1). In this type of bond, the donor group, the hydrogen atom, and the acceptor one (usually a heteroatom) are aligned. These bonds have a high dipolar character.[9,10]

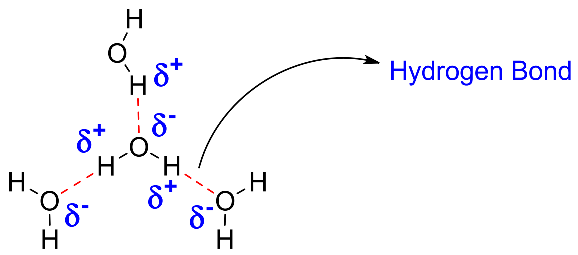

Figure S1. *Water molecules associated by hydrogen bonds. Reprinted with permission of the author from http://hdl.handle.net/10201/104842.*

Within the wide range of synthetic MIMs, two families must be highlighted due to their versatility and their extraordinary properties: catenanes and rotaxanes (Figure S2). Catenanes are molecules in which at least two macrocycles are interlocked, mimicking the chain links.[11] In the particular case of rotaxanes, the two components are of different type, one linear and one cyclic. The simplest example of a rotaxane could be considered as a dumbbell, a linear component with bulky groups at the ends, surrounded by a cyclic one. The bulky groups are known as stoppers and their purpose is to prevent the dethreading of the components.[12] One would think that, to achieve the synthesis of a rotaxane, it is necessary to know how to sew molecules. The linear component, better known as axis or thread, should be introduced through the cyclic component or macrocycle.

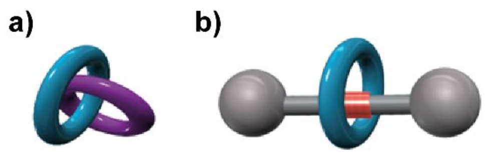

Figure S2. *Cartoon representation of a: (a) catenane; and (b)rotaxane. Reprinted with permission of the author from http://hdl.handle.net/10201/104842.*

The first rotaxane was synthesized by Harrison and Harrison more than five decades ago via a statistical approach (Figure S3a).[13] This type of protocol is characterized by an absence of interactions between the precursors to allow their efficient orientation, affording the interlocked species in very low yields. Later, Professor Schill used a covalent-bond-directed synthesis, which involved the formation of a reversible covalent bond between linear and cyclic components, to obtain another rotaxane (Figure S3b). Although the product was obtained in a higher yield, the synthetic route involved a high number of reaction steps.[14]

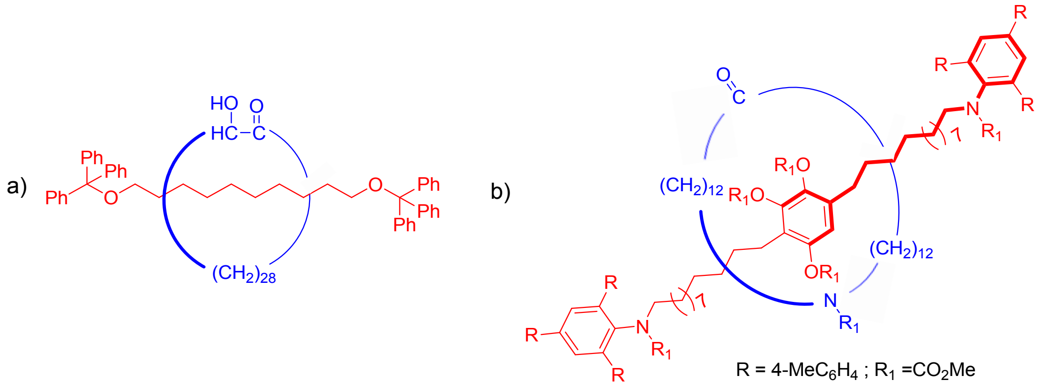

Figure S3. *Structure of (a) a [2]rotaxane prepared by a statistical synthesis [13] and (b) a [2]rotaxane prepared by a covalent bond-directed synthesis.[14] Reprinted with permission of the author from http://hdl.handle.net/10201/104842.*

Since the publication of these early examples, the synthesis of rotaxanes has undergone a remarkable development characterized by the variety of available methods and a significant increase in reaction yields. The most advantageous synthetic methods employ a template that appropriately orients the precursors in space. These methods involve the prior formation of a supramolecular complex stabilized by non-covalent interactions.[15] Subsequent covalent modifications prevent the dissociation of these components.

Template-based methods for the obtention of rotaxanes can be classified into five main types (Figure S4). The capping methodology (Figure S4a), which involves the prior formation of a pseudorotaxane and its subsequent capping. In the snapping methodology

(Figure S4b), once a semirotaxane has been formed, the capping of the non-stopper end is accomplished. In the slipping methodology (Figure S4c), the cyclic component is threaded onto a thread having stoppers under high temperature conditions. The clipping methodology (Figure S4d) involves the cyclisation of a ligand around a thread having stoppers. In the metal-active template method (Figure S4e), the metal plays a dual role, orienting the ligands in the appropriate geometry and catalyzing the formation of the covalent bond that captures the interlocked species.

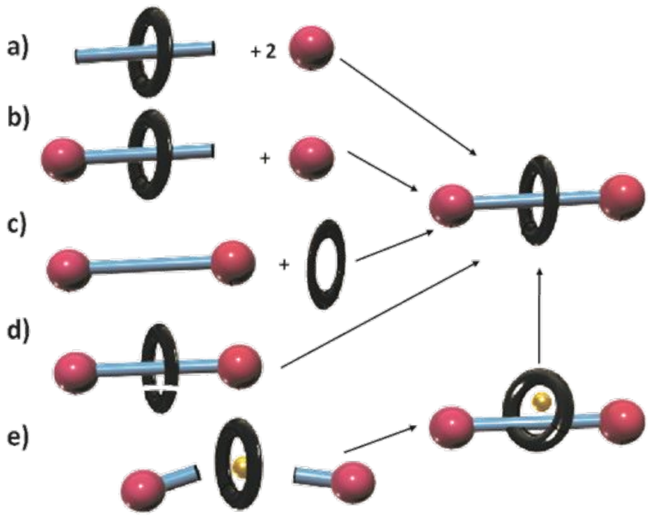

Figure S4. *Main methods for the synthesis of [2]rotaxanes: (a) capping, (b) snapping, (c) slipping, (d) clipping and (e) active metal templating.*

The template syntheses are based on the preorganization of different molecules to form a supramolecular complex by introducing a guest molecule that can establish interactions with a host molecule. One well-known strategy using template synthesis is that based on metal ion chelates which act as templates. Thus, the synthesis of crown ether macrocycles is a really instructive example. In the Figure S5, first a supramolecular complex is form by wrapping the linear ligand around the potassium ion. A subsequent ring closing reaction leads to the target crown ether.[16]

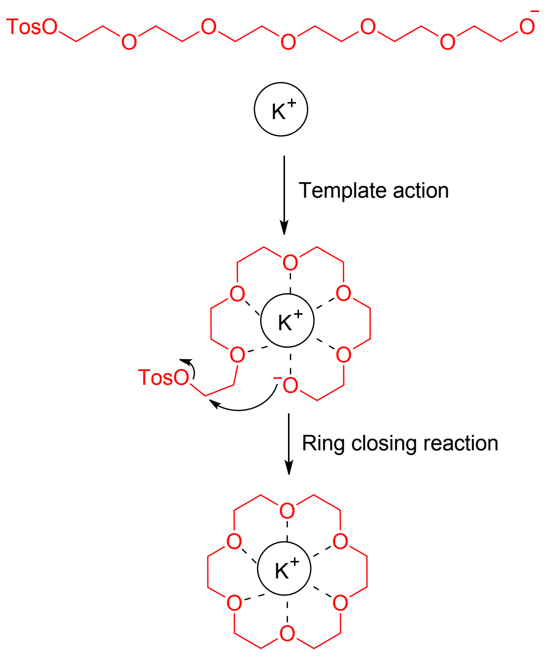

Figure S5. *Template synthesis of a crown ether.*

Rotaxanes are the most interesting type of MIMs because of their greater variety of motions and a large number of applications.[13]

In Nature, MIMs play a fundamental role in many processes occurring within the organism. The establishment of mechanical bonds is critical for the development of various biological processes, such as mitochondrial scission, selective ion transport or DNA replication, which proceed through mechanically interlocked intermediates.[1,17,18,19,20] Nature is always ahead of Science. Whatever a scientist thinks of designing, Nature did it previously. This is why Nature is a great source of inspiration, in particular a wide number of biological systems. There is a duality that may seem contradictory. In order to understand Nature, it is necessary the progress in Science and, for the improvement in research, it is essential to interpret Nature. Thus, chemists have dedicated a lot of effort to understand the chemistry of the mechanical bond, thus overcoming the inherent difficulty in achieving the adequate spatial arrangement of the different components.

5 Safety and Hazards

Chemistry laboratories are potentially dangerous places and therefore must be treated with respect. The following rules are for the safety of you, other students and members of staff and should be followed at all times.

- You should wear appropriate personal protective equipment, such as disposable gloves, goggles, closed shoes and a lab coat.

- The procedures must be performed in a fume hood or similarly ventilated workspace.

- Make sure that all glassware containing chemicals is properly labelled.

- Liquid and solid waste must be disposed into sealed and appropriately labeled containers.

- The rinsed syringe should be disposed into a dedicated, appropriately labelled disposal container. The rinsed needle can be reused after being dried in an oven.

- Report all accidents or near accidents to the lecturer in charge of the class.

- Do not remove samples or chemicals from the laboratory.

Safety information for all reagents is available via the appropriate Safety Data Sheet (SDS).

The Chemical Abstracts Service (CAS) Numbers and Globally Harmonised System (GHS) Hazards of the chemical compounds and solvents used in this work are listed below:

Table S1. Hazards of the chemical compounds employed in the laboratory experiment.

Chemical Compound	CAS Number	GHS Hazards
Celite	68855-54-9	H372: Causes damage to organs (Lungs) through prolonged or repeated exposure if inhaled.
Hydrochloric acid	7647-01-0	H314: Causes severe skin burns and eye damage.<br>H331: Toxic if inhaled.
Isophthaloyl dichloride	99-63-8	H312: Harmful in contact with skin.<br>H314: Causes severe skin burns and eye damage.<br>H318: Causes serious eye damage.<br>H331: Toxic if inhaled.

Magnesium sulphate anhydrous	7487-88-9	H302: Harmful if swallowed.<br>H312: Harmful in contact with skin.<br>H332: Harmful if inhaled.
Sodium chloride	7647-14-5	H319: Causes serious eye irritation.
Sodium hydroxide	1310-73-2	H314: Causes severe skin burns and eye damage.
Triethylamine	121-44-8	H225: Highly flammable liquid and vapor.<br>H302: Harmful if swallowed.<br>H312: Harmful in contact with skin.<br>H314: Causes severe skin burns and eye damage.<br>H332: Harmful if inhaled.
p-Xylylenediamine	539-48-0	H314: Causes severe skin burns and eye damage

Table S2. Hazards of the solvents employed in the laboratory experiment.

Solvent	CAS Number	GHS Hazards
Acetone	67-64-1	H225: Highly Flammable liquid and vapor.<br>H319: Causes serious eye irritation.<br>H336: May cause drowsiness or dizziness.
Chloroform	67-66-3	H302: Harmful if swallowed.<br>H315: Causes skin irritation.<br>H319: Causes serious eye irritation.<br>H331: Toxic if inhaled.

		H351: Suspected of causing cancer.<br><br>H361d: May damage the unborn child.<br><br>H372: Causes damage to organs through prolonged or repeated exposure.
Chloroform-*d*	865-49-6	H302: Harmful if swallowed.<br><br>H315: Causes skin irritation.<br><br>H319: Causes serious eye irritation.<br><br>H331: Toxic if inhaled.<br><br>H336: May cause drowsiness or dizziness.<br><br>H351: Suspected of causing cancer.<br><br>H361: Suspected of damaging fertility or the unborn child.<br><br>H372: Causes damage to organs through prolonged or repeated exposure.<br><br>H373: Causes damage to organs through prolonged or repeated exposure.
Diethyl ether	60-29-7	H224 Extremely flammable liquid and vapour<br>H302 Harmful if swallowed<br>H336 May cause drowsiness and dizziness

CAS Registry Numbers (often referred to as CAS RN® or CAS Numbers) are universally used to provide a unique, unmistakable identifier for chemical substances.

The Globally Harmonised System (GHS) is a single worldwide system for classifying and communicating the hazardous properties of industrial and consumer chemicals.

6 General Experimental Information

All commercially available compounds were purchased from Merck, Acros Organics, or Alfa-Aesar Chemical Co. and used without purification. *N*^{1},*N*^{1},*N*^{4},*N*^{4}-Tetrabutylfumaramide (**2**) was prepared from fumaroyl dichloride and dibutylamine[21] and the pre-formed U-shaped component. *N*^{1},*N*^{3}-Bis[4-(aminomethyl)benzyl]isophthalamide (**5**), was generated in situ by the acidic deprotection of the corresponding di-Boc derivative,[22] following the described procedures. HPLC grade solvents (Scharlab) were nitrogen-saturated as well dried and deoxygenated using an Innovative Technology Inc. Pure-Solv 400 Solvent Purification System. Deionized water is used in the preparation of all aqueous solutions. Brine refers to a saturated aqueous solution of sodium chloride.

TLC chromatographies are performed on precoated silica gel on aluminum cards (0.25 mm thick, with 254 nm fluorescent indicator, Fluka) and observed under UV light (254 or 365 nm).

The **additions** are made with *kd* Scientific motor-driven syringe pumps (model number 101) and stainless steel 304 syringe needle, noncoring point (30 cm) from Sigma-Aldrich.

Under reduced pressure refers to the use of a Büchi Rotavapor R-3000 or a Heidolph Hei-Vap Value G3 apparatus with a Vacuumbrand CVC 3000 vacuum pump equipped with a water bath for aiding to remove the solvent.

Melting point (m.p.) is determined on a Kofler hot-plate melting point apparatus and is uncorrected.

Infrared spectroscopy is performed using a PerkinElmer Spectrum 65, FT-IR Spectrometer (ATR) in the range 4000-600 cm^{-1}. The intensities of the absorption bands are indicated as vs (very strong), s (strong), m (middle) and w (weak).

NMR spectra are recorded at 298 K using a Bruker Avance 400 instrument (400 MHz ^{1}H frequency, 101 MHz ^{13}C frequency). Chemical shifts are quoted in parts per million (ppm), referenced to residual chloroform (7.26 ppm for ^{1}H NMR, 77.00 ppm for ^{13}C NMR), as internal standard, whereas coupling constants, *J*, are quoted in Hz. Multiplicities are quoted as s (singlet), d (doublet), dd (doublet of doublet), t (triplet) and m (multiplet).

Signals in the ^1H and ^{13}C NMR spectra of the synthesized compounds are assigned with the aid of DEPT, APT, or two-dimensional NMR experiments (COSY, HMQC, HMBC and NOESY). All NMR data are processed using the MestReNova software (from MestreLab Research S. L.).

7 Experimental Procedure for the five-component synthesis of [2]rotaxane 1[23]

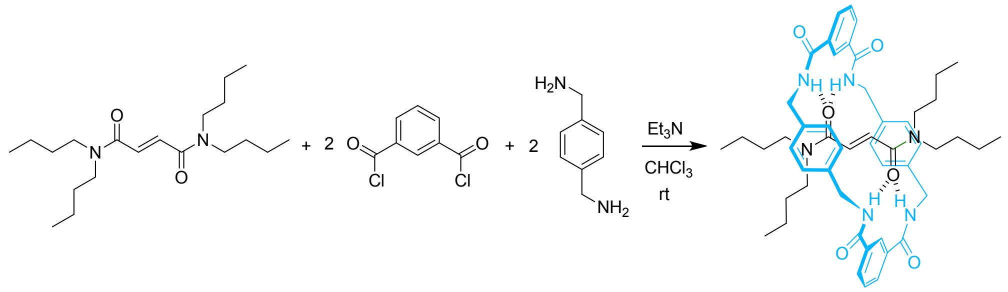

2 3 4 1

Protocol:

The amounts of each reagent and solvent used, as well as the addition time for each pair of students are specified in Table S1.

1. $CHCl_3$ (n - 40 mL) is added to a round bottom flask (RBF) equipped with a stir bar.
2. The thread **2** (x equiv) and Et_3N (z/2 equiv) were added to the RBF.
3. The RBF is sealed with a septum and the solution is stirred vigorously.
4. Two 25 mL syringes are prepared with the needles previously dried in the oven or alternatively, two dried dropping funnels.
5. The first syringe or dropping funnel is filled with a solution of isophthaloyl dichloride (**3**) (y equiv) in $CHCl_3$ (20 mL).
6. A solution of *p*-xylylenediamine (**4**) (y equiv) and Et_3N (z/2 equiv) in $CHCl_3$ (20 mL) is loaded into a second syringe or dropping funnel.
7. The two syringes are adapted to the motor-driven syringe pump and programmed for the assigned addition time according to table S1. When dropping funnels are used instead of syringes, the addition rate should be controlled manually.

8. After completion of the addition, the reaction mixture is stirred for additional 10 minutes.
9. The resulting suspension is filtered through a Celite pad.
10. The filtrate is sequentially washed with water (2 x 50 mL), a solution of HCl 1 M (2 × 50 mL), a saturated solution of NaOH 1 M (2 × 50 mL) and brine (2 × 50 mL).
11. The organic phase was then dried with anhydrous $MgSO_4$.
12. The solution is filtered and the solvent was removed under reduced pressure.
13. The resulting solid is filtered and washed with diethyl ether (3 x 15 mL) until all the unreacted thread **2** is extracted. To identify the product on TLC it is a common practice to run a side-by-side comparison of all material potentially present in the crude mixture. Make a TLC (SiO_2, $CHCl_3$/acetone 9:1) after each extraction with 5 mL of ether, comparing with a pure sample of fumaramide **2**.
14. The solid [2]rotaxane **1** is dried under vacuum.

Product identification:

1. Measure the melting point of your product and compare it with that described in the literature.
2. Performs an IR-ATR spectrum of your product.
3. Prepare a sample for NMR analysis with 15 mg of your product and $CDCl_3$.
4. With the help of your instructor, perform the following one-dimensional spectra: ^{1}H, ^{13}C and DEPT-135 (or APT) and two-dimensional spectra: $^{1}H,^{1}H$-COSY, $^{1}H,^{1}H$-NOESY, HSQC and HMBC.

8 Experimental Procedure for the three-component synthesis of [2]rotaxane 1

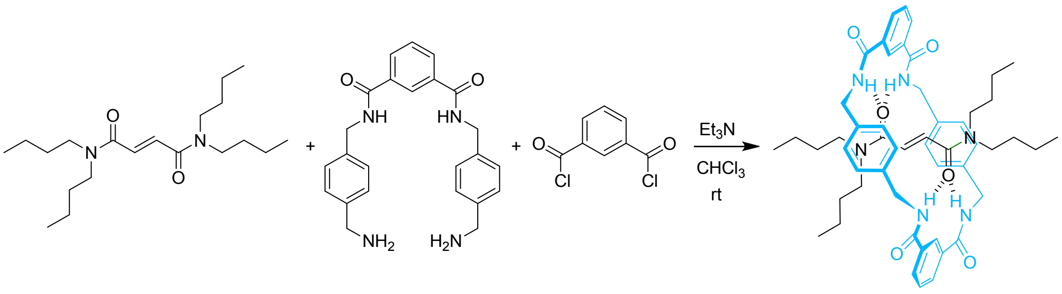

2 5 3 1

Protocol:

The amounts of each reagent and solvent used, as well as the addition time for each pair of students are specified in Table S1.

1. $CHCl_3$ (n - 20 mL) is added to a round bottom flask (RBF) equipped with a stir bar.
2. The thread **2** (x equiv), the pre-formed U-shaped **5** (y equiv) and Et_3N (z equiv) were added to the RBF.
3. The RBF is sealed with a septum and the solution is stirred vigorously.
4. One 25 mL syringe is prepared with the needle previously dried in the oven.
5. The syringe is filled with a solution of isophthaloyl dichloride (**3**) (y equiv) in $CHCl_3$ (20 mL).
6. The syringe is adapted to the motor-driven syringe pump and programmed for the assigned addition time according to table S1.
7. After completion of the addition, the reaction mixture is stirred for additional 10 minutes.
8. The resulting suspension is filtered through a Celite pad.
9. The filtrate is sequentially washed with water (2 x 50 mL), a solution of HCl 1 M (2 × 50 mL), a saturated solution of NaOH 1 M (2 × 50 mL) and brine (2 × 50 mL).
10. The organic phase was then dried with anhydrous $MgSO_4$.
11. The solution is filtered and the solvent was removed under reduced pressure.
12. The resulting solid is filtered and washed with diethyl ether (3 x 15 mL) until all the unreacted thread **2** is extracted. To identify the product on TLC it is a common practice to run a side-by-side comparison of all material potentially present in the

crude mixture. Make a TLC (SiO_2, $CHCl_3$/acetone 9:1) after each extraction with 5 mL of ether, comparing with a pure sample of fumaramide **2**.

13. The solid [2]rotaxane **1** is dried under vacuum.

Product identification:

1. Measure the melting point of your product and compare it with that described in the literature.
2. Performs an IR-ATR spectrum of your product.
3. Prepare a sample for NMR analysis with 15 mg of your product and $CDCl_3$.
4. With the help of your instructor, perform the following one-dimensional spectra: ^1H, ^{13}C and DEPT-135 (or APT) and two-dimensional spectra: $^1H,^1H$-COSY, $^1H,^1H$-NOESY, HSQC and HMBC.

Each student will perform the reaction using different conditions according to Table S1.

Table S3. Different reaction conditions carried out by the students

Entry	Five-Component Reaction[a]	Three-Component Reaction[b]	Solvent (n mL)	Addition Time (min)
1	Conditions A		90	30
2	Conditions A		90	60
3	Conditions A		90	90
4	Conditions A		180	30
5	Conditions A		180	60
6	Conditions A		180	90
7	Conditions A		360	90
~~8~~	~~Conditions B~~		~~90~~	~~90~~
~~9~~	~~Conditions B~~		~~180~~	~~90~~
10		Conditions A	180	90
11		Conditions B	180	90

[a] Conditions A: **2** (1 equiv), **3** (8 equiv), **4** (8 equiv), Et_3N (24 equiv). ~~Conditions B: **2** (8 equiv), **3** (1 equiv), **4** (1 equiv), Et_3N (8 equiv)~~.
[b] Conditions A: **2** (1 equiv), **5** (8 equiv), **3** (8 equiv), Et_3N (24 equiv). Conditions B: **2** (8 equiv), **5** (1 equiv), **3** (1 equiv), Et_3N (8 equiv).

Complete the following tables (S4 and S5) with appropriate values for the reagents to be used. Some information has been provided to guide you.

Table S4. Data for the five-component synthesis of [2]rotaxane **1**

	**2**	**3**	**4**	**Et_3N**
MF	$C_{20}H_{38}N_2O_2$	$C_8H_4Cl_2O_2$	$C_8H_{12}N_2$	$C_6H_{15}N$
MW	338.54	203.02	136.20	101.19
	Conditions A			
equiv	1	8	8	24
mmol	___	___	___	___
g or mL	0.2 g	___ g	___ g	___ g (___ mL)
	Condition B			
equiv	8	1	1	8
mmol	___	___	___	___
g or mL	0.6 g	___ g	___ g	___ g (___ mL)

Table S5. Data for the three-component synthesis of [2]rotaxane **1**

	**2**	**5**	**3**	**Et_3N**
MF	$C_{20}H_{38}N_2O_2$	$C_{24}H_{26}N_4O_2$	$C_8H_4Cl_2O_2$	$C_6H_{15}N$
MW	338,54	402.49	203.02	101,19
	Conditions A			
equiv	1	8	8	24
mmol	___	___	___	___
g or mL	0.2 g	___ g	___ g	___ g (___ mL)

	Conditions B			
equiv	8	1	1	8
mmol	____	____	____	____
g or mL	0.6 g	____ g	____ g	____ g (____ mL)

9 Important questions

1. Definition of the following concepts:

   (a) supramolecular chemistry

   (b) supramolecular complex

   (c) chemical bond

   (d) hydrogen bond

   (e) mechanical bond

   (f) molecular machine

   (g) rotaxane

   (h) template effect

   (i) anisotropy in aromatic rings (NMR)

   (j) Nuclear Overhauser Effect (NOE)

2. Draw the structure of:

   (a) a fumaramide

   (b) a tetralactam macrocycle

3. It is necessary to prepare 100 mL of HCl 1 M from the commercial one. Calculate the amount (mL) you need of the commercial one (37%).

4. Calculate the amount of NaOH you need to weigh in order to obtain 100 mL of a 1 M solution.

5. Assign the ^{1}H NMR spectrum of tetrabutylfumaramide.

N^{1},*N*^{1},*N*^{4},*N*^{4}-tetrabutylfumaramide (^{1}H NMR, 400 MHz, $CDCl_3$, 298 K)

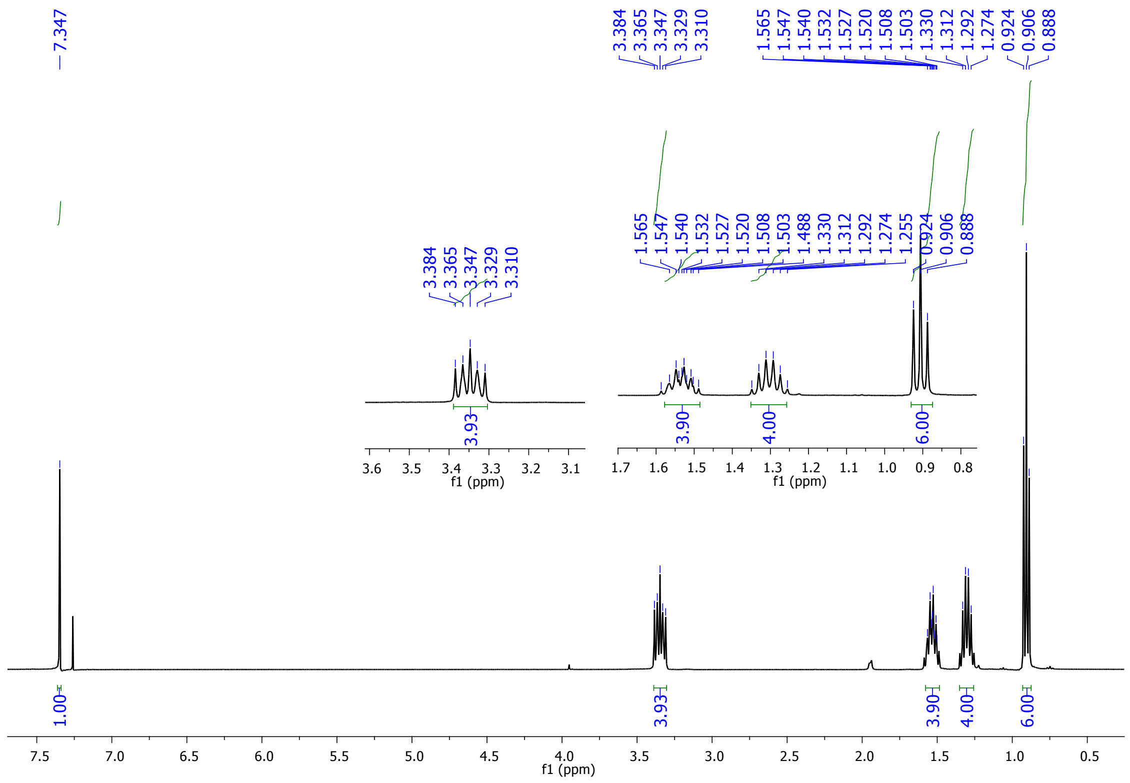

10 References

1 C. J. Bruns, J. F. Stoddart. The Mechanical Bond: A Work of Art. *Top. Curr. Chem.* **2011**, *323*, 19-72.

2. J. E. M. Lewis, M. Galli, S. M. Goldup. Properties and Emerging Applications of Mechanically Interlocked Ligands. *Chem. Commun.* **2017**, *53*, 298-312.

3. E. A. Neal, S. M. Goldup. Chemical Consequences of Mechanical Bonding in Catenanes and Rotaxanes: Isomerism, Modification, Catalysis and Molecular Machines for Synthesis. *Chem. Commun.* **2014**, *50*, 5128-5142.

4. S. Erbas-Cakmak, D. A. Leigh, C. T. McTernan, A. L. Nussbaumer. Artificial Molecular Machines. *Chem. Rev.* **2015**, *115*, 10081-10206.

5. D. B. Amabilino, J. F. Stoddart. Interlocked and Intertwined Structures and Superstructures. *Chem. Rev.* **1995**, *95*, 2725-2828.

6. F. Vögtle, T. Dünnwald, T. Schmidt. Catenanes and Rotaxanes of the Amide Type. *Acc. Chem. Res.* **1996**, *29*, 451-460.

7. J. E. Beves, B. A. Blight, C. J. Campbell, D. A. Leigh, R. T. McBurney. Strategies and Tactics for the Metal-Directed Synthesis of Rotaxanes, Knots, Catenanes and Higher Order Links. *Angew. Chem., Int. Ed.* **2011**, *50*, 9620-9327.

8. N. H. Evans. Recent Advances in the Synthesis and Application of Hydrogen Bond Templated Rotaxanes and Catenanes. *Eur. J. Org. Chem.* **2019**, 3320-3343.

9. L. Pauling. The Structure and Entropy of Ice and of Other Crystals with Some Randomness of Atomic Arrangement. *J. Am. Chem. Soc.* **1935**, *57*, 2680-2684.

10. P. A. Kollman, L. C. Allen. The Theory of the Hydrogen Bond. *Chem. Rev.* **1972**, *72*, 283-303.

11. J. F. Stoddart. The Chemistry of the Mechanical Bond. *Chem. Soc. Rev.* **2009***, 38*, 1802-1820.

12. M. Xue, Y. Yang, X. Chi, X. Yan, F. Huang. Development of Pseudorotaxanes and Rotaxanes: From Synthesis to Stimuli-Responsive Motions to Applications. *Chem. Rev.* **2015**, *115*, 7398-7501.

13. I. T. Harrison, S. Harrison. Synthesis of a stable complex of a macrocycle and a threaded chain. *J. Am. Chem. Soc.* **1967**, *89*, 5723-5724.

14. G. Schill, H. Zollenkopf, Rotaxan-Verbindungen, I. *Justus Liebigs Ann. Chem.* **1969**, *721*, 53-74.

15. F. G. Gatti, D. A. Leigh, S. A. Nepogodiev, A. M. Z. Slawin, S. J. Teat, J. K. Y. Wong. Stiff and Sticky in the Right Places: The Dramatic Influence of Preorganizing Guest Binding Sites on the Hydrogen Bond-Directed Assembly of Rotaxanes. *J. Amer. Chem. Soc.* **2001**, *123*, 5983-5989.

16. R. Hoss, F. Vögtle. Template Syntheses. *Angew. Chem. Int. Ed. Engl.* **1994**, *33*, 375-384.

17. J. A. Mears, L. L. Lackner, S. Fang, E. Ingerman, J. Numari, J. E. Hinshaw. Conformational Changes in Dnm1 Support a Contactile Mechanism for Mitocondrial Fission. *Nat. Struct. Mol. Biol.* **2011**, *18*, 20-26.

18. E. Gouaux, R. MacKinnon. Principles of Selective Ion Transport in Channels and Pumps. *Science* **2005**, *310*, 1461-1465.

19. A. Tonath. Hibernating Bears, Antibiotics and the Evolving Ribosome. *Angew. Chem., Int. Ed.* **2010**, *49*, 4340-4354.

20. K. N. Kreuzer, N. R. Cozzarelli. Formation and Resolution of DNA Catenanes by DNA Gyrase. *Cell* **1980**, *20*, 245-254.

21. C. Lopez-Leonardo, A. Martinez-Cuezva, D. Bautista, M. Alajarin, J. Berna. Homo and heteroassembly of amide-based [2]rotaxanes using α,α'-dimethyl-*p*-xylylenediamines. *Chem. Commun.* **2019**, *55*, 6787-6790.

22. D. Gonzalez Cabrera, B. D. Koivisto, D. A. Leigh. A metal-complex-tolerant CuAAC ‘click’ protocol exemplified through the preparation of homo- and mixed-metal-coordinated [2]rotaxanes. *Chem. Commun.* **2007**, 4218-4220.

23. The conditions were modified based on the previously reported work: A. Martinez-Cuezva, F. Morales, G. R. Marley, A. Lopez-Lopez, J. C. Martinez-Costa, D. Bautista, M. Alajarin, J. Berna. Thermally and Photochemically Induced Dethreading of Fumaramide-Based Kinetically Stable Pseudo[2]rotaxanes. *Eur. J. Org. Chem.* **2019**, 3480-3488.
