# Supplementary material for: Exploring the Chemistry of the Mechanical Bond: Synthesis of a [2]Rotaxane through Multicomponent Reactions
Source: J Chem Educ. 2023 Aug 1;100(9):3355–63. doi: 10.1021/acs.jchemed.3c00163 (PMC10501439; doi:10.1021/acs.jchemed.3c00163)
Supplement: Supplementary file 10 — ed3c00163_si_010.zip [file ed3c00163_si_010.zip › JCE_SI2 Seminar presentation_QOS.pptx]

## Slide 1
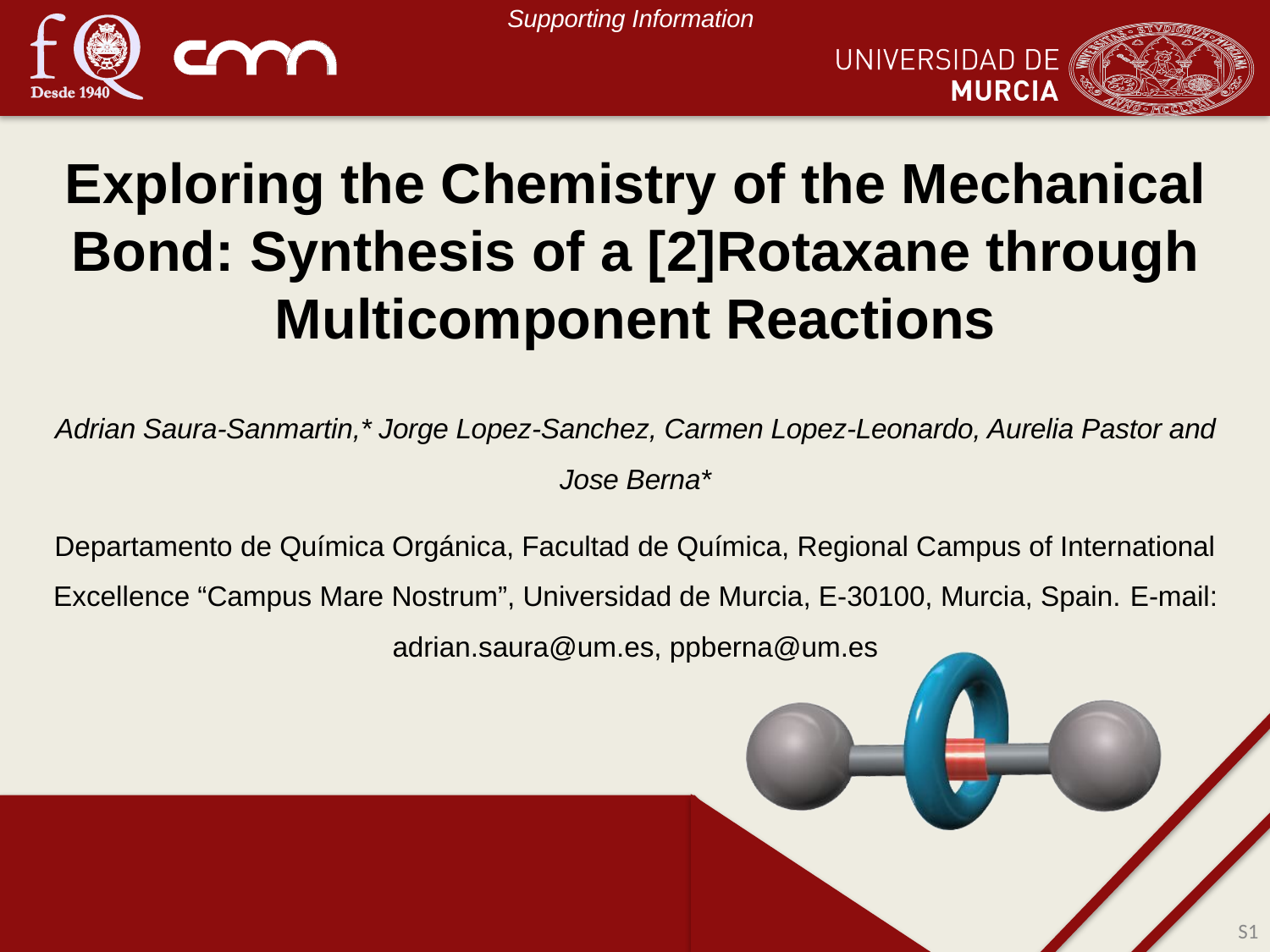

Supporting Information
Exploring the Chemistry of the Mechanical Bond: Synthesis of a [2]Rotaxane through Multicomponent Reactions
Adrian Saura-Sanmartin,* Jorge Lopez-Sanchez, Carmen Lopez-Leonardo, Aurelia Pastor and Jose Berna*
Departamento de Química Orgánica, Facultad de Química, Regional Campus of International Excellence “Campus Mare Nostrum”, Universidad de Murcia, E-30100, Murcia, Spain. E-mail: adrian.saura@um.es, ppberna@um.es
S1

## Slide 2
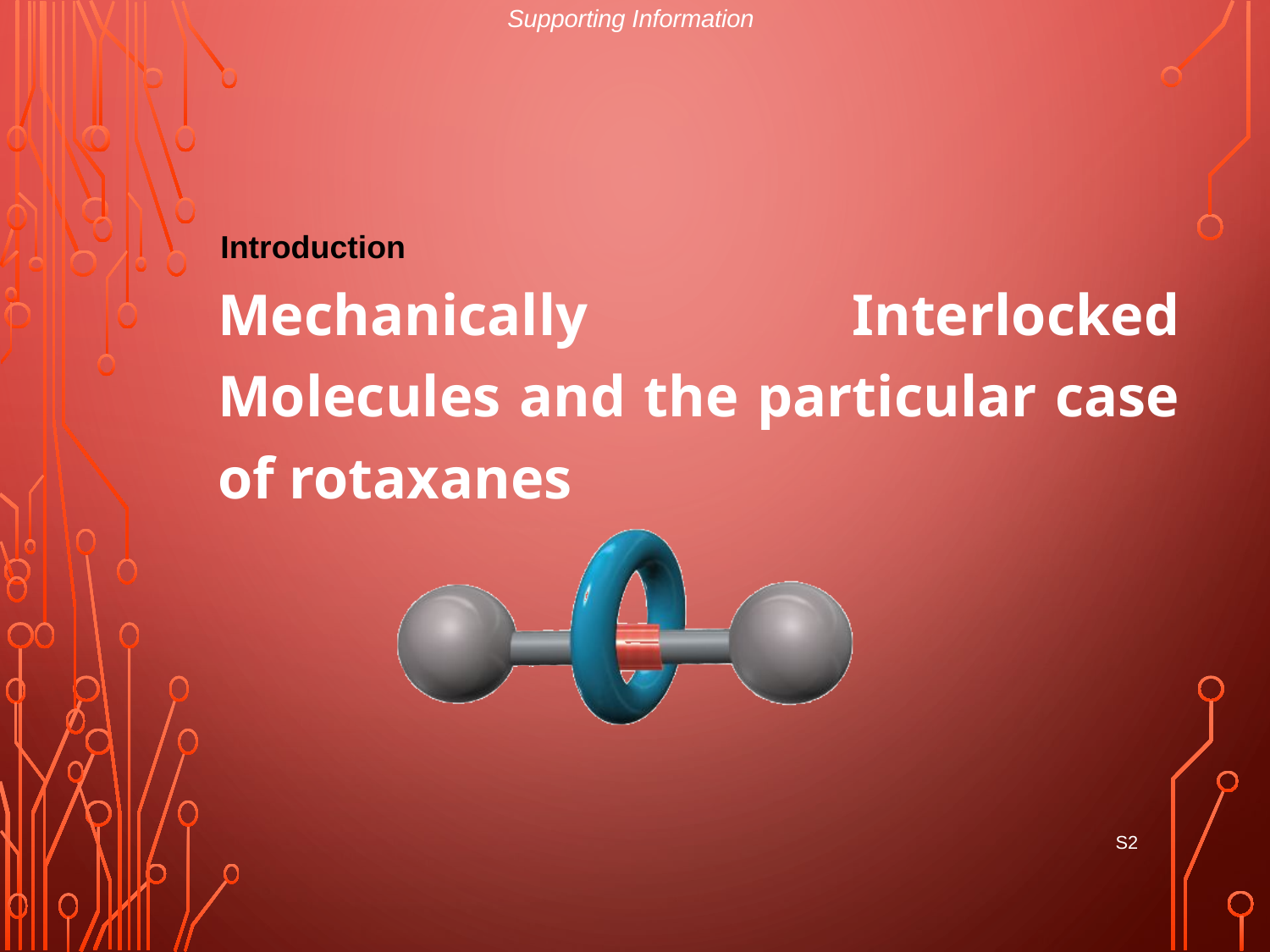

Supporting Information
Introduction
Mechanically Interlocked Molecules and the particular case of rotaxanes
S2

## Slide 3
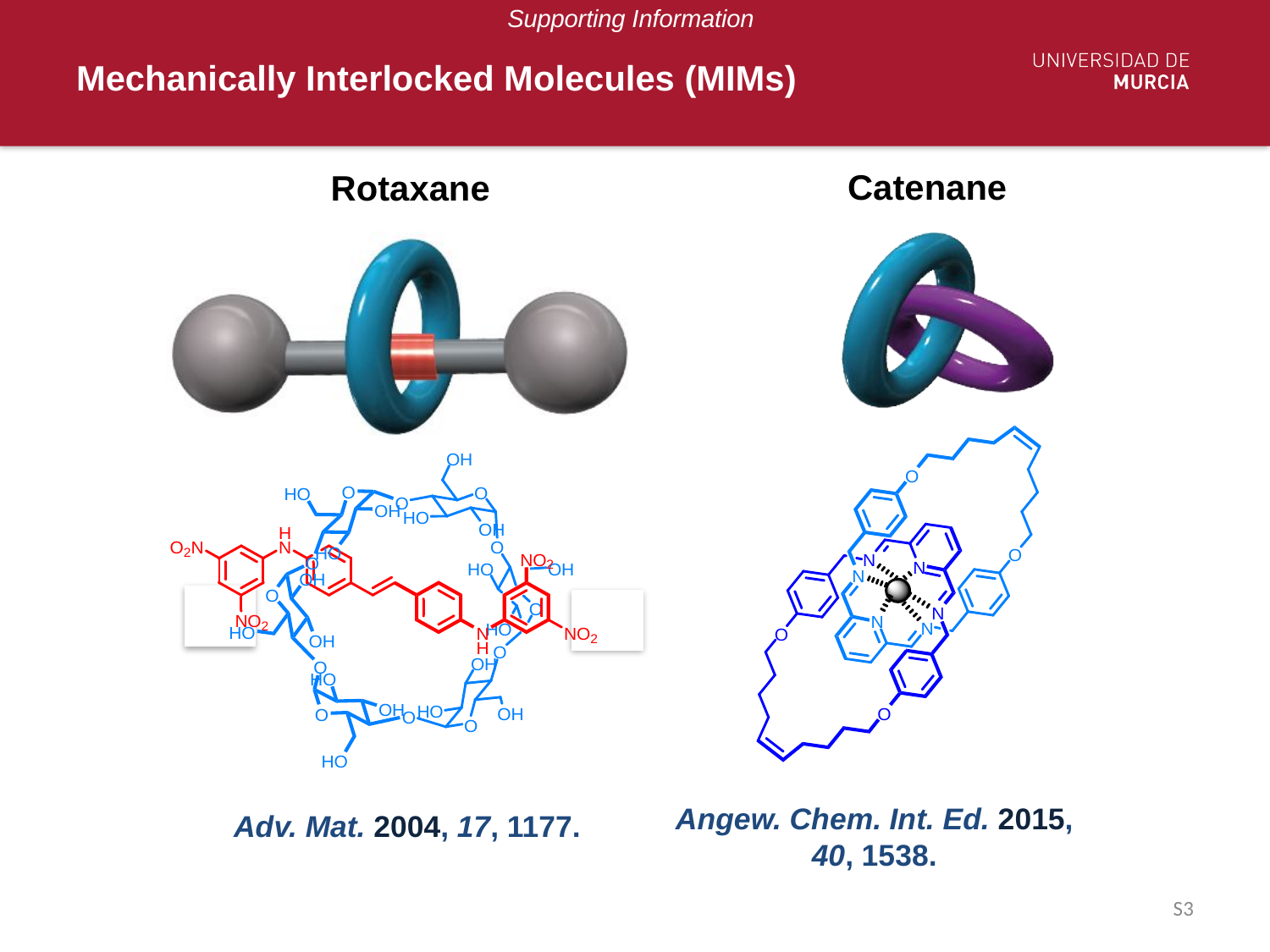

Supporting Information
Mechanically Interlocked Molecules (MIMs)
# Moléculas enlazadas mecánicamente
Catenane
Rotaxane
Adv. Mat. 2004, 17, 1177.
Angew. Chem. Int. Ed. 2015, 40, 1538.
S3

## Slide 4
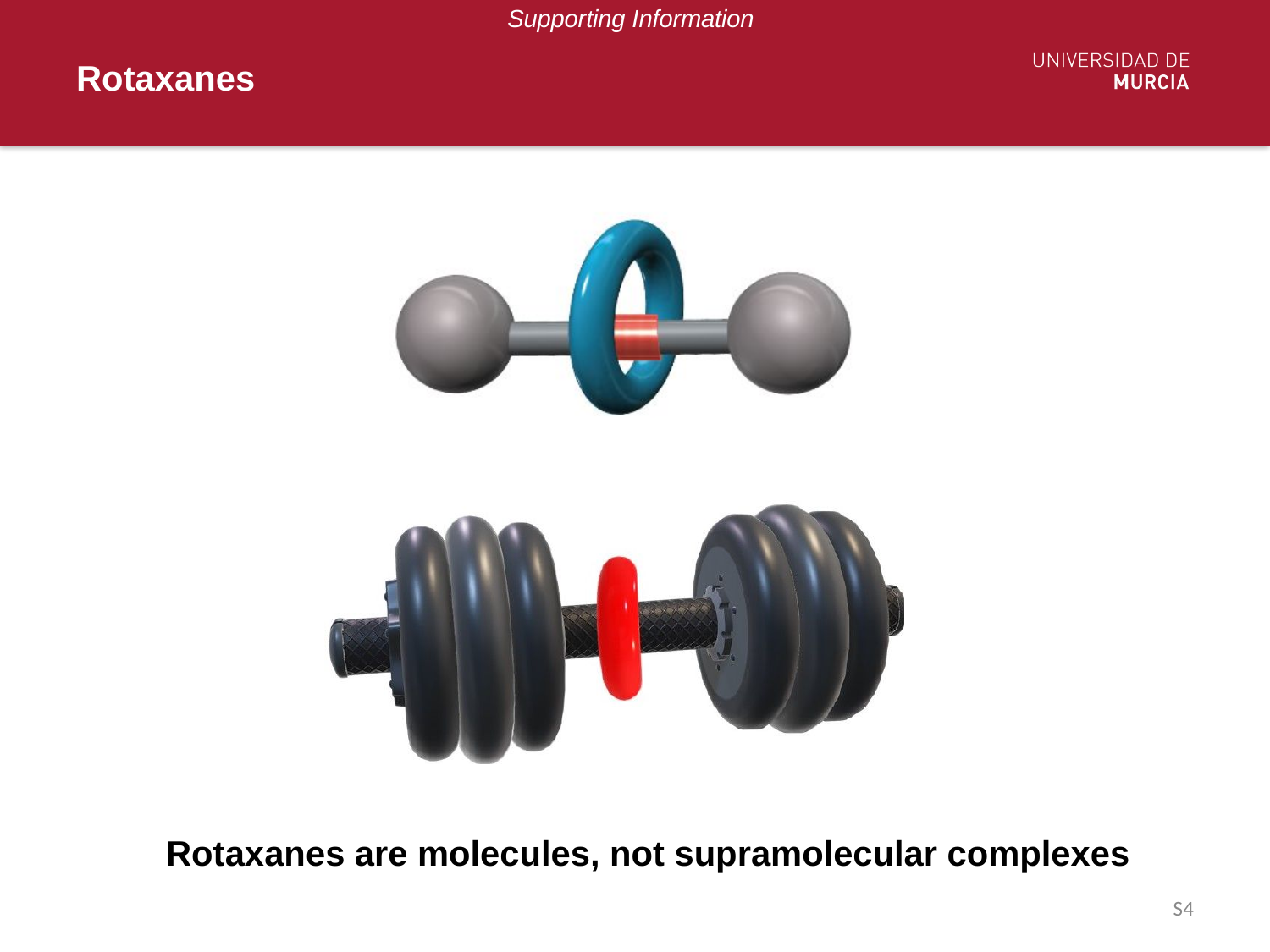

Supporting Information
Rotaxanes
# Moléculas enlazadas mecánicamente
Rotaxanes are molecules, not supramolecular complexes
S4

## Slide 5
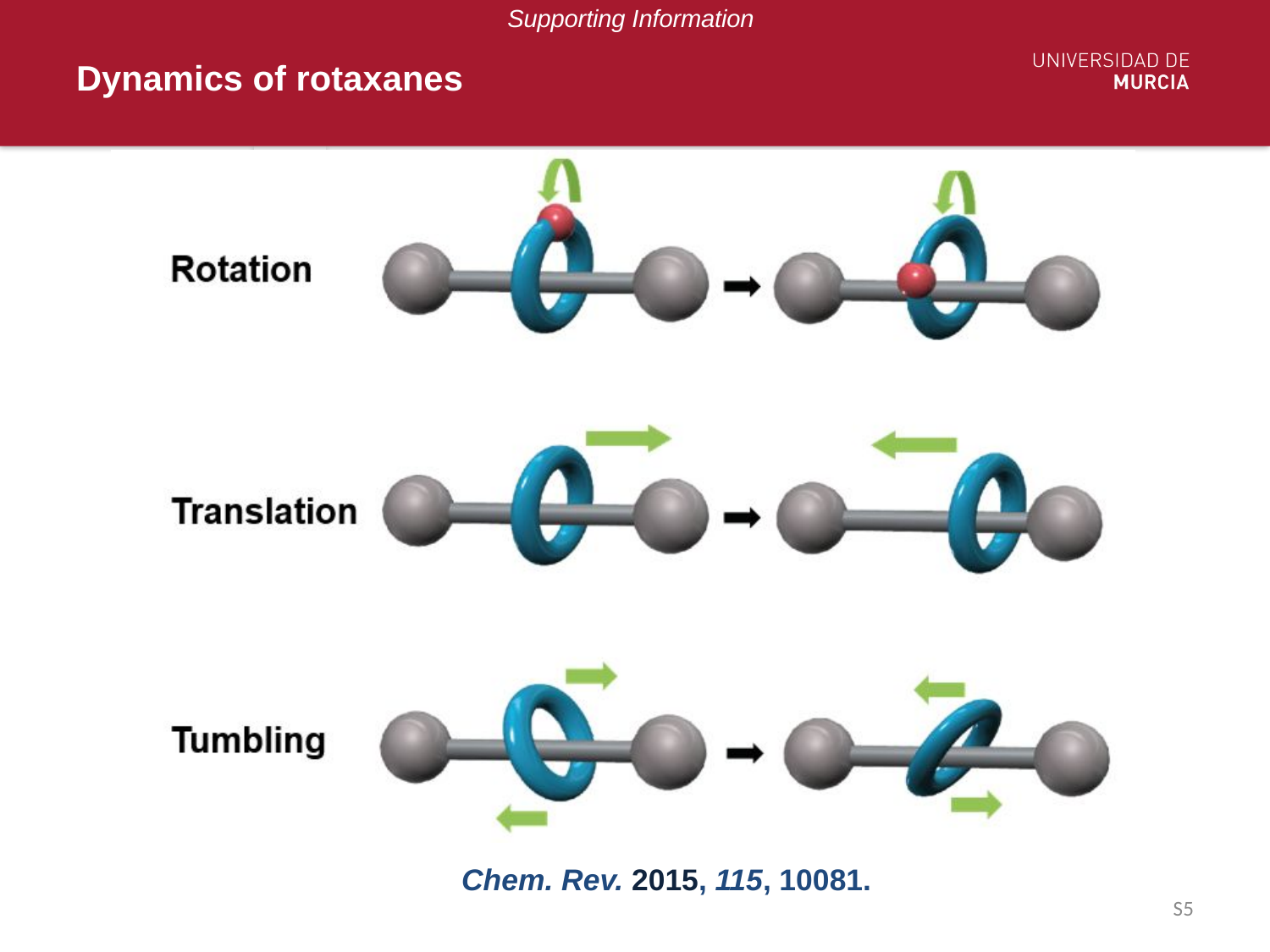

Supporting Information
Dynamics of rotaxanes
# Dinámica de rotaxanos
Chem. Rev. 2015, 115, 10081.
S5

## Slide 6
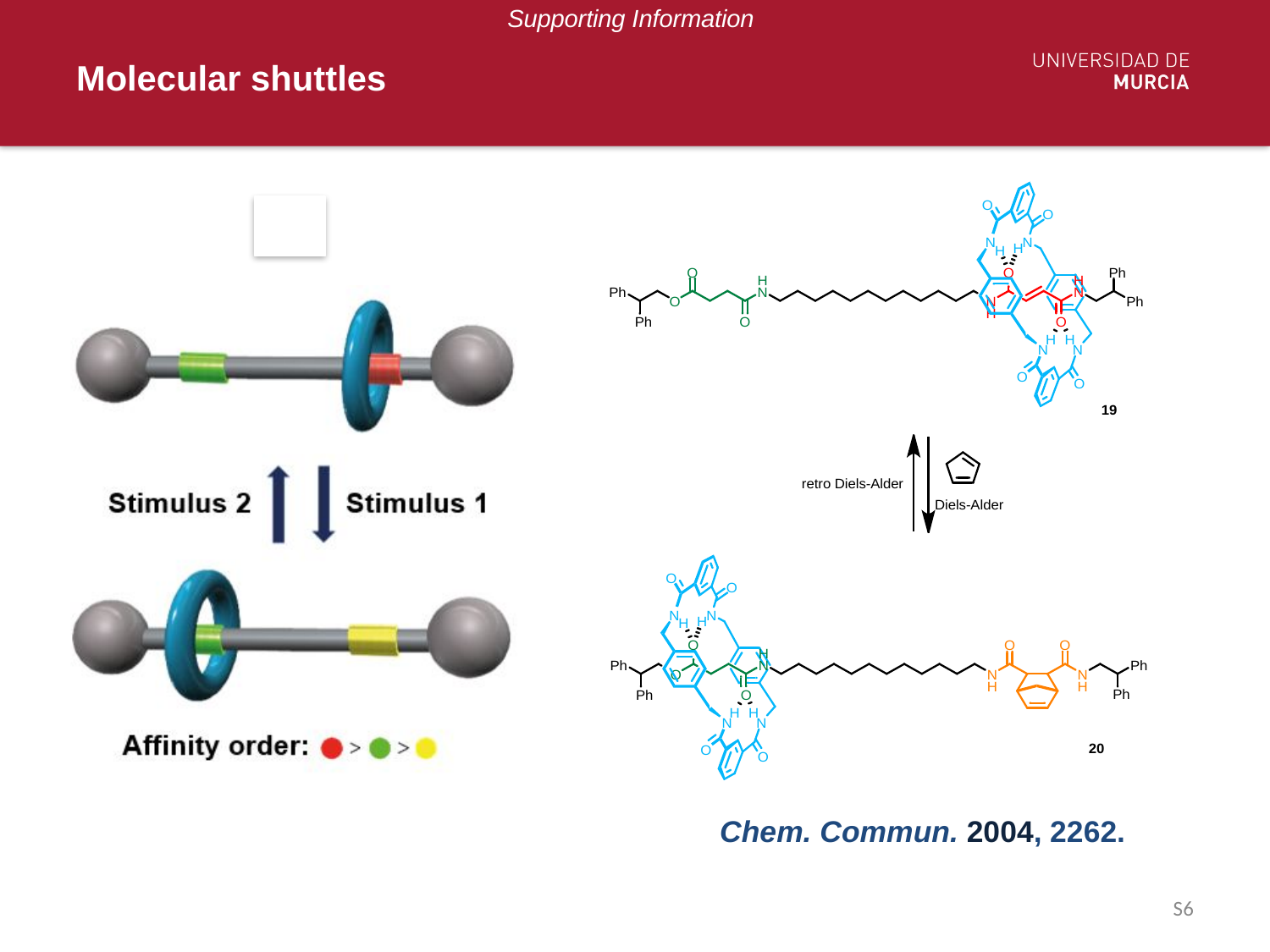

Supporting Information
Molecular shuttles
# Lanzadera molecular
Chem. Commun. 2004, 2262.
S6

## Slide 7
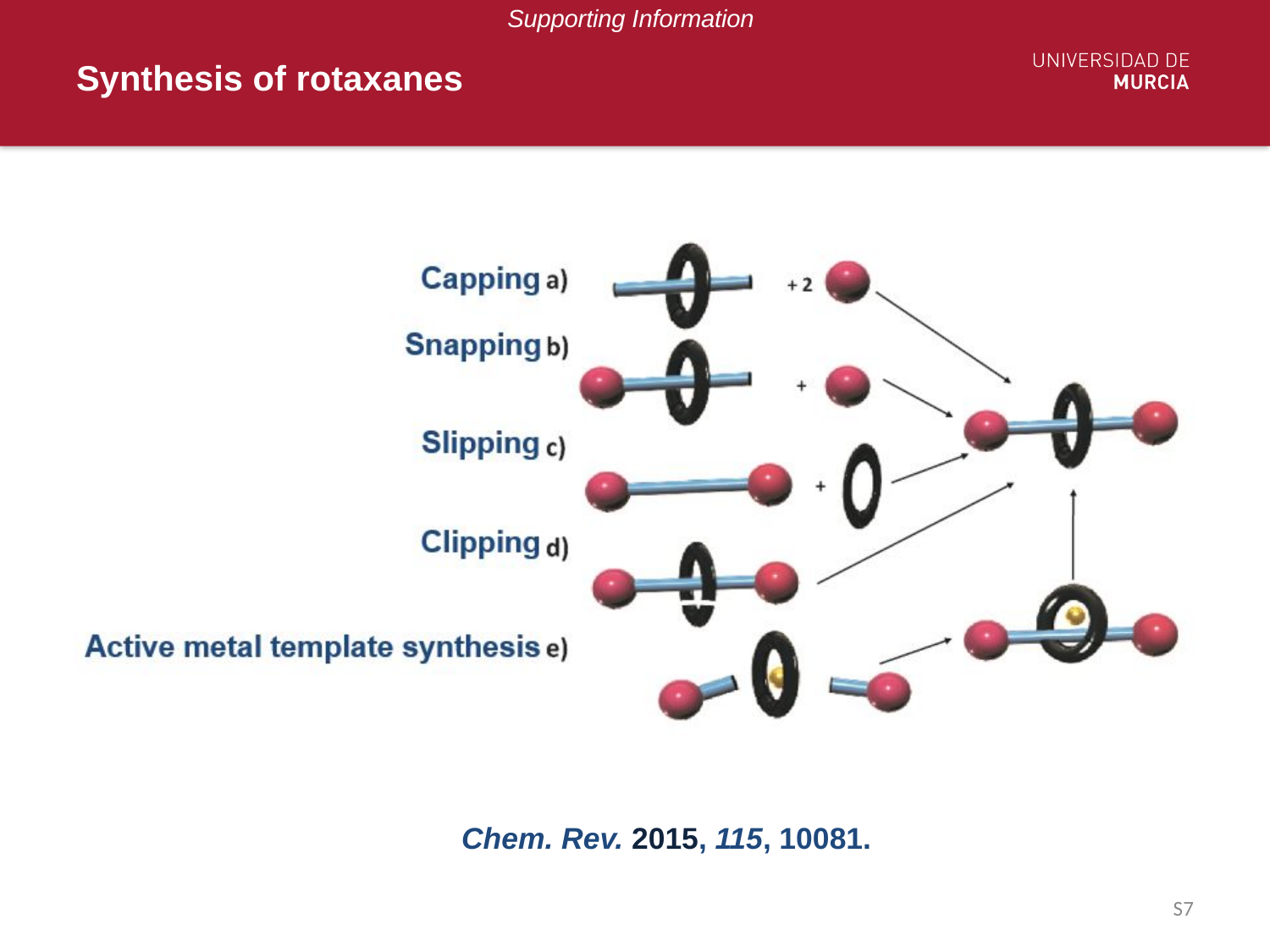

Supporting Information
Synthesis of rotaxanes
# Lanzadera molecular
Chem. Rev. 2015, 115, 10081.
S7

## Slide 8
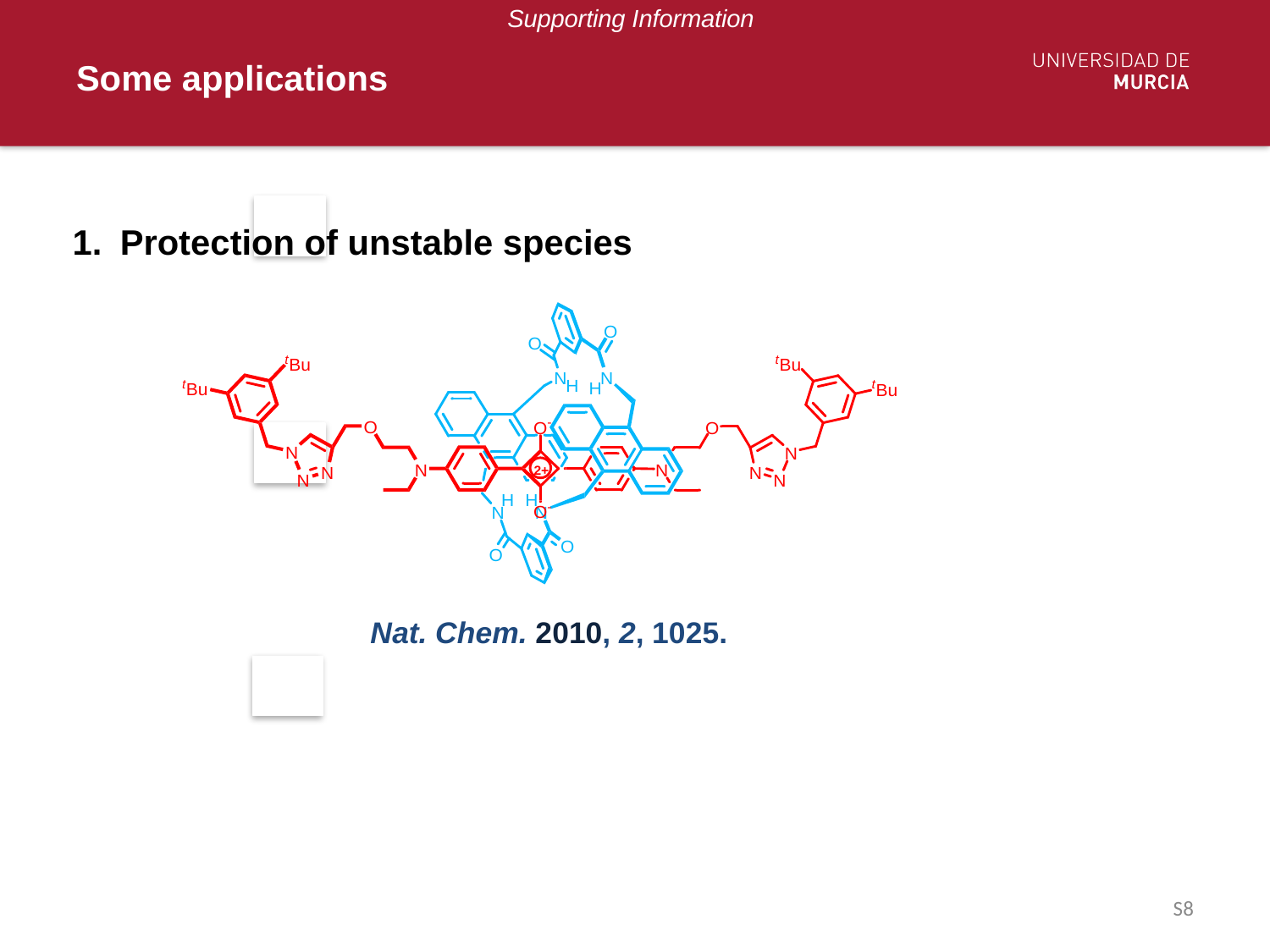

Supporting Information
Some applications
# Aplicaciones de rotaxanos
Protection of unstable species
Nat. Chem. 2010, 2, 1025.
S8

## Slide 9
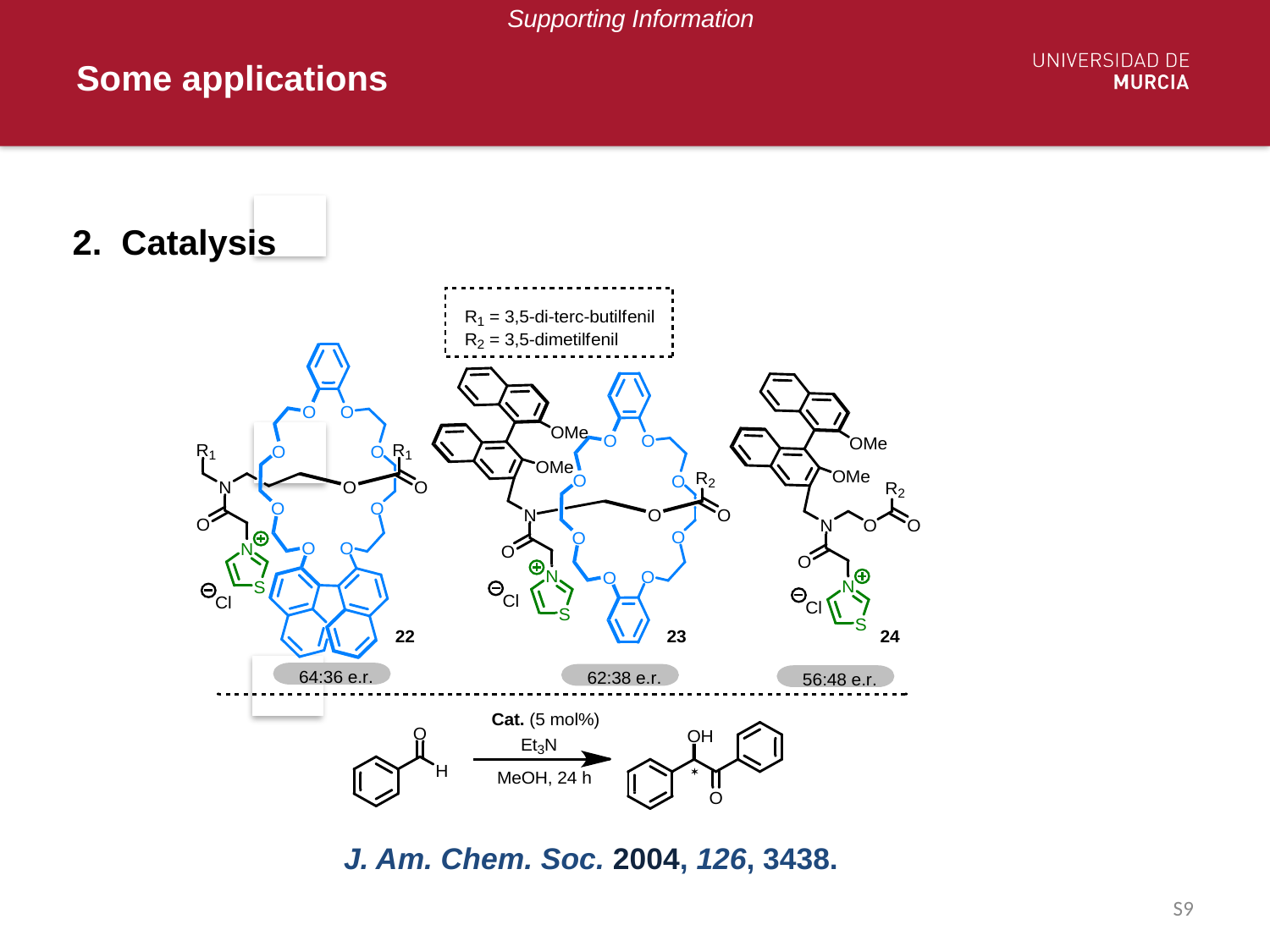

Supporting Information
Some applications
# Aplicaciones de rotaxanos
2. Catalysis
J. Am. Chem. Soc. 2004, 126, 3438.
S9

## Slide 10
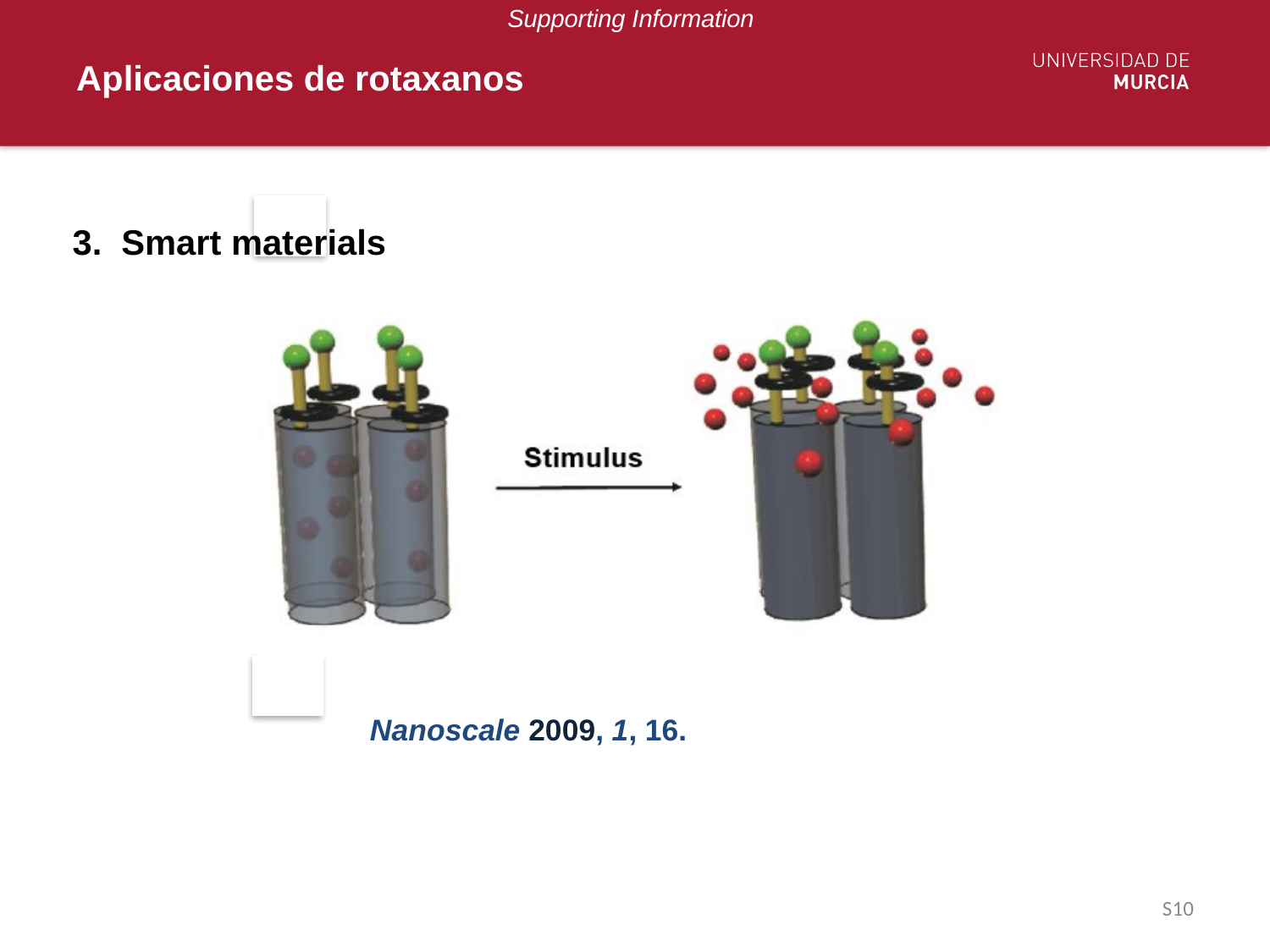

Supporting Information
Aplicaciones de rotaxanos
# Aplicaciones de rotaxanos
3. Smart materials
Nanoscale 2009, 1, 16.
S10

## Slide 11
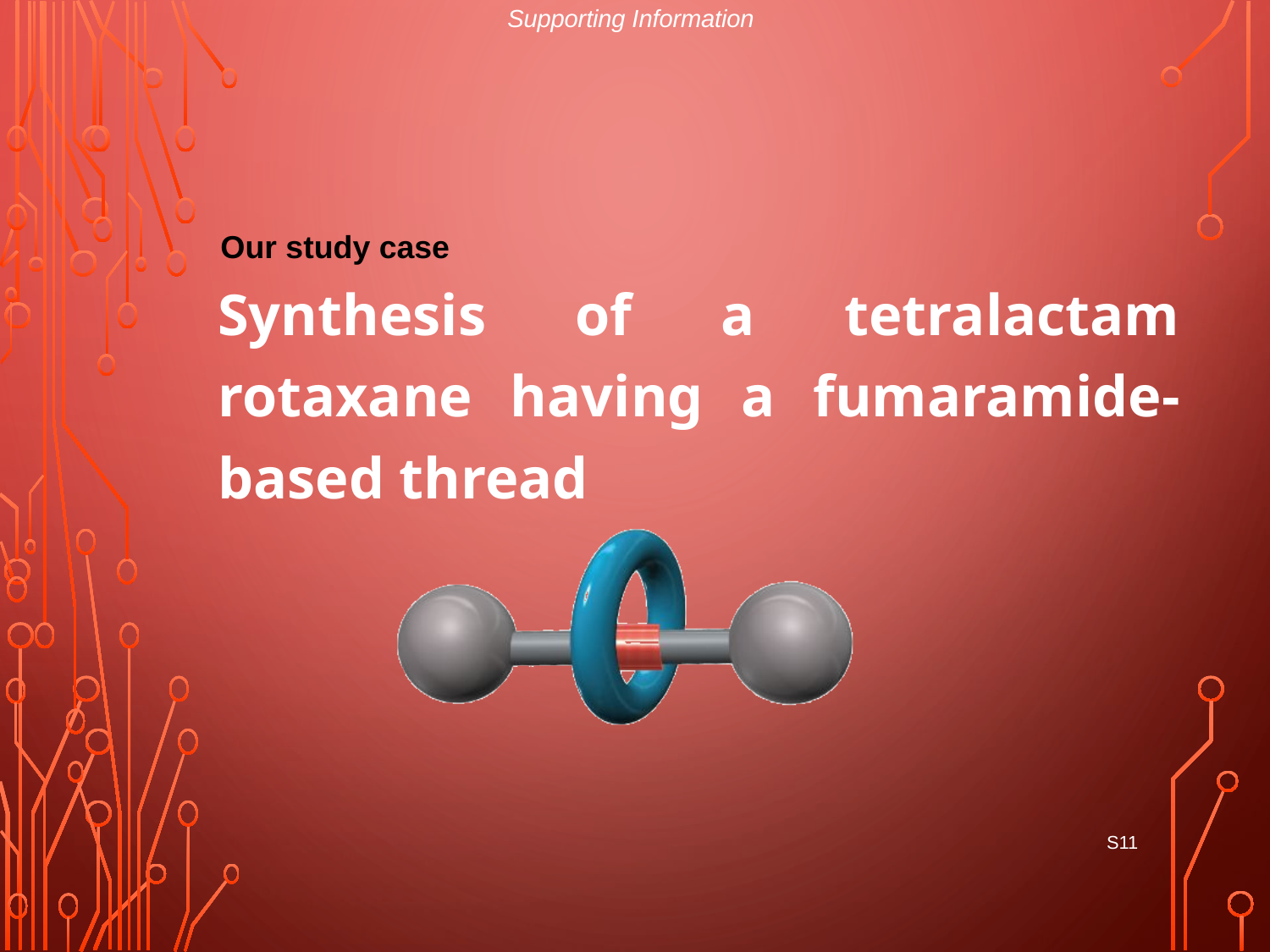

Supporting Information
Our study case
Synthesis of a tetralactam rotaxane having a fumaramide-based thread
S11

## Slide 12
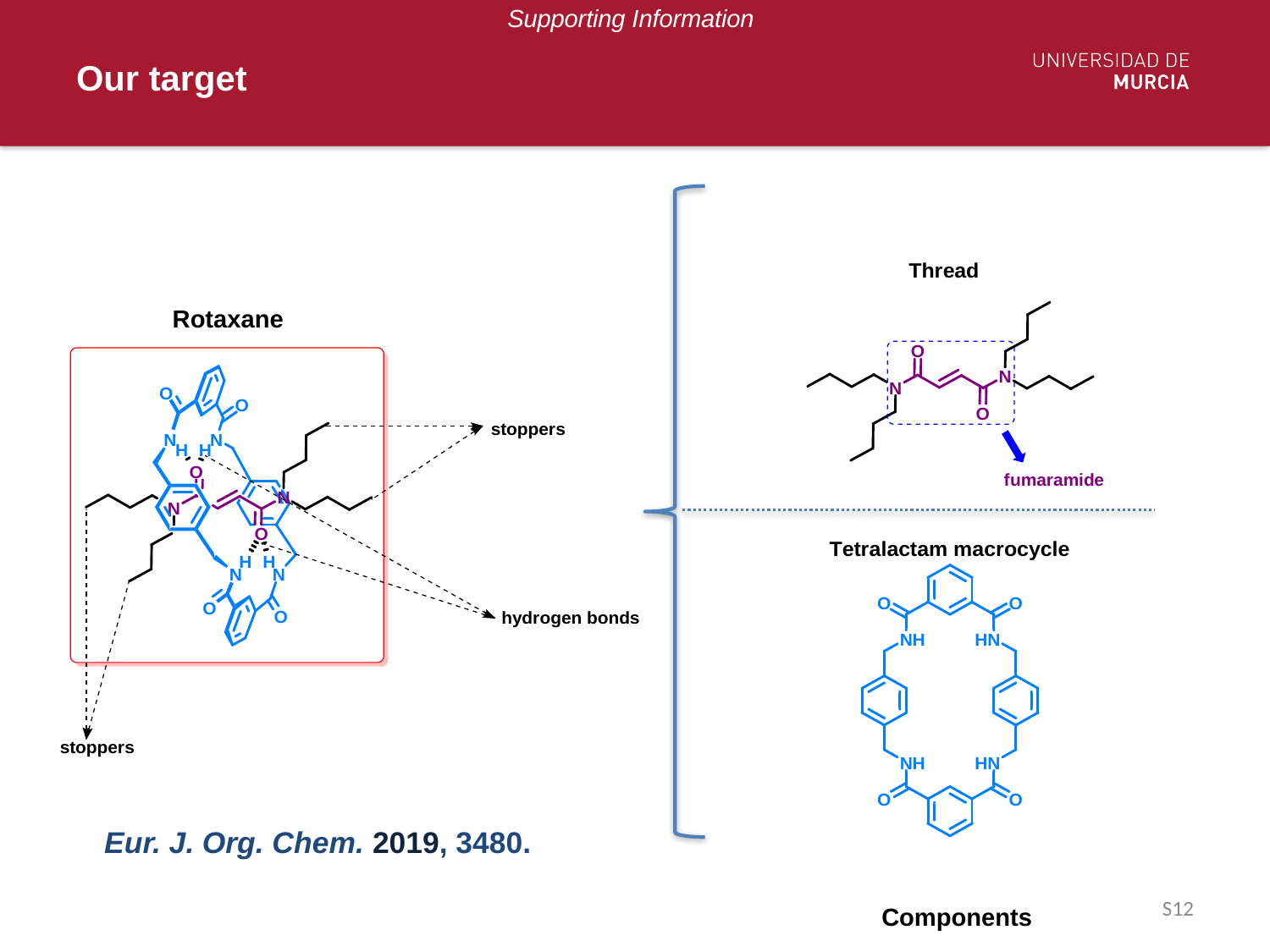

Supporting Information
Our target
# Movimiento de rotación
Rotaxane
Eur. J. Org. Chem. 2019, 3480.
Components
S12

## Slide 13
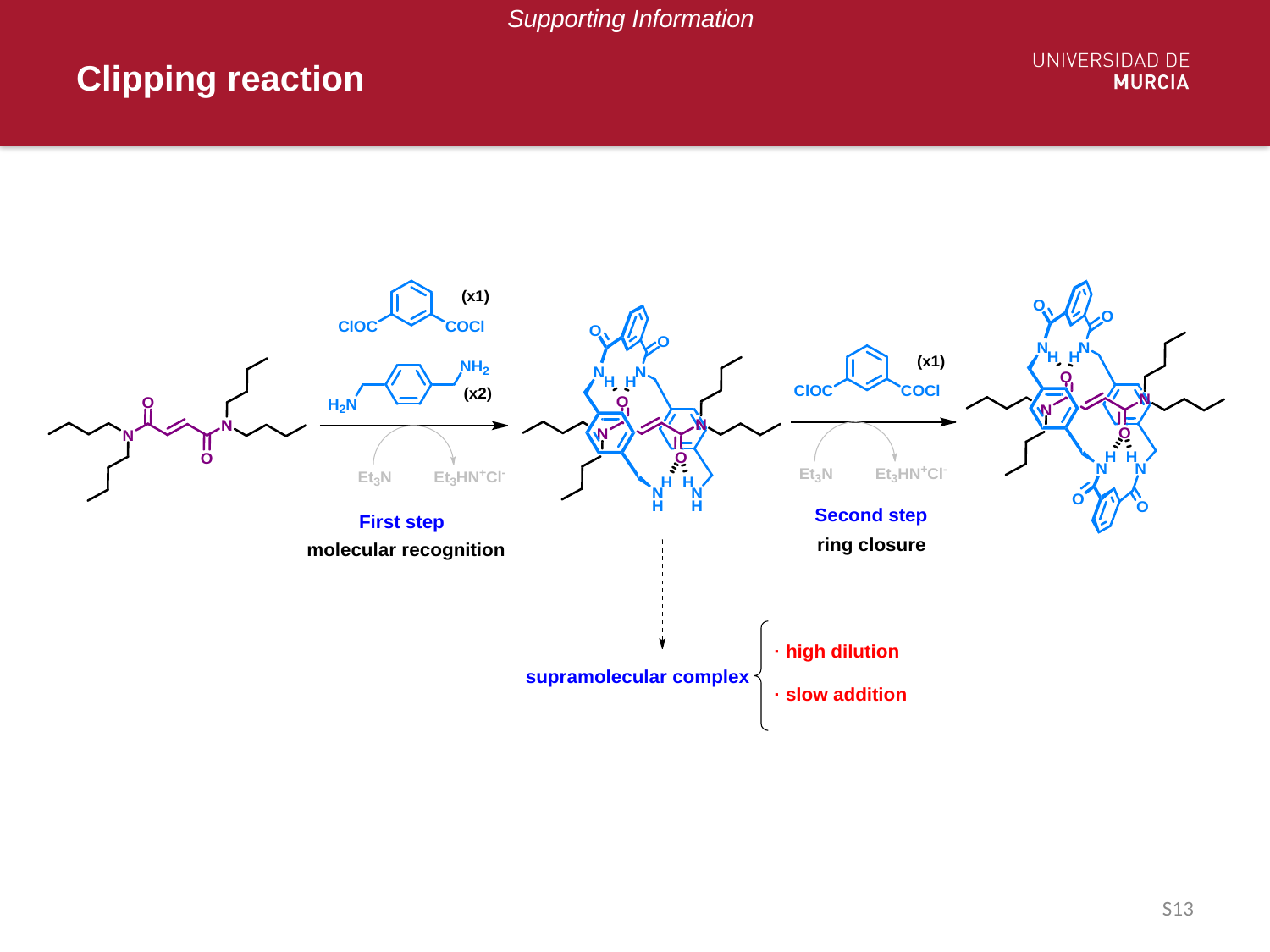

Supporting Information
Clipping reaction
# Movimiento de rotación
S13

## Slide 14
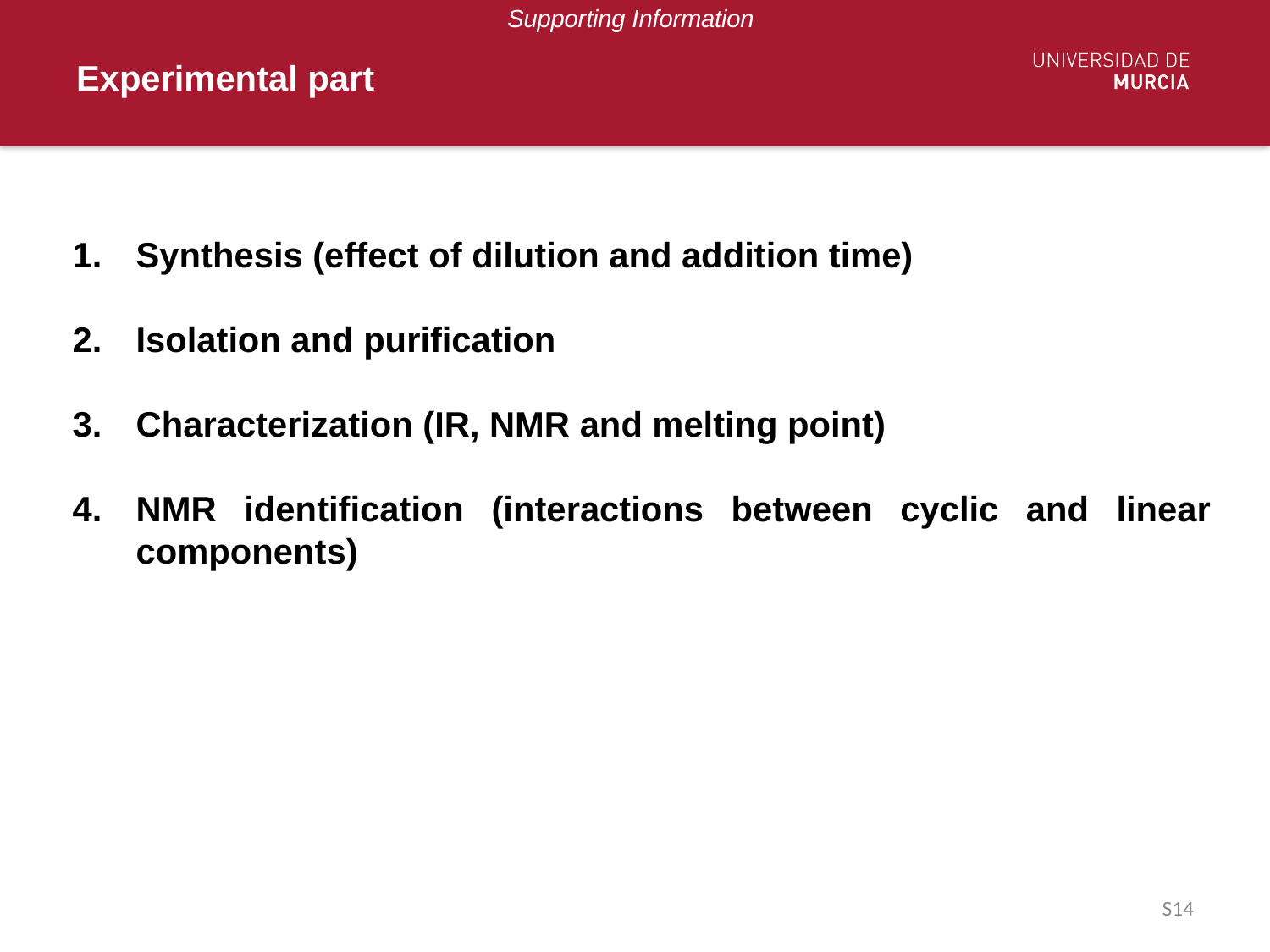

Supporting Information
Experimental part
# Movimiento de rotación
Synthesis (effect of dilution and addition time)
Isolation and purification
Characterization (IR, NMR and melting point)
NMR identification (interactions between cyclic and linear components)
S14
